# Phylogenomic Analyses and Molecular Signatures Elucidating the Evolutionary Relationships amongst the *Chlorobia* and *Ignavibacteria* Species: Robust Demarcation of Two Family-Level Clades within the Order *Chlorobiales* and Proposal for the Family *Chloroherpetonaceae* fam. nov

**DOI:** 10.3390/microorganisms10071312

**Published:** 2022-06-29

**Authors:** Sarah Bello, Mohammad Howard-Azzeh, Herb E. Schellhorn, Radhey S. Gupta

**Affiliations:** 1 Department of Biochemistry and Biomedical Sciences, McMaster University, Hamilton, ON L8N 3Z5, Canada; bellos1@mcmaster.ca; 2 Department of Population Medicine, Ontario Veterinary College, University of Guelph, Guelph, ON N1G 2W1, Canada; mhowarda@uoguelph.ca; 3 Department of Biology, McMaster University, Hamilton, ON L8S 4K1, Canada; schell@mcmaster.ca

**Keywords:** phylogenomic and comparative genomic analyses, conserved signature indels (CSIs), molecular signatures, class *Chlorobia* and the families *Chlorobiaceae* and *Chloroherpetonaceae*, *Ignavibacteria*, uncultured species/strains related to *Chlorobia*/*Ignavibacteria*

## Abstract

Evolutionary relationships amongst *Chlorobia* and *Ignavibacteria* species/strains were examined using phylogenomic and comparative analyses of genome sequences. In a phylogenomic tree based on 282 conserved proteins, the named *Chlorobia* species formed a monophyletic clade containing two distinct subclades. One clade, encompassing the genera *Chlorobaculum, Chlorobium, Pelodictyon,* and *Prosthecochloris,* corresponds to the family *Chlorobiaceae,* whereas another clade, harboring *Chloroherpeton thalassium, Candidatus* Thermochlorobacter aerophilum, *Candidatus* Thermochlorobacteriaceae bacterium GBChlB, and *Chlorobium* sp. 445, is now proposed as a new family (*Chloroherpetonaceae* fam. nov). In parallel, our comparative genomic analyses have identified 47 conserved signature indels (CSIs) in diverse proteins that are exclusively present in members of the class *Chlorobia* or its two families, providing reliable means for identification. Two known *Ignavibacteria* species in our phylogenomic tree are found to group within a larger clade containing several *Candidatus* species and uncultured *Chlorobi* strains. A CSI in the SecY protein is uniquely shared by the species/strains from this “larger *Ignavibacteria* clade”. Two additional CSIs, which are commonly shared by *Chlorobia* species and the “larger *Ignavibacteria* clade”, support a specific relationship between these two groups. The newly identified molecular markers provide novel tools for genetic and biochemical studies and identification of these organisms.

## 1. Introduction

Members of the class *Chlorobia*, also known as green sulfur bacteria, were, until recently, comprised of a group of strictly anaerobic, photosynthetic bacteria [1] that occupy a very narrow environmental niche consisting of anoxic aquatic settings in stratified lakes, where sunlight can penetrate [1,2,3,4]. These bacteria use reduced sulfur compounds as reductants and electron source to fix CO_2_ using the reverse TCA cycle [4,5,6]. Based on their branching in phylogenetic trees for the 16S rRNA gene and FMO protein sequences, these bacteria have been placed in a separate phylum *Chlorobiota*, consisting of a single class “*Chlorobia*” containing a single order *Chlorobiales* and a single family *Chlorobiaceae* [3,4,5,7,8,9,10]. According to the List of Prokaryotic Names with Standing in Nomenclature (LPSN) server [11], the family *Chlorobiaceae* contains six genera (*Ancalochloris, Chlorobaculum, Chlorobium, Chloroherpeton, Pelodictyon,* and *Prosthecochloris*) [4,5,12,13]. However, there is no information available for the sole species *Ancalochloris perfilievii* from the genus *Ancalochloris* [14] and most of the species from the family *Pelodictyon* have been transferred into the genus *Chlorobium* [12,15]. According to the LPSN server, the family *Chlorobiaceae* now contains 15 species (excluding synonyms and non-validly published species) [11]. However, most of these species were described prior to 2008 [11] and since then, except for a few *Candidatus* species [16,17], no new *Chlorobiaceae* species have been identified. Until 2013, members of the phylum *Bacteroidetes* (recently renamed *Bacteroidota*) [18] were the closest relatives of the *Chlorobia* species [3,5,12,19,20] and together with the *Fibrobacteres* they formed the *Fibrobacteres–Bacteroidetes–Chlorobia* (FBC) superphylum [20,21]. However, subsequent studies have led to the identification of two new species, viz., *Melioribacter roseus* [22,23] and *Ignavibacterium album* [7], which, based on phylogenetic trees for several gene/protein sequences, are considered the closest relatives of *Chlorobia* species [7,23,24]. Unlike the *Chlorobia* species, all of which are strictly anaerobic and obligate photolithotrophs [3,4,5,6], *Ignavibacterium* as well as *Melioribacter* are moderately thermophilic, facultatively anaerobic, and obligate chemoorganotrophs [7,23,24,25,26]. In earlier phylogenetic trees based on the 16S rRNA gene and other genes/proteins, a clade comprising *I. album* and *M. roseus* was separated from *Chlorobia* species by a long branch [7,23]. This led to the placement of these two species into a new phylum, recently renamed *Ignavibacteriota* [18], containing a single class *Ignavibacteria* and a single order *Ignavibacteriales* [23,24], which is a sister taxon of the *Chlorobia* (*Chlorobiota*) [23,24]. In addition to these two *Ignavibacteria* species, analyses of metagenomic sequences from several thermophilic habitats have led to the reconstruction of genomes for several uncultivated organisms that are related to *Chlorobia–Ignavibacteria* species [25,26,27]. Based on their genome sequences, some of these organisms are hypothesized to have biochemical–physiological characteristics overlapping with the properties of either members of the class *Chlorobia* or members of the class *Ignavibacteria* (i.e., aerobic photoheterotrophs possessing genes for bacteriochlorophyll biosynthesis and related proteins) [25,26,27].

Due to rapid advances in genome sequencing technology, and because of several major genomic sequence projects [28,29,30], whole genome sequences are now available for most of the species from the *Chlorobia*/*Ignavibacteria* groups. In addition, sequence information is also available for several unnamed *Chlorobia* species in the NCBI database (https://www.ncbi.nlm.nih.gov/genome/ (accessed on 15 January 2022) [31]. The available genomes provide a comprehensive resource for more reliably understanding the evolutionary relationships amongst these organisms based on multiple independent genomic approaches. Based on genome sequences, robust phylogenetic trees can be constructed based on a large dataset of genes/proteins, exhibiting a high degree of statistical support at different taxonomic levels [32,33,34,35]. Indeed, phylogenetic analyses based on 120 proteins that are ubiquitously found in different micro-organisms have led to the creation of a Genome Taxonomy database (GTDB), which has become an important reference resource for the classification of prokaryotic organisms [32,36]. In the GTDB taxonomy (https://gtdb.ecogenomic.org/ (accessed on 30 May 2022)), members of the classes *Chlorobia* and *Ignavibacteria* exhibit considerable genetic diversity, indicating that more detailed studies are needed to clarify the evolutionary relationships and taxonomy of these organisms.

With the aim of clarifying the evolutionary relationships amongst *Chlorobia*/*Ignavibacteria* species, we have carried out phylogenomic and comparative genomic studies on the genome sequences of these species. To this end, we have constructed phylogenetic trees for these two groups of species based on concatenated sequences for several sets of conserved proteins. These trees, along with the GTDB taxonomy [32] and phylogenetic trees reported in earlier studies [25,26,27], provide a phylogenetic framework for understanding the evolutionary relationships among the *Chlorobia* species and their relationship to the class *Ignavibacteria*. In parallel, we have also performed extensive studies on protein sequences from the *Chlorobia/Ignavibacteria* genomes to identify molecular signatures, consisting of conserved signature indels (CSIs), that are uniquely shared by species from the major clades of *Chlorobia/Ignavibacteria* observed in our phylogenetic trees. Molecular synapomorphies, such as CSIs, that are specific for a given group of organisms, provide strong evidence, independently of phylogenetic trees, for the genetic cohesiveness and common ancestry of a given group of organisms [21,37,38,39,40,41]. Hence, these molecular markers provide reliable means for the delineation of specific clades in molecular terms, and they have proven useful for taxonomic purposes [35,39,42,43]. Results presented here show that the named *Chlorobia* species (order *Chlorobiales*) form a strongly supported clade in trees based on different genes/proteins. The distinctness of this clade is independently strongly supported by 33 CSIs that are present in diverse proteins that are uniquely shared by the members of this class/order. Furthermore, members of the class *Chlorobia* (order *Chlorobiales*) form two distinct clades, which can be reliably distinguished from each other based upon several identified CSIs that are exclusively shared by the members of these two clades. One of these clades, encompassing all *Chlorobiales* genera except *Chloroherpeton*, corresponds to the revised family *Chlorobiaceae*, whereas a second clade grouping together *Chloroherpeton thalassium* and some *Candidatus* Thermochlorobacter strains/isolates and an uncultured *Chlorobium* sp. 445 (we will be referring to this group as thermophilic photoheterotrophs), is now proposed as a new family (*Chloroherpetonaceae* fam. nov). Results presented here also show that the class *Ignavibacteria* is genetically highly diverse and that several uncultured species, currently referred to as “*Chlorobi* bacterium” as well as some *Candidatus* species, are also related to this class/phylum of bacteria. Our work has also identified two new CSIs, which are uniquely shared by most of the *Chlorobia* and *Ignavibacteria* species, providing further evidence that these two groups of organisms are closely related.

## 2. Materials and Methods

### 2.1. Construction of Phylogenetic Trees

Protein sequences were downloaded for different available genomes from named *Chlorobia* species as well as several unnamed/uncultured *Chlorobi* species that are denoted by specific numbers in the NCBI genome database [31]. In addition, the sequences for *I. album* and *M. roseus* and some *Candidatus* species (viz., Cand. *Thermochlorobacter aerophilum* [27], Cand. *Thermochlorobacteriaceae* bacterium GBChlB, Cand. *Kapabacteria thiocyanatum*, and Cand. *Kryptonium thompsoni*), which according to the GTDB taxonomy are related to the *Chlorobia*/*Ignavibacteria* taxa, were also downloaded. In addition, genome sequences for several *Bacteroidetes/Fibrobacteres* species (viz., *Rhodothermus* (R.) *marina, Salinibacter* (S.) *ruber*, *Cytophaga* (C.) *aurantiaca, Bacteroides* (B.) *fragilis,* and *Fibrobacter* (F.) *succinogenes*) were also downloaded to serve as outgroups in phylogenetic analysis. Using these genome sequences, a phylogenetic tree was constructed based on concatenated sequences of 282 conserved proteins that are a part of the phyloeco marker set for the FBC superphylum [20]. Two additional phylogenetic trees were constructed for these species based on the concatenated sequences for Gyrase A and Gyrase B proteins, as well as UvrD and PolA proteins, which are highly conserved proteins commonly employed for phylogenetic analysis [38].

The construction of the phylogenetic trees was done using an internally developed pipeline described by Adeolu et al. (2016) [40]. Briefly, the CD-HIT program and the profile Hidden Markov Models (HMMs) of the proteins that are part of the FBC–phyloeco set [34] were used to search for homologs of these proteins in the input genomes. The search parameters used required that the selected homologs of different proteins shared a minimum of 50% sequence identity and sequence length, and they were found in at least 80% of the input genomes. The Clustal Omega algorithm [44] was used to generate multiple sequence alignments (MSAs) of these protein families. The aligned protein families were trimmed with TrimAl [45] to remove poorly aligned regions before concatenation of the other core proteins. The final concatenated sequence alignments of these proteins used for tree construction consisted of 89,743 aligned amino acids. Based on this sequence alignment, an initial tree was constructed using FastTree 2 [46] based on the Whelan and Goldman model of protein sequence evolution [47] and optimized using RAxML 8 [48] based on the Le and Gascuel model of protein sequence evolution [49]. SH-like statistical support values (which are similar to bootstrap scores) were calculated for each branch node using RAxML 8 [48]. The resultant phylogenetic tree was drawn using MEGA X [50]. Sequence alignment of the 282 core proteins was also used to calculate the pairwise average amino acid identity (AAI) [51] for the species belonging to the order *Chlorobiales*. In addition, based on genome sequences for the *Chlorobia* species, the pairwise percentage of conserved proteins (POCP) between different species was also determined [52,53].

We also constructed a 16S rRNA gene tree for the *Chlorobia* and *Ignavibacteriae* species based on sequences obtained from the SILVA ribosomal RNA [54] and the NCBI genome database (https://www.ncbi.nlm.nih.gov/ (accessed on 25 March 2022)). The sequences were aligned using the MUSCLE program in MEGA-X [50]. The non-conserved regions as well as regions with gaps were removed, leaving 1269 aligned positions in the final dataset. A maximum-likelihood phylogenetic tree based on this dataset was created using MEGA X [50], employing the Tamura–Nei model [55] based on 100 bootstrap replicates.

### 2.2. Identification of Conserved Signature Indels

The identification of conserved signature indels (CSIs) was carried out as described in earlier work [56,57]. Briefly, BLASTp searches using the NCBI non-redundant database were carried out on all proteins from the genomes of *Chlorobium limicola* and *Chloroherpeton thalassium*. Based on these BLASTp searches, protein sequences were obtained for 8–10 divergent *Chlorobia/Ignavibacteria* species (generally including representative species from all three main groups of interest, i.e., *Chlorobiaceae*, *Chloroherpeton,* and *Ignavibacteria*) as well as 8–10 species from other bacterial taxa (generally belonging to different families/orders of *Bacteroidetes*). The multiple sequence alignments (MSAs) of different proteins were created using ClustalX 2.1. However, other programs can also be used for the creation of multiple sequence alignments and will yield similar results [56,57] (unpublished results). Multiple sequence alignments were visually examined for insertions or deletions of fixed length that were present in conserved regions (i.e., flanked on both sides by minimally 4–5 conserved/identical amino acids (aa) in the neighbouring 40–50 aa) and shared by only the *Chlorobia/Ignavibacteria* species. The query sequences of interest containing the identified conserved indels and their flanking 30–50 aa (generally beginning and ending with a stretch of completely conserved amino acid residues) were reblasted against the NCBI non-redundant (nr) database and the top 250–500 hits were examined. Based on these BLASTp searches, conserved indels that were specifically shared by all or most of the species from the different main clades of *Chlorobia/Ignavibacteria* were identified and further formatted using SIG_CREATE and SIG_STYLE programs (available from Gleans.net (accessed on 25 March 2022) [56]. Due to space constraints, sequence information is presented in the main figures for only a limited number of representative species. However, unless otherwise stated, the CSIs described here are shared by and are exclusive to the indicated groups of *Chlorobia/Ignavibacteria* and absent in all other bacterial homologues in the top 250–500 BLASTp hits examined. More detailed information for different CSIs is provided in the Supplemental Figures.

## 3. Results

### 3.1. Phylogenetic Analysis of the Chlorobia/Ignavibacteria Species Based on Genome Sequences

**Figure 1:**
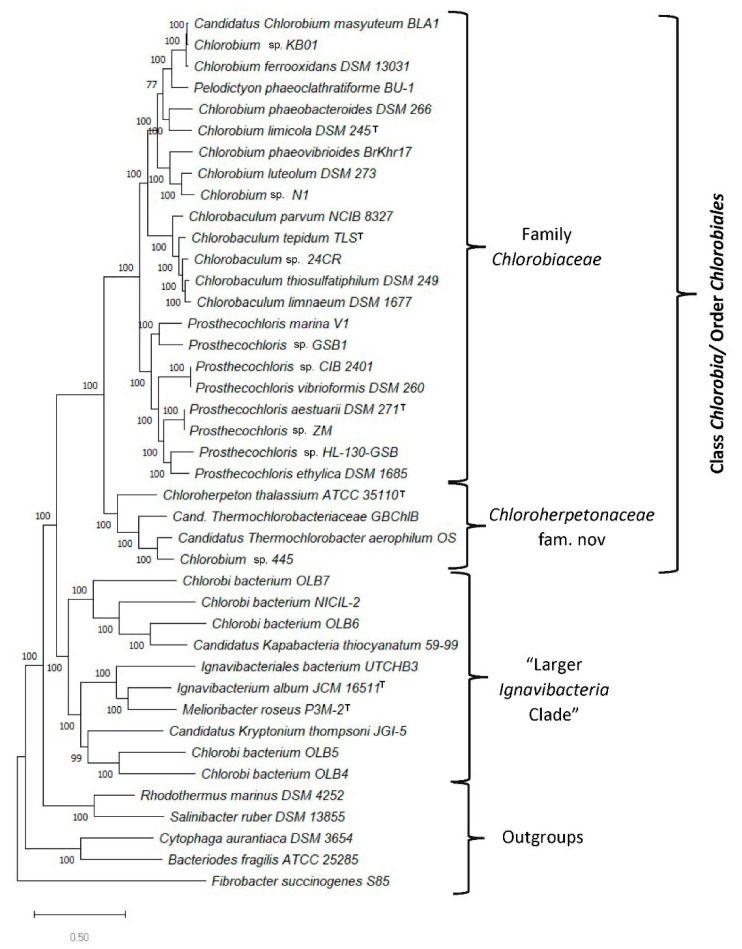
A bootstrapped maximum-likelihood tree for different genome-sequenced *Chlorobia/Ignavibacteria* and related species/strains based on concatenated sequences for 282 conserved proteins that are core proteins for these species. Statistical support values for different branches are indicated on the nodes. The tree was rooted using the sequences from the species *Fibrobacter succinogenes.* The type strains of different species are marked by the superscript^T^. Some species clades observed in this tree are marked.

The genome sequences for 36 available *Chlorobia/Ignavibacteria* species in the NCBI database were used to construct a rooted maximum-likelihood phylogenetic tree for these organisms based on concatenated sequences for 282 conserved proteins. The proteins used for tree construction are from the phyloeco set for the FBC group of bacteria and they are conserved and widely distributed in these bacteria [34]. The tree also includes sequences for some outgroup species (viz., *R. marina*, *S. ruber*, *C. aurantiaca,* and *B. fragilis*) and it was rooted using the sequence for *F. succinogenes.* The resulting maximum-likelihood distance tree based on 100 bootstrap replicates is shown in Figure 1. All major nodes in this phylogenomic tree are supported by 100% SH-support values (like bootstrap values), indicating that the evolutionary relationships observed here are robust.

In addition to the tree shown in Figure 1, we have also constructed phylogenetic trees for these species using concatenated sequences for GyrA and GyrB proteins as well as PolA and UvrD proteins. The results for these are shown in Supplementary Figures S1 and S2. The branching pattern as well as the grouping of species into different clades in these two trees are identical to that seen in Figure 1.

**Figure 2:**
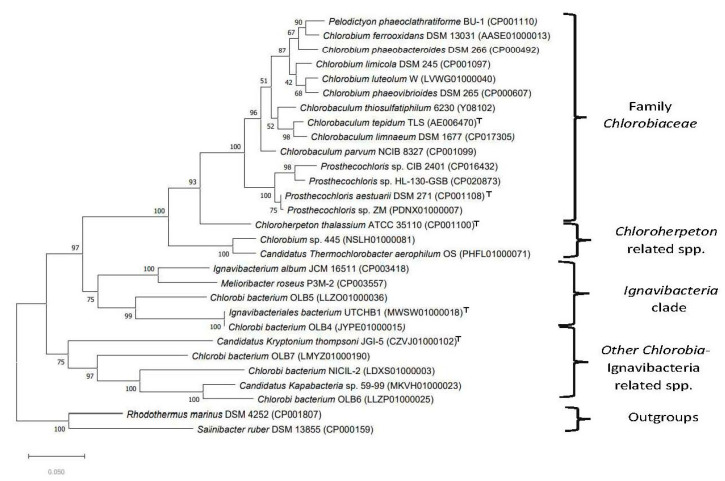
Maximum-likelihood phylogenetic tree based on 16S rRNA gene sequences for the type strains of all *Chlorobi* species. The tree was rooted using the sequences for *S. ruber* and *R. marinus*. Accession numbers of the 16S rRNA gene sequences are given within the bracket after species names in the tree. The main identified clades in this tree are marked.

The branching pattern of species shown in Figure 1 (as well as Figures S1 and S2) is similar to that reported by Roy et al. [25]. Based on this tree, several inferences regarding the evolutionary relationships among the *Chlorobia/Ignavibacteria* species/strains can be drawn. First, all named *Chlorobia* species formed a strongly supported clade (labeled the class *Chlorobia* or the order *Chlorobiales* clade), which is separated from a clade harboring *I. album*, *M. roseus,* and several other uncultured species/strains by a long branch. Second, the *Chlorobiales* clade comprises several distinct clades. One of these subclades groups together species from the genera *Chlorobaculum, Chlorobium, Pelodictyon,* and *Prosthecochloris*, whereas the second subclade consists of the species *Chloroherpeton thalassium* [58] and three uncultured thermophilic photoheterotrophic organisms related to *Candidatus* Thermochlorobacter aerophilum [25,27]. We have designated these two clades as the family *Chlorobiaceae* and the family *Chloroherpetonaceae* fam. nov., respectively. Third, within the *Chlorobiaceae* clade, species from the genera *Chlorobaculum* and *Prosthecochloris* group together, supporting the monophyly of these taxa. However, the genus *Chlorobium* is not monophyletic due to branching within *Pelodictyon phaeoclathratiforme,* indicating that this latter species is misclassified [12]. Fourth, the second major clade observed in Figure 1 consists of the species *I. album* and *M. roseus*, two *Candidatus* species (viz., Cand. Kapabacteria thiocyanatum [59] and Cand. Kryptonium thompsoni [60]), and several uncultured organisms annotated as *Chlorobi* bacterium (OLB4, OLB5, OLB6, OLB7, and NICIL-2). We have designated this clade as the “larger *Ignavibacteria* clade”. Unlike the *Chlorobia* species clade, this larger *Ignavibacteria* clade shows greater genetic diversity, and it is made up of several subclades separated by short branches. We also constructed a phylogenetic tree for *Chlorobia/Ignavibacteria* species/strains based on 16S rRNA gene sequences (Figure 2).

Unlike the phylogenomic tree (Figure 1), the 16S rRNA gene tree is characterized by lower bootstrap scores for several branches. In spite of its somewhat poor resolution, all named *Chlorobia* species formed a distinct clade within this tree. Within this clade, the clade corresponding to the family *Chlorobiaceae* is strongly supported. However, unlike the core protein tree, this tree did not group together *C. thalassium* with *Candidatus* Thermochlorobacter aerophilum and *Chlorobium* sp. 445 and the latter two species branched more deeply than *C. thalassium.* Similar branching of *C. thalassium* and thermophilic phototrophic organisms has also been previously observed [25]. However, the 16S rRNA sequences from thermophilic organisms have higher G+C content, which may, artefactually, lead to more deeper branching of thermophilic organisms in the phylogenetic tree [61]. Additionally, in the 16S rRNA tree a clade grouping the two *Ignavibacteria* species with several unnamed *Chlorobi* bacterium (OLB4, OLB5, OLB6, OLB7, and NICIL-2) and the two *Candidatus* species was not observed. These strains/species instead formed several clusters some with very long branches, branching at different positions in between the clades for *Chlorobia* and outgroup species.

**Figure 3:**
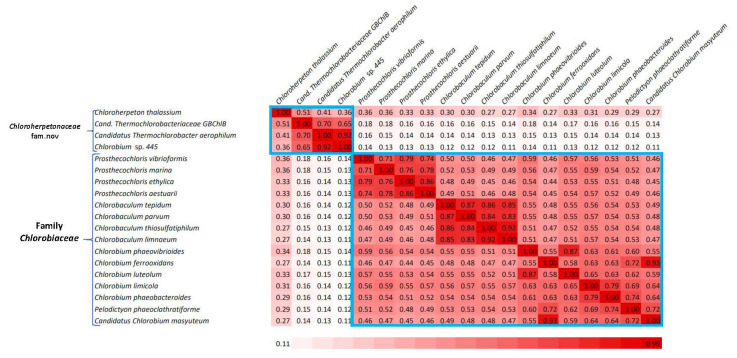
Pairwise comparison matrix showing the averages of the percentage of conserved proteins (POCP) between different genome-sequenced *Chlorobia* species. Genome pairs sharing higher POCP values are shaded more darkly (red). The regions of the matrix corresponding to the species from the two families are labeled.

Genome sequences for *Chlorobia* species were also used to calculate a pairwise matrix of percentage of conserved proteins (POCP) between different species/genomes. The POCP provides a whole-genome-based method for assessing the similarity and differences between species from related taxa [52,53]. It has been suggested that a POCP matrix is more useful than an average amino acid identity (AAI) matrix for discrimination of taxa at or above the genus rank [53]. In Figure 3, we show the POCP matrix for members of the class *Chlorobia*.

As seen from this matrix, based on the POCP values, different species/strains from the order *Chlorobiales* are clearly separated into two clades corresponding to the families *Chlorobiaceae* and *Chloroherpetonaceae*. In terms of POCP values, the members of these two clades show no overlap and they are thus clearly distinct. In addition, a matrix based on pairwise AAI values between different *Chlorobia* species was also calculated based on core proteins using an internally developed pipeline [40]. This matrix is shown in Figure S3. While the AAI values for species within these two families were in the range of 0.72–0.87 (for *Chlorobiaceae*) and 0.65 −0.80 (for *Chloroherpetonaceae*), the AAI values for interfamily comparisons ranged from 0.67 to 0.70. Although there is no established threshold value for the demarcation of family-level taxa based on AAI [51], the observed differences between the intrafamily and interfamily AAI values supported the overall distinction between the members of these two family-level clades.

### 3.2. Identification of Molecular Markers Specific for the Main Clades of Chlorobia Species

**Figure 4:**
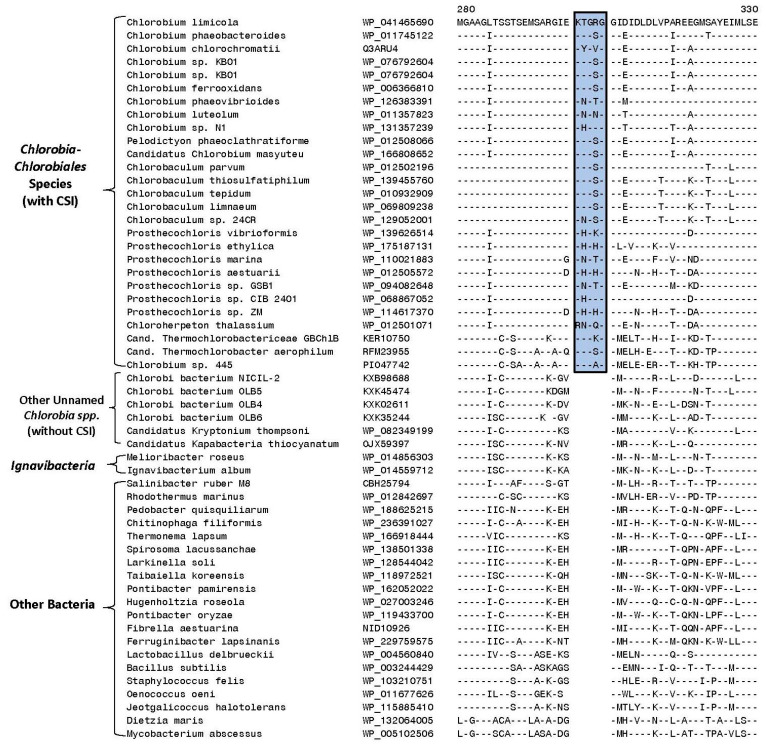
Partial sequence alignment of the protein phosphoribosylformylglycinamidine synthase II showing a five-amino-acid insertion (boxed) in a conserved region that is exclusively shared by all species/strains from the class *Chlorobia* (Figure 1). This insert is lacking in *Ignavibacteria* as well as other *Candidatus* and *Chlorobi* bacteria strains that do not group with the *Chlorobia* clade. The dashes (-) in this and all other sequence alignments indicate identity with the amino acids on the top line. Gaps in sequence alignment indicate that no amino acid is present in that position. Accession numbers for different sequences are indicated in the second column and the position of this sequence fragment within the protein is indicated above the sequences.

Results of our phylogenomic studies show that the named *Chlorobia* species form a strongly supported clade. Furthermore, within it, two family-level clades are observed. However, several uncultured species whose genome sequences are available have been annotated as *Chlorobi* bacterium (OLB4, OLB5, OLB6, OLB7, and NICIL-2) branched outside of the main *Chlorobia* clade. Instead of grouping with the *Chlorobiales*, these sequences showed a closer relationship to the two *Ignavibacteria* species. Therefore, it is important to employ other means to reliably demarcate the family *Chlorobia* and its two family-level clades. With this objective, we have performed detailed comparative studies on protein sequences from *Chlorobia* genomes to identify molecular markers consisting of CSIs that are uniquely shared by members from the main clades of *Chlorobia* species. As noted in the introduction, CSIs in gene/protein sequences that are specifically shared by members from a given clade provide an important class of molecular markers for evolutionary and taxonomic studies [21,35,37,41,62,63]. Our analyses of protein sequences from *Chlorobia/Ignavibacteria* genomes have identified 50 new CSIs that are specific for the different main clades of these bacteria, providing important means for reliably demarcating these clades in molecular terms. The group-specificities and some characteristics of the identified CSIs are described below. Of the identified CSIs, 33 CSIs present in diverse proteins are commonly shared by all or most *Chlorobia* species for whom genome sequences are available. One example of a CSI specific for the class *Chlorobia*, as demarcated based on phylogenetic studies (Figure 1 and Figure 2), is presented in Figure 4.

Sequence information for the 32 other CSIs that are also specific for the class *Chlorobi* (order *Chlorobiales*) is presented in Figures S4–S35 and some of their characteristics are summarized in Table 1.

**Figure 5:**
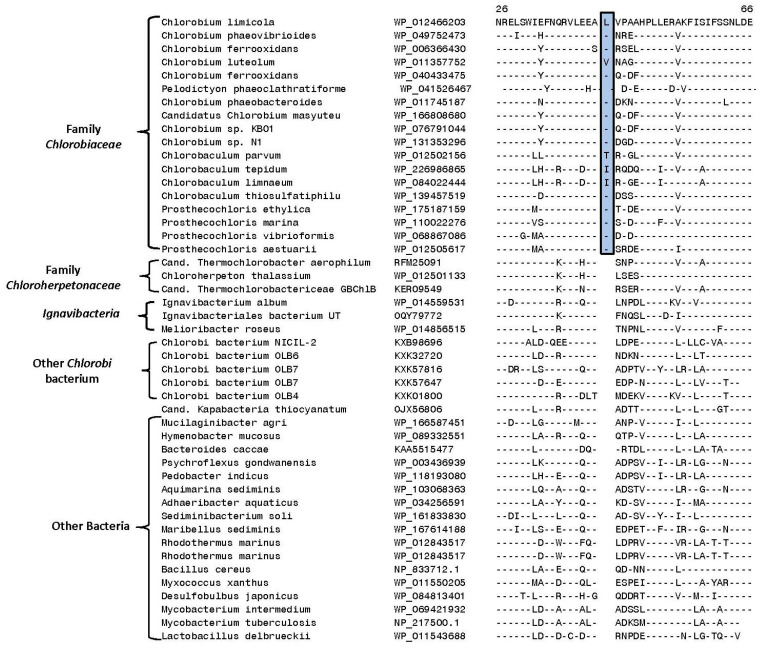
Excerpts from the sequence alignment of the protein polyphosphate kinase highlighting a one-amino-acid insertion (boxed) in a conserved region that is specifically shared by species/strains that are part of the family *Chlorobiaceae* (Figure 1). This insert is not found in members of the family *Chloroherpetonaceae*, class *Ignavibacteria*, other outgroup bacteria, as well as in other *Candidatus* and *Chlorobi* bacteria strains that do not group with the *Chlorobia* clade.

Our analyses have also identified multiple CSIs that are specific for the two family-level clades within the order *Chlorobiales*. Figure 5 presents a partial sequence alignment of the protein polyphosphate kinase-1 highlighting a one-amino-acid insert in a conserved region (boxed) that is exclusively shared by the members of the family *Chlorobiaceae*.

The polyphosphate kinase CSI in Figure 5 is commonly shared by all species/strains that are a part of the *Chlorobiaceae* family (see Figure 1), but it is absent in members of the family *Chloroherpetonaceae* as well as all other deeper branching species/strains including the *Ignavibacteria* species, unnamed *Chlorobi* bacteria, as well as different outgroup bacteria. In addition to this CSI, seven other CSIs identified in this study within other protein sequences are also specific for members of the family *Chlorobiaceae.* Sequence information for these other CSIs is presented in Figures S36–S42 and some of their characteristics are summarized in Table 2. Based on these CSIs, members of the family *Chlorobiaceae* can be clearly distinguished in molecular terms from all other bacteria.

**Figure 6:**
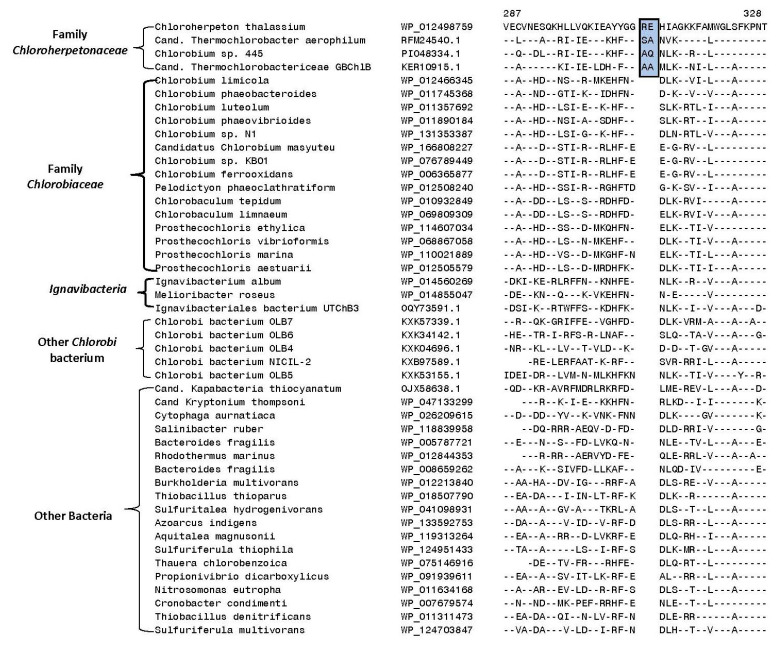
Partial sequence alignment of the protein UDP-glucose/GDP-mannose dehydrogenase showing a two-amino-acid insertion (boxed) in a conserved region that is exclusively shared by all species/strains that are part of the proposed family *Chloroherpetonaceae* (Figure 1). This insert is not found in members of the family *Chlorobiaceae*, class *Ignavibacteria*, as well as other *Candidatus* and *Chlorobi* bacteria strains that do not group with the *Chlorobia* clade. Sequence information for five other CSIs specific for the family *Chloroherpetonaceae* is presented in Figures S43–S47 and Table 2.

Six other CSIs identified in this work are specific for members of the family *Chloroherpetonaceae*. In Figure 6, we show a partial sequence alignment of the protein UDP-glucose GDP-mannose dehydrogenase, where a two-amino-acid insert is present in a conserved region (boxed) that is exclusively found in members of the family Chloroherpetonaceae but not found in any other *Chlorobia*/*Ignavibacteria* species or other bacteria. Sequence information for five other CSIs that are also specific for members of the family *Chloroherpetonaceae* is presented in Figures S43–S47 and some of their characteristics are summarized in Table 2. These CSIs provide strong and independent evidence for the distinctness of the family *Chloroherpetonaceae* from other *Chlorobiales* species and provide reliable means for the identification/demarcation of this clade.

**Figure 7:**
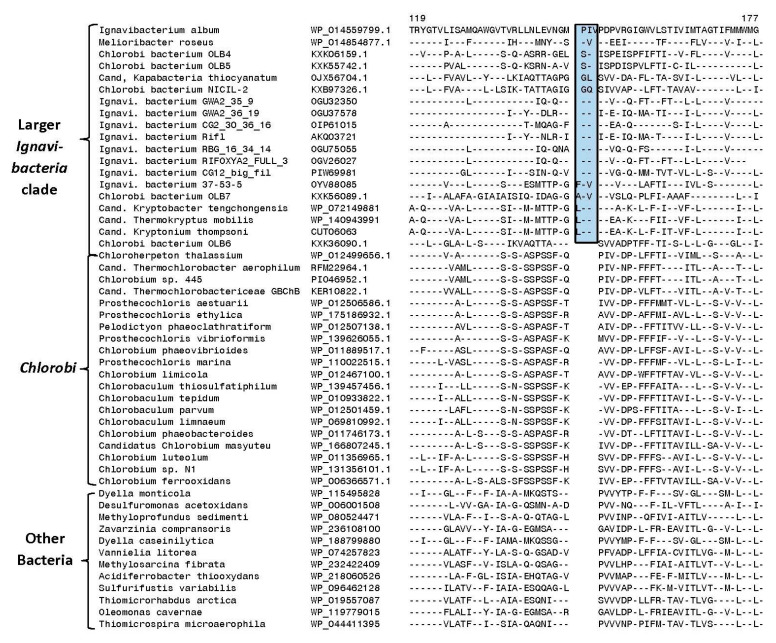
Partial sequence alignment of the protein preprotein translocase subunit SecY showing a two-to-three amino acid insertion (boxed) in a conserved region that is specifically shared by most of the species/strains that are a part of the larger *Ignavibacteria* clade (Figure 1). This insert is not found in members of the class *Chlorobia* or different outgroup bacteria examined, indicating that the genetic change giving rise to this CSI likely occurred in a common ancestor of the *Ignavibacteria* clade of species/strains.

Our analysis has also identified one CSI that is commonly shared by most of the species/strains that are a part of the larger *Ignavibacteria* clade. Sequence information for this CSI consisting of a two-to-three amino acid insert in the protein preprotein translocase subunit SecY is shown in Figure 7. This CSI, in addition to the known *Ignavibacteria* species/strains, is also commonly shared by specific *Candidatus* species and unnamed *Chlorobi* bacterium strains that group with the *Ignavibacteria* clade in our core protein tree. However, this CSI is absent in all *Chlorobiales* species and the outgroup bacteria examined.

**Figure 8:**
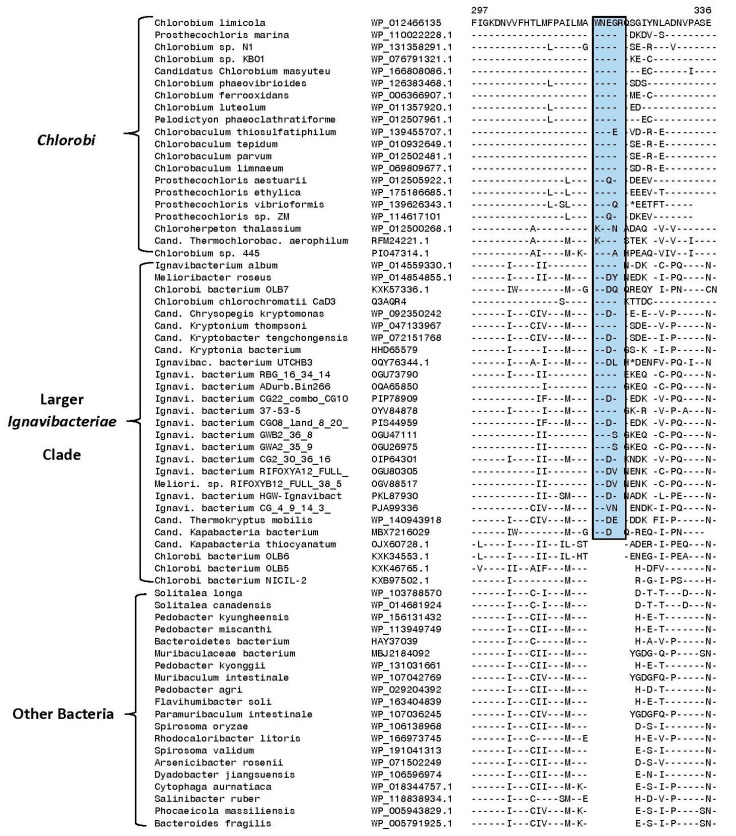
Partial sequence alignment of the protein methionine t-RNA ligase showing a four-amino-acid insertion (boxed) in a conserved region that is specifically shared by all Chlorobia species and most of the species/strains from the larger Ignavibacteria clade (Figure 1). However, this insert is not found in any other outgroup bacteria within the top 500 BLASTp hits. Sequence information for one additional CSI in the protein tRNA-dihydrouridine synthase that is also commonly shared by the species/strains from these two classes is presented in Figure S48.

Lastly, in phylogenetic trees, members of the class *Ignavibacteria* branch in the proximity of *Chlorobia* species (Figure 1) [25,26,27]. Our work has also identified two CSIs that are uniquely shared by all *Chlorobia* species and different *Ignavibacteria* species/strains. Figure 8 shows a partial sequence alignment of the protein methionine t-RNA ligase, where a four-amino-acid insert is present in a conserved region that is commonly shared by most species/strains from these two groups, but it is not present in different Bacteroidetes species examined or other closely related bacterial phyla in the top 500 BLASTp hits.

The CSI shown in Figure 8 in addition to different Chlorobia and Ignavibacteria species is also shared by several Candidatus species, which group within the larger Ignavibacteria clade in our phylogenomic tree (Figure 1). However, several Chlorobi bacterium (viz., OLB5, OLB6, and NICIL-2), which also group within the larger Ignavibacteria clade (Figure 1), do not share this CSI, indicating that the grouping together of all these species/strains is not confirmed by the identified CSI. Sequence information for another CSI, consisting of a one-amino-acid deletion in the protein tRNA-dihydrouridine synthase that is commonly shared by various Chlorobia and Ignavibacteria species/strains, is presented in Figure S48. This CSI is specific for the Chlorobia and Ignavibacteria species/strains, as homologs of this protein were not detected in other Candidatus species or Chlorobi bacterium strains.

## 4. Discussion

Members of the class *Chlorobia* constitute one of the seven discontinuous lineages of prokaryotic organisms that can carry out bacteriochlorophyll and chlorophyll-based photosynthesis [42,64,65]. As these bacteria are primarily found in a narrow environmental niche consisting of anoxic aquatic settings in stratified lakes, where sunlight can penetrate, identification and culturing of these bacteria are often not easy [3,4,5]. Thus, it is important to better understand the evolutionary relationships amongst these and related bacteria in addition to developing reliable means for their identification. Based on earlier work, members of the class *Ignavibacteria* are thought to be the closest relatives of *Chlorobia* [23,24,25,26,27,66]. Genome sequences are now available for most of the named *Chlorobia* and *Ignavibacteria* species as well as several other related uncultured species. In the present work, we have used these genome sequences to elucidate the evolutionary relationships among these organisms through several genome-sequence-based approaches. The approaches used to examine their evolutionary relationships include: (i) construction of a phylogenetic tree based on concatenated sequences of 282 core proteins from their genomes (Figure 1); (ii) construction of phylogenetic trees based on concatenated sequences of PolA–UvrD proteins (Figure S1), GyrA–GyrB proteins (Figure S2), and 16S rRNA gene sequences (Figure 2); (iii) determination of pairwise comparison matrices for *Chlorobia* species based on POCP (Figure 3) and AAI (Figure S3); and (iv) detailed analyses of protein sequences from *Chlorobia/Ignavibacteria* species, which have identified 50 novel conserved CSIs that are specific for different clades of these organisms. The CSIs in protein sequences result from rare genetic changes [21,41,56]. Hence, the shared presence of these molecular synapomorphies by a given group of species provides strong evidence that the species from that clade shared a common ancestor exclusive of other organisms and thus are specifically related to each other [21,41,56]. Additionally, earlier work on CSIs provides evidence that these molecular markers possess a high degree of predictive ability to be found in other unidentified or uncharacterized members of these clades [35,37,63].

Based on the results presented here, several consistent inferences can be drawn concerning the evolutionary relationships among *Chlorobia/Ignavibacteria* species. First, the results presented here confirm that the named *Chlorobia* species form a strongly supported clade, which can be reliably distinguished from *Ignavibacteria* and other bacteria by phylogenomic analysis and by 33 identified CSIs in diverse proteins that are uniquely shared (synapomorphies) by the members of this clade. The results from phylogenetic studies and clade specificities of the identified CSIs also reveal that several uncultured organisms that are referred to in the NCBI database as *Chlorobi* bacterium (OLB4, OLB5, OLB6, OLB7, and NICIL-2) are not related to the class *Chlorobia* and thus they are misclassified (or incorrectly annotated) as *Chlorobi* bacterium. Based on the grouping of these uncultured strains with the larger *Ignavibacteria* clade, rather than with the clade for *Chlorobia* species, it is suggested that in future these sequences should be referred to as the *Ignavibacteria* bacterium OLB4, OLB5, OLB6, OLB7, and NICIL-2. Second, the results presented here provide compelling evidence that the class *Chlorobia* or the order *Chlorobiales* comprises two distinct clades. The first of these clades grouping together different species from the genera *Chlorobaculum, Chlorobium, Pelodictyon,* and *Prosthecochloris* corresponds to the family *Chlorobiaceae*. The second clade harbors the species *Chloroherpeton thalassium, Candidatus* Thermochlorobacter aerophilum, *Candidatus* Thermochlorobacteriaceae bacterium GBChlB, and an unnamed *Chlorobium* sp. 445. Separation of the members of the order *Chlorobiales* into two family-level clades is also observed in other phylogenetic studies including the GTDB taxonomy [4,25,32]. Furthermore, the species from these two clades can also be distinguished from each other in pairwise matrices based on whole-genome comparison of POCP and AAI. However, the strongest evidence that the species from these two clades are distinct from each other is provided by our identification of eight and six CSIs in diverse proteins, respectively, that are exclusively shared by the members of these two clades. These CSIs provide a novel and unambiguous means for the identification and demarcation of the members of these two clades in molecular terms. Based on the clear distinction seen between the members of these two clades, based on phylogenomic and molecular sequence-based characteristics, we propose that the species from the second clade consisting of *Chloroherpeton*-related organisms be referred to as a new family *Chloroherpetonaceae* fam. nov.

**Figure 9:**
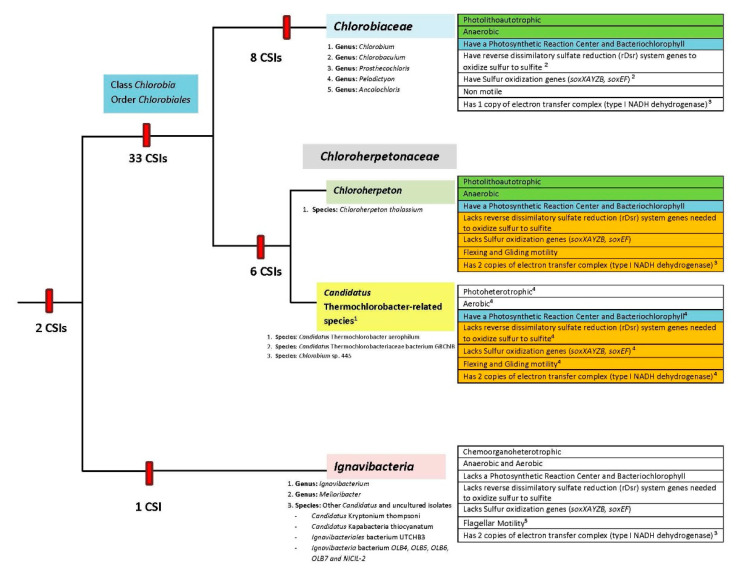
A conceptual diagram summarizing the evolutionary relationships among members of the phylum *Chlorobi* based on phylogenetic analysis and specific identified molecular signatures (synapomorphies). The numbers of CSIs that are specific for each clade or species-grouping are noted on the respective nodes. The species/genera present in each clade are listed underneath each label. The physiological and biochemical traits unique to each clade are listed on the right-hand side of the figure. Other notes: ^1^ These species have yet to be isolated and grown in pure culture; ^2^ *Chlorobium ferroxidans* is the sole exception; ^3^ Subunits NuoEFG missing from the sole copy in all *Chlorobiaceae* (11 subunits); *Chloroherpeton thalassium* NuoEFG missing from only one copy (14 and 11 subunits); *Candidatus* Thermochlorobacter aerophilum (12 and 11 subunits); *Ignavibacterium* album (14 and 11 subunits); ^4^ Inferred from genome sequences of *Chlorobium* sp. 445, *Candidatus* Thermochlorobacter aerophilum, and *Candidatus* Thermochlorobacteriaceae bacterium GBChlB; ^5^ Observations by light and electron microscopy suggested that *I. album* was not motile and lacked flagella (Iino et al., 2010 [7]). However, the *I. album* genome contains a nearly complete set of genes for flagella along with genes for chemotaxis and signal transduction. *Melioribacter roseus* is motile only during exponential growth, using flagella to move through water, but loses its flagella and mobility during stationary growth.

It should be noted that the species from the two main clades of *Chlorobiales* exhibit interesting clade-specific differences in growth, biochemical, and physiological characteristics [4,5,25,26,27,67,68]. Some of these differences are noted in Figure 9, which also summarizes the results from this study.

As noted in Figure 9, members of the family *Chlorobiaceae* are uniformly anaerobic, nonmotile, and photolithoautotrophic bacteria that use reduced sulfur compounds as reductants and electron source to fix CO_2_. They contain reverse dissimilatory sulfate reduction (rDsr) system genes to oxidize sulfur to sulfite as well as sulfur oxidization genes (soxXAYZB, soxEF) [25,67,68]. In contrast, members of the proposed *Chloroherpetonaceae* family exhibit important differences in their biochemical and physiological characteristics. Among these, *Chloroherpeton thalassium* is an anaerobic photoautolithotroph like the members of the family *Chlorobiaceae*. However, this species differs from the other *Chlorobiaceae* species in that it lacks the genes for the reverse dissimilatory sulfate reduction (rDsr) system as well as the sulfur oxidization genes (soxXAYZB, soxEF) needed to oxidize sulfur to sulfite. It also exhibits flexing and gliding motility. All other members of this clade are uncultured species that, based on their genome sequences, have been inferred to be aerobic photoheterotrophs, containing genes for photosynthetic reaction centers and bacteriochlorophyll biosynthesis, that likely use photo-assimilated acetate and propionate as carbon sources [25,26,27]. Based on the biochemical and physiological characteristics of *Chloroherpeton*, which overlap with those of *Chlorobiaceae*, it has been suggested [25,27] that this species/genus should be placed into a separate family distinct from the other Cand. *Thermochlorobacter*-related photoheterotrophs. However, based on considerations discussed in this work, we propose to keep all members of this clade in one family rather than two separate families. The main reasons for doing this are as follows. In the different phylogenetic trees constructed in this work and in earlier studies [25,32], *Chloroherpeton* groups reliably with the Cand. *Thermochlorobacter*-related photoheterotrophs. Six CSIs identified in this work are also uniquely shared by these two groups, providing strong evidence that the species from these two groups are specifically related. On the other hand, no CSI was identified that was commonly shared by only the *Chloroherpeton* and *Chlorobiaceae*. The GTDB taxonomy, which now provides a widely used reference resource for prokaryotic taxonomy, also places these species into a single family. If we place Cand. *Thermochlorobacter*-related photoheterotrophs into a separate family, it will not be possible to publish it as a valid family in the List of Prokaryotic Names with Standing in Nomenclature [11], as it does not contain any cultured species. However, in future work, if additional information becomes available for this clade of species, including an isolated cultured species for the Cand. *Thermochlorobacter*-related photoheterotrophs, the possibility of dividing the proposed family *Chloroherpetonaceae* into two families could be re-evaluated.

In contrast to the monophyly and reliable demarcation of the clades corresponding to the order *Chlorobiales* (class *Chlorobia*) and the two families within this order, our results indicate that the class *Ignavibacteria* is genetically highly diverse, and it is difficult at present to unambiguously delineate this taxon either in phylogenetic terms or by means of identified molecular markers. The class *Ignavibacteria* presently contains only two named species (*M. roseus* and *I. album*), which have been placed into two separate families within the order *Ignavibacteriales* [7,23,24]. However, unlike the class *Chlorobia*, where very few new species have been identified in the past 10–15 years, in the same period several *Candidatus* and other uncultured species/strains have been described that branch in the proximity of two *Ignavibacteria* species (Figure 1 and Figure 2) [17,27,59,60]. In the phylogenomic tree constructed in this work, the two *Ignavibacteria* species are part of a larger clade that includes several *Candidatus* species and uncultured *Chlorobi* species/strains. The GTDB taxonomy also indicates that these *Candidatus* and uncultured species/strains branch in the proximity of *Ignavibacteria* [32]. Although branching in a phylogenetic tree is affected by large numbers of variables, and can sometimes be misleading [69,70,71], a specific grouping or relationship of the species/strains that are a part of the larger *Ignavibacteria* clade is also independently supported by our identification of a CSI in the SecY protein that is uniquely shared by most of the species/strains from this clade (Figure 7). Two additional CSIs identified in this work, which are commonly shared by different *Chlorobia* species and several species/strains from this larger *Ignavibacteria* clade, provide further support that these specific *Candidatus* and uncultured species/strains are related to these bacteria. These observations suggest that the circumscription of the class *Ignavibacteria* should be expanded to include these other *Candidatus* and uncultured *Chlorobi* species/strains (i.e., corresponding to the large *Ignavibacteria* clade (Figure 1)). However, we refrain from making a formal proposal, because the number of uncultured *Candidatus* species/strains related to this group is rapidly expanding in the public databases. Thus, more detailed future studies on *Ignavibacteria* and related species/strains need be conducted to develop a more precise and informative classification scheme for this group/class of bacteria, which will likely lead to its division into several different order/family-level taxa.

The present study has identified many molecular markers (CSIs) that are exclusively found in members of different clades within the *Chlorobia/Ignavibacteria* groups of bacteria. Extensive earlier work on CSIs specific for other prokaryotic taxa provides compelling evidence that these molecular markers possess a high degree of predictive ability to be found in other members from these clades whose genome sequences are currently unavailable, as well in novel and uncultured species that are related to these specific clades [39,57,62,63,72,73]. Due to the presence of these CSIs in conserved regions of different genes/proteins, they provide a novel means for the identification of other species from these taxa by either in silico analysis of genomic sequences (based on BLASTp searches checking for the presence or absence of these molecular sequences) or experimental means using PCR-based assays [62,74,75,76]. Most of the novel species/strains related to the *Chlorobia/Ignavibacteria* taxa reported in recent years consist of uncultured species that have been identified based on analyses of metagenomic sequences from diverse geological habitats [17,27,59,60]. In this context, the molecular markers described here, which are highly specific for these groups of bacteria, should prove helpful in the identification and characterization of other novel species/strains related to these taxa. Lastly, earlier work on CSIs provides evidence that these molecular characteristics are functionally important for the group of organisms for which they are specific, and several of these CSIs found in key chlorophyll–bacteriochlorophyll biosynthesis proteins and core centre proteins have provided important insights into the evolutionary relationships among different groups/phyla of photosynthetic bacteria [77,78,79,80]. Currently, very few reliable characteristics are known that are specific for the *Chlorobia/Chlorobiales* species [3,4]. Therefore, to incorporate the information for the CSIs that are specific for the order *Chlorobiales* and the family *Chlorobiaceae*, we also provide emended descriptions of these taxa. In addition, we provide a formal description of the class *Chlorobia* as this has not yet been validly published [11]. The descriptions of the emended and novel taxa are given below.

**Description of the Class *Chlorobia* class nov. (Garrity and Holt 2001, 601^EP^)**

*Chlorobia* (Chlo.ro’bi.a. N.L. neut. n. *Chlorobium*, genus of the class, changing ending to denote a class; N.L. neut. pl. n. *Chlorobia*, class of the genus *Chlorobium*).

The class *Chlorobia* contains a single order (*Chlorobiales*) [8] and the description of this class is the same as that given below for the order *Chlorobiales.*

The type order is *Chlorobiales* Gibbons and Murray 1978 (Approved Lists 1980).

**Emended Description of the order *Chlorobiales* Gibbons and Murray 1978 (Approved Lists 1980)**

The order is composed of two families: *Chlorobiaceae* and *Chloroherpetonaceae.* Members of this order form a monophyletic clade in phylogenetic trees based on 16S rRNA gene sequences and trees based on several individual proteins (viz., Fmo, GyrA, GyrB, PolA, RecA, and UvrD) and large datasets of concatenated protein sequences. The order comprises species that are anaerobic and strict photolithoautotrophs, which use reduced sulfur compounds as an electron source to fix CO_2_, as well as aerobic photoautotrophic organisms, which likely use photo-assimilated acetate and propionate as carbon sources. Species from this phylum generally contain genes encoding for photosynthetic reaction centers and bacteriochlorophyll biosynthesis. The members of this order can be reliably distinguished from all other bacteria based on the shared presence of 33 conserved signature indels (CSIs) in diverse proteins (Table 1) that are uniquely shared by the members of this order. The CSIs specific for the order *Chlorobiales* are found in the following proteins: a major facilitator superfamily protein, a radical SAM-domain-containing protein, a Gfo/Idh/MocA family oxidoreductase, an acetyl-CoA carboxylase carboxyltransferase alpha subunit, a DNA mismatch repair protein, bifunctional 5,10-methylene-tetrahydrofolate dehydrogenase, cytidylate kinase, deoxyribonucleotide triphosphate pyrophosphatase, a DNA polymerase III alpha subunit, DNA polymerase III subunits gamma and tau, fructose-1,6-bisphosphate aldolase (class II), glutamate-1-semialdehyde aminotransferase, glutamyl-tRNA reductase, glutamate-1-semialdehyde aminotransferase, glycyl-tRNA synthetase, 4-hydroxy-3-methylbut-2-enyl diphosphate reductase, lactoylglutathione lyase, molecular chaperone DnaK, phosphoribosylformylglycinamidine synthase II, polynucleotide phosphorylase/polyadenylase, ribonuclease R, an RNA polymerase sigma-32 subunit, serine hydroxymethyltransferase, succinyl-CoA synthetase subunit alpha, thiazole synthase, tryptophanyl-tRNA synthetase, uroporphyrinogen decarboxylase, N-acetyl-alpha-D-glucosaminyl L-malate synthase BshA, and 7-carboxy-7-deazaguanine synthase QueE.

The type genus of this order is *Chlorobium* Nadson 1906 [81] (Approved Lists 1980).

**Emended Description of the family *Chlorobiaceae* Copeland 1956 (Approved Lists 1980)**

The family *Chlorobiaceae* contains the type genus *Chlorobium* [8,81] and the following validly published genera: *Ancalochloris* [8,14], *Chlorobaculum* [12], *Pelodictyon* Lauterborn [8], and *Prosthecochloris* [8,14]. The description of this family is partially based on that given by Imhoff [4]. Members of this family are non-motile photolithoautotrophs and they grow under anoxic conditions in the presence of limited light using reduced sulfur compounds as an electron source to fix CO_2_. Thiosulfate, hydrogen, and ferrous iron can also be used as reductants. Most of the members of this family have been isolated from anoxic aquatic settings in stratified lakes, where sunlight can penetrate. All members of this family except *Chlorobium ferroxidans* possess a dissimilatory sulfite reductase (DSR) system. Members of this family also form a monophyletic clade in phylogenetic trees based on 16S rRNA gene sequences and trees constructed based on large datasets of concatenated protein sequences. The members of this order can be clearly distinguished from members of the family *Chloroherpetonaceae* and all other bacteria based on the shared presence of eight conserved signature indels (CSIs) found in diverse proteins (listed in Table 2) that are uniquely shared by the members of this family. The proteins containing the CSIs specific for this family are as follows: biogenesis of lysosome-related organelles complex 1 subunit 2, DegT/DnrJ/EryC1/StrS aminotransferase, DNA gyrase subunit A, molecular chaperone HtpG, MiaB-like tRNA modifying protein, peptidase U32, peptide chain release factor 3, and polyphosphate kinase.

The type genus of this family is *Chlorobium* Nadson 1906 [81] (Approved Lists 1980).

**Description of the family *Chloroherpetonaceae* fam. nov.**

*Chloroherpetonaceae* (Chlo.ro.her’pe.to.na.ce’ae. N.L. neut. n. *Chloroherpeton*, type genus of the family; -aceae ending to denote a family; N.L. fem. pl. n. *Chloroherpetonaceae* the *Chloroherpeton* family).

This family contains the type genus *Chloroherpeton* and another *Candidatus* genus Thermochlorobacter. In addition, two uncultured organisms with assembled genomes known by the names *Chlorobium* sp. 445 and *Candidatus* Thermochlorobacteriaceae GBChlB are also members of this family. Like the *Chlorobiaceae* family, members of this family contain type-I reaction centers and chlorosomes. The sole cultured species from this family is *Chloroherpeton thalassium*, which like members of the *Chlorobiaceae* family is a photolithoautotroph that grows under anoxic conditions in the presence of light using reduced sulfur compounds as an electron source to fix CO_2_. However, unlike *Chlorobiaceae* species, which are nonmotile, cells of *Chloroherpeton thalassium* exhibit gliding mobility. In contrast to *Chloroherpeton*, the *Candidatus* species Thermochlorobacter aerophilum is an aerobic photoheterotroph that cannot oxidize sulfur compounds, cannot fix N_2_, and does not fix CO_2_ autotrophically [27]. It is considered to have gliding and flexing motility and two copies of the type-1 NADH dehydrogenase complex similarly to *Chloroherpeton thalassium*. Members of this family are characterized by their lack of a dissimilatory sulfite reductase (DSR) system, which is a protein present in all members of *Chlorobiaceae* except for *Chlorobium ferroxidans*. Members of this family form a monophyletic clade in a phylogenetic tree based on concatenated sequences of core proteins from the genomes of *Chlorobiales* species. The members of this family are also clearly differentiated from the *Chlorobiaceae* species in a pairwise matrix of the percentage of conserved proteins in the genomes. Furthermore, members of this family can be reliably distinguished from members of the family *Chlorobiaceae* and all other bacteria based on the shared presence of six conserved signature indels (CSIs) found in diverse proteins (Table 2) that are uniquely shared by members of this family. The proteins containing the CSIs specific for this family are: alkaline phosphatase family protein, dihydrolipoyl dehydrogenase, hypoxanthine phosphoribosyltransferase, SDR family oxidoreductase, RecQ family ATP-dependent DNA helicase, and UDP-glucose/GDP-mannose dehydrogenase family protein.

The type genus for this family is *Chloroherpeton* Gibson et al. 1985 [13,82].

## Figures and Tables

**Table 1:** Conserved signature indels specific for members of the order *Chlorobiales* *^,a^.

Protein Name	Accession/GI Number	Figure No.	Indel Size	Indel Position
Phosphoribosylformylglycinamidine synthase II	WP_041465690	Figure 4	5 aa ins	270–320
Glycyl-tRNA synthetase	78185909	Figure S4	1 aa ins	248–314
RNA polymerase sigma-32 subunit	194337394	Figure S5	12 aa ins	24–79
Uroporphyrinogen decarboxylase	189347814	Figure S6	3 aa ins	284–333
Glutamyl-tRNA reductase	194336061	Figure S7	1 aa ins	211–250
Glutamate-1-semialdehyde aminotransferase	21674908	Figure S8	1 aa ins	60–109
fructose-1,6-bisphosphate aldolase, class II	189500266	Figure S9	11 aa del	233–282
A major facilitator superfamily protein	193212482	Figure S10	1 aa ins	325–365
DNA polymerase III subunit alpha	78187124	Figure S11	2–4 aa ins	294–340
Acetyl-CoA carboxylase carboxyltransferase subunit alpha	119356086	Figure S12	6 aa ins	103–137
N-acetyl-alpha-D-glucosaminyl L-malate synthase BshA	193214252	Figure S13	3 aa ins	88–132
Serine hydroxymethyltransferase	193215659	Figure S14	2 aa ins	228–280
Deoxyribonucleotide triphosphate pyrophosphatase	193215546	Figure S15	3 aa ins	94–144
Polynucleotide phosphorylase/polyadenylase	189346136	Figure S16	1 aa ins	37–69
Polynucleotide phosphorylase/polyadenylase	189346136	Figure S17	17 aa ins	264–335
7-carboxy-7-deazaguanine synthase QueE	500067752	Figure S18	3–7 aa ins	119–164
DNA polymerase III subunits gamma and tau	193213861	Figure S19	2 aa ins	47–93
DNA polymerase III subunits gamma and tau	193213861	Figure S20	4 aa ins	220–268
Cytidylate kinase	21673125	Figure S21	4 aa ins	146–199
4-hydroxy-3-methylbut-2-enyl diphosphate reductase	194333263	Figure S22	2–5 aa ins	264–311
molecular chaperone DnaK	193214979	Figure S23	1 aa del	70–119
Tryptophanyl-tRNA synthetase	78188056	Figure S24	8 aa ins	16–59
Tryptophanyl-tRNA synthase	493409794	Figure S25	5 aa ins	153–204
Succinyl-CoA synthetase subunit alpha	193216377	Figure S26	7 aa ins	31–87
A Gfo/Idh/MocA family oxidoreductase	21673819	Figure S27	4 aa ins	2–48
A Gfo/Idh/MocA family oxidoreductase	21673819	Figure S28	2 aa ins	209–249
Ribonuclease R	193213033	Figure S29	7–8 aa ins	176–223
Ribonuclease R	193213033	Figure S30	4–5 aa ins	558–608
Ribonuclease R	193213033	Figure S31	4 aa ins	640–720
Bifunctional 5,10-methylene-THF dehydrogenase	78186789	Figure S32	2 aa ins	128–161
Lactoylglutathione lyase	78187149	Figure S33	2 aa ins	5–46
Thiazole synthase	78187419	Figure S34	1 aa ins	55–106
DNA mismatch repair protein *	193213813	Figure S35	1 aa del	599–644

* Except for an isolated exception all of these CSIs are specific for the *Chlorobia*/*Chlorobiales* species. ^a^ For some CSIs, homologs were not found in all *Chlorobia* species.

**Table 2:** Conserved Signature Indels Specific for Members of the families *Chlorobiaceae* and *Chloroherpetonaceae* *^,a^.

Protein Name	Accession/GI Number	Figure No.	Indel Size	Indel Position	Specificity
Polyphosphate kinase	WP_012466203	Figure 5	1 aa ins	25–65	Family *Chlorobiaceae*
Peptide chain release factor 3	193212508	Figure S36	1 aa del	400–447	
Peptidase U32	493410285	Figure S37	6 aa ins	260–316	
MiaB-like tRNA modifying protein	145220314	Figure S38	5 aa ins	329–382	
Molecular chaperone HtpG	145219831	Figure S39	3 aa ins	259–314	
DegT/DnrJ/EryC1/StrS aminotransferase	193212751	Figure S40	1 aa del	111–156	
biogenesis of lysosome-related organelles complex 1 subunit 2	194336576	Figure S41	1 aa del	238–279	
DNA gyrase subunit A	194335275	Figure S42	1 aa ins	234–268	
UDP-glucose/GDP-mannose dehydrogenase family protein	WP_012498759	Figure 6	2 aa ins	287–330	Family *Chloroherpetonaceae*
hypoxanthine phosphoribosyltransferase	PIO48526	Figure S43	1 aa ins	57–94	
dihydrolipoyl dehydrogenase	PIO48610	Figure S44	1 aa ins	30–64	
SDR family oxidoreductase	WP_012499003	Figure S45	4 aa ins	236–280	
RecQ family ATP-dependent DNA helicase	WP_012499407	Figure S46	2 aa ins	164- 203	
alkaline phosphatase family protein	RFM24133	Figure S47	5 aa ins	33–78	

* Except for an isolated exception, all of these CSIs are specific for the members of the indicated families. ^a^ For some CSIs, homologs were not identified in all members of a given family.

## Data Availability

The data presented in this study are available in a publicly accessible repository (https://www.ncbi.nlm.nih.gov/genome/, accessed on 10 January 2022) and the Supplementary Material.

## Supplementary Materials

The following supporting information can be downloaded at: https://www.mdpi.com/article/10.3390/microorganisms10071312/s1, Figure S1. A bootstrapped maximum likelihood tree for different genome-sequenced *Chlorobia*/*Ignavibacteria* and related species/strains based on concatenated sequences of UvrD and PolA proteins. Figure S2. A bootstrapped maximum-likelihood tree for different genome-sequenced *Chlorobia*/*Ignavibacteria* and related species/strains based on concatenated sequences of GyrA and GyrB proteins. Figure S3. AAI matrix indicating the pairwise percentage average amino acid identities based on core proteins for the type species of different genera within the order *Chlorobiales*. Figure S4. Partial sequence alignment of the protein glycine-tRNA synthetase, showing a 1 aa insertion (boxed) that is specific for the order *Chlorobiales*. Figure S5. Partial sequence alignment of the protein RNA polymerase sigma factor RpoD/SigA, showing a 12 aa insertion (boxed) that is exclusive to all members belonging to the order *Chlorobiales*. Figure S6 Partial sequence alignment of the protein uroporphyrinogen decarboxylase, showing a 3 aa insertion (boxed) that is exclusive to all members belonging to the order *Chlorobiales*. Figure S7. Partial sequence alignment of the protein glutamyl-tRNA reductase, showing a 1 aa insertion (boxed) that is exclusive to all members belonging to the order *Chlorobiales*. Figure S8. Partial sequence alignment of the protein glutamate-1-semialdehyde 2,1-aminomutase, showing a 1 aa insertion (boxed) that is found in all members belonging to the order *Chlorobiales.* Figure S9. Partial sequence alignment of the protein class II fructose-1,6-bisphosphate aldolase, showing an 11 aa deletion (boxed) that is found in all members belonging to the order *Chlorobiales*. Figure S10. Partial sequence alignment of the protein major facilitator superfamily transporter, showing a 1 aa insertion (boxed) that is specific for members of the order *Chlorobiales*. Figure S11. Partial sequence alignment of the protein DNA polymerase III subunit alpha, showing a 2–4 aa insert (boxed) that is specific for all members belonging to the order *Chlorobiales*. Figure S12. Partial sequence alignment of the protein acetyl-CoA carboxylase carboxyltransferase subunit alpha, showing a 6 aa insertion (boxed) that is found in all members belonging to the order *Chlorobiales*. Figure S13. Partial sequence alignment of the protein N-acetyl-alpha-D-glucosaminyl L-malate synthase BshA, showing a 3 aa insertion (boxed) that is found in all members belonging to the order *Chlorobiales*. Figure S14. Partial sequence alignment of the protein serine hydroxymethyltransferase, showing a 2 aa insertion (boxed) that is found in all members belonging to the order *Chlorobiales*. Figure S15. Partial sequence alignment of the protein deoxyribonucleotide triphosphate pyrophosphatase, showing a 3 aa insertion (boxed) that is found in all members belonging to the order *Chlorobiales*. Figure S16. Partial sequence alignment of the protein polynucleotide phosphorylase/polyadenylase, showing a 1 aa insertion (boxed) that is found in all members belonging to the order *Chlorobiales*. Figure S17. Partial sequence alignment of the protein polynucleotide phosphorylase/polyadenylase, showing a 17 aa insertion (boxed) that is found in all members belonging to the order *Chlorobiales*. Figure S18. Partial sequence alignment of the protein 7-carboxy-7-deazaguanine synthase QueE, showing a 3–7 aa insertion (boxed) that is found in all members belonging to the order *Chlorobiales*. While other *Chlorobia* species contain a 7 aa insertion, *Chlorobaculum* species have a 3 aa insertion in this position. Figure S19. Partial sequence alignment of the protein DNA polymerase III subunits gamma and tau, showing a 2 aa insertion (boxed) that is found in all members belonging to the order *Chlorobiales*. Figure S20. Partial sequence alignment of the protein DNA polymerase III subunits gamma and tau, showing a 4 aa insertion (boxed) that is found in all members belonging to the order *Chlorobiales.* Figure S21. Partial sequence alignment of the protein cytidylate kinase, showing a 4 aa insertion (boxed) that is found in all members belonging to the order *Chlorobiales*. Figure S22. Partial sequence alignment of the protein 4-hydroxy-3-methylbut-2-enyl diphosphate reductase, showing a 2–5 aa insertion (boxed) that is found in all members belonging to the order *Chlorobiales*. Figure S23. Partial sequence alignment of the protein molecular chaperone DnaK, showing a 1 aa deletion (boxed) that is found in all members belonging to the order *Chlorobiales*. Figure S24. Partial sequence alignment of the protein tryptophanyl-tRNA synthetase, showing an 8 aa insert (boxed) that is found in all members belonging to the order *Chlorobiales*. Figure S25. Partial sequence alignment of the protein tryptophanyl-tRNA synthetase, showing a 5 aa insert (boxed) that is found in all members belonging to the order *Chlorobiales*. Figure S26. Partial sequence alignment of the protein succinyl-CoA synthetase subunit alpha, showing a 7 aa insert (boxed) that is found in all members belonging to the order *Chlorobiales*. Figure S27. Partial sequence alignment of the protein Gfo/Idh/MocA family oxidoreductase, showing a 4 aa insert (boxed) that is found in all members belonging to the order *Chlorobiales*. Figure S28. Partial sequence alignment of the protein Gfo/Idh/MocA family oxidoreductase, showing a 2 aa insert (boxed) that is found in all members belonging to the order *Chlorobiales*. Figure S29. Partial sequence alignment of the protein ribonuclease R, showing a 7–8 aa insert (boxed) that is found in all members belonging to the order *Chlorobiales*. Figure S30. Partial sequence alignment of the protein ribonuclease R, showing a 4–5 aa insert (boxed) that is found in all members belonging to the order *Chlorobiales.* Figure S31. Partial sequence alignment of the protein ribonuclease R, showing a 4 aa insert (boxed) that is found in all members belonging to the order *Chlorobiales*. Figure S32. Partial sequence alignment of the protein bifunctional 5,10-methylene-tetrahydrofolate dehydrogenase, showing a 2 aa insert (boxed) that is found in all members belonging to the order *Chlorobiales*. Figure S33. Partial sequence alignment of the protein lactoylglutathione lyase, showing a 2 aa insert (boxed) that is found in all members belonging to the order *Chlorobiales*. Figure S34. Partial sequence alignment of the protein thiazole synthase, showing a 1 aa insert (boxed) that is found in all members belonging to the order *Chlorobiales*. Figure S35. Partial sequence alignment of the protein DNA mismatch repair protein (MutS), showing a 1 aa deletion (boxed) that is found in all members belonging to the order *Chlorobiales*. Figure S36. Partial sequence alignment of the protein peptide chain release factor 3, showing a 1 aa deletion (boxed) that is specific for members of the family *Chlorobiaceae*. Figure S37. Partial sequence alignment of the protein U32 family peptidase, showing a 6 aa insertion (boxed) that is specific for members of the family *Chlorobiaceae*. Figure S38. Partial sequence alignment of the MiaB-like tRNA modifying protein, showing a 5 aa insertion (boxed) that is specific for members belonging to the family *Chlorobiaceae*. Figure S39. Partial sequence alignment of the molecular chaperone HtpG protein, showing a 3 aa insertion (boxed) that is specific for members of the family *Chlorobiaceae*. Figure S40. Partial sequence alignment of the DegT/DnrJ/EryC1/StrS family aminotransferase protein, showing a 1 aa deletion (boxed) that is specific for the family *Chlorobiaceae*. Figure S41. Partial sequence alignment of the biogenesis of lysosome-related organelles complex 1 subunit 2 protein, showing a 1 aa deletion (boxed) that is specific for members of the family *Chlorobiaceae*. Figure S42. Partial sequence alignment of the protein DNA gyrase subunit A, showing a 1 aa insertion (boxed) that is specific for members belonging to the family *Chlorobiaceae*. Figure S43. Partial sequence alignment of the protein hypoxanthine phosphoribosyltransferase, showing a 1 aa insertion (boxed) that is exclusively shared by all members of the family *Chloroherpetonaceae*. Figure S44. Partial sequence alignment of the protein dihydrolipoyl dehydrogenase containing a 1 aa insertion (boxed) that is specific for members of the family *Chloroherpetonaceae*. Figure S45. Partial sequence alignment of the protein SDR family oxidoreductase, showing a 4 aa insertion (boxed) that is specific for members of the family *Chloroherpetonaceae*. Figure S46. Partial sequence alignment of the protein RecQ family ATP-dependent DNA helicase, showing a 2 aa insertion (boxed) that is specific for members of the family *Chloroherpetonaceae*. Figure S47. Partial sequence alignment of the alkaline phosphatase family protein, showing a 5 aa insertion (boxed) that is specific for members of the family *Chloroherpetonaceae*. Figure S48. Partial sequence alignment of the protein tRNA dihydrouridine synthase DusB, showing a 1 aa insertion (boxed) that is specific for members of the order *Chlorobiales* and the “larger *Ignavibacteriae* clade”.
