# Supplementary material for: Phylogenomic Analyses and Molecular Signatures Elucidating the Evolutionary Relationships amongst the Chlorobia and Ignavibacteria Species: Robust Demarcation of Two Family-Level Clades within the Order Chlorobiales and Proposal for the Family Chloroherpetonaceae fam. nov
Source: Microorganisms. 2022 Jun 29;10(7):1312. doi: 10.3390/microorganisms10071312 (PMC9318685; doi:10.3390/microorganisms10071312)
Supplement: Supplementary file 1 [file microorganisms-10-01312-s001.zip › microorganisms-1695449-supplementary.pdf]

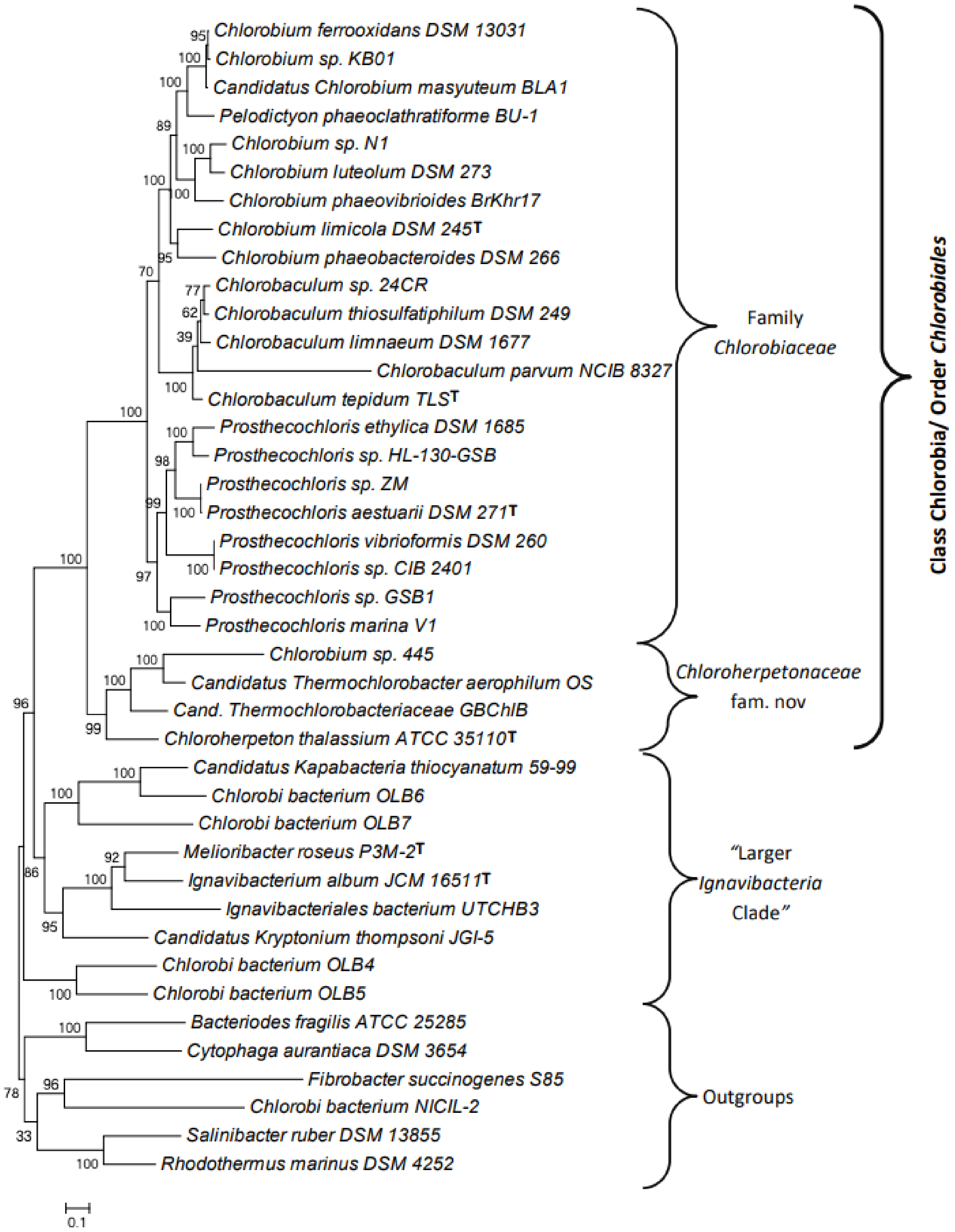

Figure S1

A bootstrapped maximum likelihood tree for different genomes-sequenced Chlorobia/Ignavibacteria and related species/strains based on concatenated sequences of UvrD and PolA proteins. Statistical support values for different branches are indicated on the nodes. The tree was rooted using the sequences from the species Fibrobacter succinogenes. The type strains of different species are marked by the superscript "T". Some species clades observed in this tree are marked.

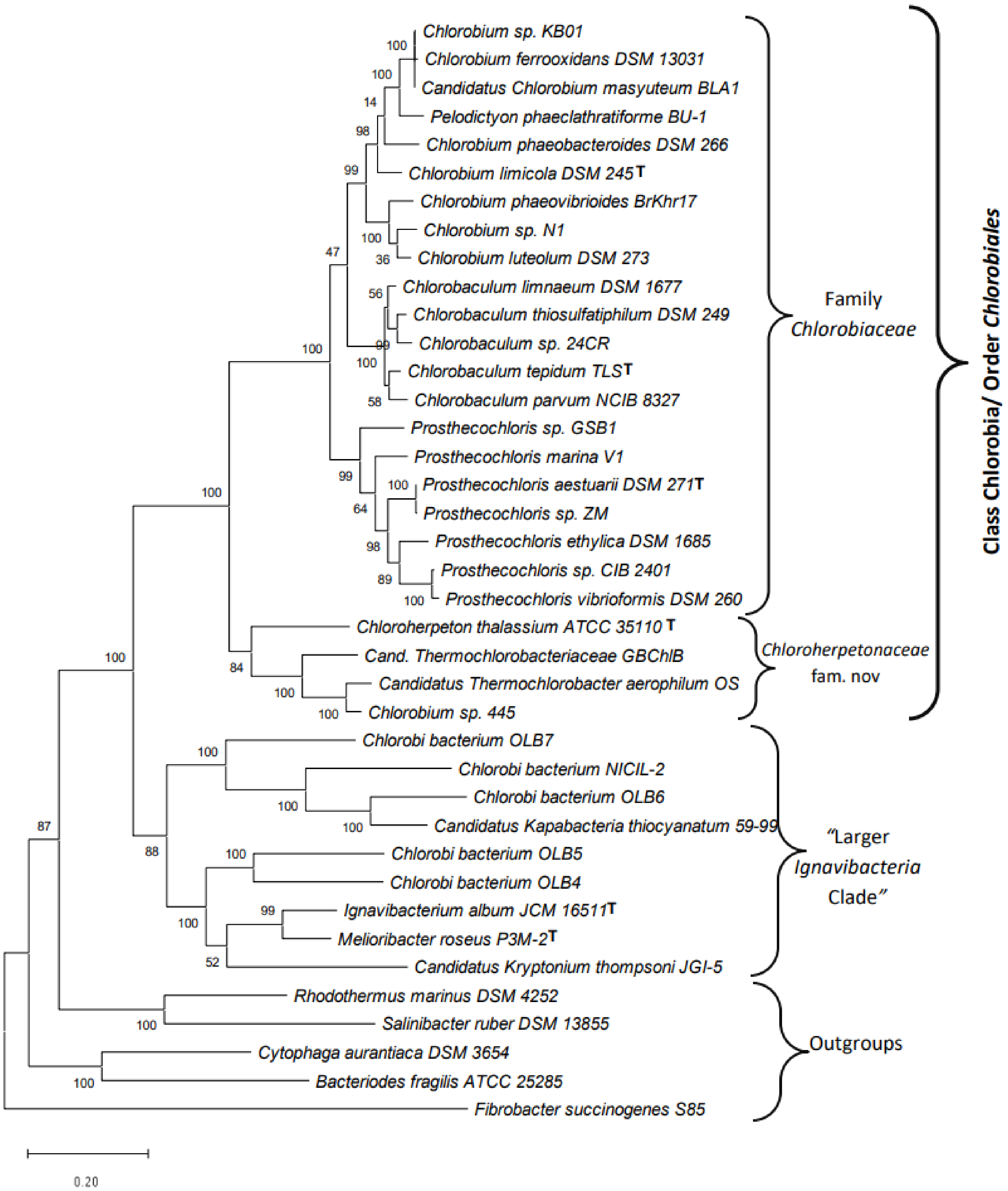

Figure S2

A bootstrapped maximum likelihood tree for different genomes-sequenced Chlorobia/Ignavibacteria and related species/strains based on concatenated sequences of GyrA and Gyr B proteins. Statistical support values for different branches are indicated on the nodes. The tree was rooted using the sequences from the species Fibrobacter succinogenes. The type strains of different species are marked by the superscript "T". Some species clades observed in this tree are marked.

Family	Species	*Chloroherpeton thalassium*	*Candidatus Thermochlorobacter aerophilum*	*Candidatus Thermochlorobacteriaceae GBChlB*	*Chlorobium* sp. 445	*Chlorobaculum tepidum*	*Chlorobaculum parvum*	*Chlorobaculum limnaeum*	*Chlorobaculum thiosulfatiphilum*	*Pelodictyon phaeoclathratiforme*	*Chlorobium phaeobacteroides*	*Chlorobium limicola*	*Chlorobium luteolum*	*Chlorobium ferrooxidans*	*Chlorobium phaeovibrioides*	*Prosthecochloris marina*	*Prosthecochloris aestuarii*	*Prosthecochloris vibrioformis*	*Prosthecochloris ethylica*	*Candidatus Chlorobium masyuteum*
Chloroherpetonaceae **fam.nov**	*Chloroherpeton thalassium*	1	0.69	0.7	0.69	0.69	0.7	0.7	0.69	0.69	0.69	0.69	0.69	0.69	0.69	0.7	0.69	0.69	0.69	0.69
	Candidatus Thermochlorobacter aerophilum	0.69	1	0.73	0.8	0.67	0.67	0.67	0.67	0.67	0.67	0.67	0.67	0.67	0.67	0.67	0.67	0.67	0.67	0.67
	Candidatus Thermochlorobacteriaceae GBChlB	0.7	0.73	1	0.73	0.67	0.67	0.67	0.67	0.67	0.67	0.67	0.67	0.67	0.67	0.67	0.67	0.67	0.67	0.67
	Chlorobium sp. 445	0.69	0.8	0.73	1	0.68	0.68	0.68	0.68	0.68	0.68	0.68	0.68	0.68	0.67	0.68	0.68	0.68	0.67	0.68
**Family *Chlorobiaceae***	*Chlorobaculum tepidum*	0.69	0.67	0.67	0.68	1	0.83	0.87	0.87	0.73	0.73	0.74	0.73	0.73	0.73	0.72	0.73	0.72	0.72	0.74
	Chlorobaculum parvum	0.7	0.67	0.67	0.68	0.83	1	0.82	0.82	0.73	0.73	0.74	0.73	0.73	0.73	0.72	0.73	0.73	0.73	0.73
	Chlorobaculum limnaeum	0.7	0.67	0.67	0.68	0.87	0.82	1	0.9	0.73	0.73	0.74	0.74	0.74	0.73	0.72	0.73	0.72	0.72	0.74
	Chlorobaculum thiosulfatiphilum	0.69	0.67	0.67	0.68	0.87	0.82	0.9	1	0.73	0.73	0.74	0.73	0.73	0.73	0.72	0.73	0.72	0.72	0.73
	Pelodictyon phaeoclathratiforme	0.69	0.67	0.67	0.68	0.73	0.73	0.73	0.73	1	0.77	0.76	0.75	0.8	0.75	0.72	0.73	0.72	0.72	0.8
	Chlorobium phaeobacteroides	0.69	0.67	0.67	0.68	0.73	0.73	0.73	0.73	0.77	1	0.78	0.74	0.76	0.74	0.72	0.73	0.72	0.72	0.76
	Chlorobium limicola	0.69	0.67	0.67	0.68	0.74	0.74	0.74	0.74	0.76	0.78	1	0.75	0.76	0.75	0.73	0.73	0.73	0.73	0.76
	Chlorobium luteolum	0.69	0.67	0.67	0.68	0.73	0.73	0.74	0.73	0.75	0.74	0.75	1	0.75	0.8	0.72	0.72	0.72	0.72	0.76
	Chlorobium ferrooxidans	0.69	0.67	0.67	0.68	0.73	0.73	0.74	0.73	0.8	0.76	0.76	0.75	1	0.76	0.72	0.73	0.72	0.72	0.95
	Chlorobium phaeovibrioides	0.69	0.67	0.67	0.67	0.73	0.73	0.73	0.73	0.75	0.74	0.75	0.8	0.76	1	0.72	0.72	0.72	0.72	0.76
	Prosthecochloris marina	0.7	0.67	0.67	0.68	0.72	0.72	0.72	0.72	0.72	0.72	0.73	0.72	0.72	0.72	1	0.75	0.75	0.75	0.72
	Prosthecochloris aestuarii	0.69	0.67	0.67	0.68	0.73	0.73	0.73	0.73	0.73	0.73	0.73	0.72	0.73	0.72	0.75	1	0.77	0.78	0.73
	Prosthecochloris vibrioformis	0.69	0.67	0.67	0.68	0.72	0.73	0.72	0.72	0.72	0.72	0.73	0.72	0.72	0.72	0.75	0.77	1	0.78	0.72
	Prosthecochloris ethylica	0.69	0.67	0.67	0.67	0.72	0.73	0.72	0.72	0.72	0.72	0.73	0.72	0.72	0.72	0.75	0.78	0.78	1	0.72
	Candidatus Chlorobium masyuteum	0.69	0.67	0.67	0.68	0.74	0.73	0.74	0.73	0.8	0.76	0.76	0.76	0.95	0.76	0.72	0.73	0.72	0.72	1

0.66 — 1.00

Figure S3

AAI matrix indicating the pairwise percentage average amino acid identities based on 355 core proteins for the type species of different genera within the order *Chlorobiales* . Genome pairs sharing higher amino acid identity are shaded more darkly (red). The regions of the matrix corresponding to species from different proposed families are labeled

Group	Organism	Accession	248		314
	Chlorobium luteolum	78185909	EFEQMEMQYFVKPGTQAEAFEAWREERYQWY	T	RQLGIRPEKLHWYKHDKLAHY
	Chlorobium chlorochromatii	78188020	----------------L----------FA--	V	NS---T-S-------------
	Chlorobium phaeovibrioides	145218843	----------------V----------FN--	-	SS---S---------------
	Chlorobium phaeoclathratiforme	194335210	---------------------------FA--	-	SA--L-K--------------
	Chlorobium phaeobacteroides	119355895	---------------------------FN--	-	SA---NRD-------------
	Chlorobium limicola	189345581	--------------S-I----------FN--	V	TE---TQ--------------
	Chlorobaculum parvum	193211698	----------------L----------FR--	S	ET--MTK--------------
	Chlorobaculum tepidum	21675063	----------------L----------FR--	S	ET--MSK--------------
	Prosthecochloris aestuarii	194332877	----------------K-T--------FN--	K	ER--LT-G-------------
	Chlorobium ferrooxidans	493410795	----------------KG---------FA--	S	TA--L-A--------------
**Order *Chlorobiales***	*Chloroherpeton thalassium*	193214675	----------------M----E---R-MS--	-	ES---SK---------Q----
	Chlorobaculum limnaeum	2501895760	----------L-----L----------FR--	S	ET--MTK--------------
	Candidatus Chlorobium masyuteum	166807360	----------------K----------FA--	S	AT--L-A--------------
	Chlorobaculum thiosulfatiphilum	170181247	----------------L----------FR--	S	ET--MTR--------------
	Prosthecochloris marina	161953525	----------------G-S---------N--	I	EK---ER--------------
	Candidatus Thermochlorobacter GBChlB	KER10651	----------I---A-K---SM-Q-K-FN--	A	DY---NT-N-V--Q-------
	Candidatus Thermochlorobacter aerophilum	RFM23176	-----------R--E-MA---M-Q-Q-MK--	-	EC--FKK-N-V----------
	Chlorobium sp. N1	165498823	--------------------T------G--	-	A---LS---------------
	Chlorobium sp. KB01	076789630	----------------K----E--T--FA--	S	TT--L-A--------------
	Chlorobium sp. 445	PI048809	--------------V-L----M-Q-R-MK--	A	ER--LK--N-S----------
	Prosthecochloris sp. HL-130-GSB	085658738	----------------L-T--------FN--	V	KS---S-D-----R-------
	Chlorobaculum sp. 24CR	167599212	----------------L----------FR--	S	ET--MTR--------------
	Prosthecochloris sp. ZM	114617902	----------------K-T-DA-----FN--	K	ER--LT-G-------------
	Prosthecochloris sp. GSB1	094081604	--------------A-E----------FN--	V	ER---GQ------R-------
	Chlorobi bacterium OLB4	KXK01802	--------F-----DDS-W--Y-K-Q-I--F		EKY-M-K---KF-E-E-----
**Larger *Ignavibacteriae* Clade**	*Chlorobi bacterium OLB6*	KXK33418	--------F-IA----D-W--Y--AA-WE-L		VN--LT--N-RRKP-E-----
	Chlorobi bacterium OLB7	KXK53003	--------F-----ED-KWYDY---Q-WN--		-D--MQM-R-RMKP-E-----
	Segetibacter koreensis	517441118	--------F--R---EG-WYRY-K-A-L--H		LS---S-K-YRYHD-V-----
	Solitalea canadensis	387789634	--------F--R---EM-WYNY-K-A-LK-H		KA--TD---YRYHD-T-----
	Hymenobacter norwichensis	551197521	--------F--R---EG-WY-H-KAT-RA-H		EAM--PTN--RFHD-------
	Arcticibacter svalbardensis	510820536	--------F--R--EDKKW-DY-KIA-LK-H		TA--TSS--YRFHD-V-----
	Mucilaginibacter paludis	495781065	--------F-IR---EM-WY-Y-KAA-LK-H		VA--TD-A-YQYHD-A-----
	Flexithrix dorotheae	522015193	--------F--R--SEM-WY-K-KAQ-MA-H		TV--TD----RFHD-------
	Joostella marina	495886513	--------F-I-----K-WYDY-K-T-LK-H		MS--MGE-NYRFHD-E-----
	Chitinophaga pinensis	256419457	--------F--R----K-W-AY-K---LK-H		KS---D-D-YRFHV-----F-
	Pedobacter heparinus	255533134	--------F--R--EDKKW-AY-K-A-LK-H		LA--TPA--YRYHD-V-----
**Other Bacteria**	*Porphyromonas endodontalis*	490463729	--------F--A--EEMKW--Y-KKT-MAFH		KS--LGDN-YRYHD-E-----
	Dyadobacter fermentans	255038651	--------F--R---EQ-WY-K-K-A-LKFH		KAI-LPA---KYHD-------
	Dyadobacter beijingensis	518785017	--------F--R---EE-WYNK-K-A-LKFH		KAI-LPA---KYHD-------
	Anaerophaga thermohalophila	515149603	--------F--R--EEM-W-NY-KQV-MK-H		KA--MGED-YRFHD-------
	Flavobacterium columnare	365960887	--------F-----EEMKWY-Y-KQT-LK-H		KS--LGA-NYRYHD-E-----
	Lewinella cohaerens	522025975	--------F-IQ----KKWYDY-K-A-LK-H		LA--TSAS-YRFHD--N----
	Paludibacter propionicigenes	313204242	--------F--R--EEMKW--H-KAF-LK-H		KA--LGDQ-YRFHD-------
	Porphyromonas catoniae	491610397	--------F--R--EELKW-DF-KDL-LR-H		KA--LGDK-YRFHD-E-----
	Anaerophaga thermohalophila	498104982	--------F--R--EEM-W-NY-KQV-MK-H		KA--MGE--YRFHD-------
	Mariniradius saccharolyticus	495906476	--------F--R--SELDWYKE-AAT-M--H		KA---PE---RRHD-E-----

Figure S4

Partial sequence alignment of the protein Glycine-tRNA synthetase showing a 1 aa insertion (boxed) that is specific for the order *Chlorobiales*

Group	Species	Accession	24		79
Order *Chlorobiales*	*Chlorobaculum parvum*	193212077	EIGKYDLLTAEDEVKLTMAIKEG	YDMPVDTVEYKR	AKRALDKLIKGNLRFVVSVAK
	Chlorobium limicola	189345950	----------D------------	------------	---------------------
	Prosthecochloris aestuarii	194334634	-----E-----------------	-------P----	---------------------
	Chlorobium phaeoclathratiforme	194337394	-----------------K-----	F------T----	---------------------
	Chlorobium ferrooxidans	493409341	-----------------K---D-	F------T----	---------------------
	Chlorobium chlorochromatii	78188365	--------------R--K-----	F------P----	---------------------
	Chlorobium luteolum	78186275	----------D---R--K-----	F------T----	---------------------
	Chlorobium phaeobacteroides	189500956	-----E-----------K---A-	-------T----	---------------------
	Chlorobium phaeovibrioides	145219258	----------D---R--K-----	F---L--T----	---------------------
	Chlorobaculum tepidum	1519644	----------RRRGEAHEGH---	------------	---------------------
	Chlorobaculum limnaeum	2501895477	-----------------K-----	------------	---------------------
	Prosthecochloris marina	110022354	-----E-----------K-----	-------P----	---------------------
	Chloroherpeton thalassium	193216316	----F-----QE-IE--RQ--K-	DGQ-I-SK--RE	-R---E----A----------
	Chlorobium sp. N1	131356747	----------D---R--K-----	F------T----	---------------------
	Chlorobium sp. 445	PI048482	----FE----Q--IE--RK--R-	EGKS--SK--RE	-Q---E----A----------
	Cand. Thermochlorobacteria aerophilum	KER10064	----F-----Q--IT---K--K-	DGK---SK-F-E	GR---E----A----------
	Chlorobaculum sp. 24CR	129052373	-----------------------	----------R-	---------------------
	Prosthecochloris sp. HL-130-GSB	085660123	-----E-----------R-----	-------P----	---------------------
	Prosthecochloris sp. GSB1	094083210	-----E-----------K-----	-------P----	---------------------
Larger *Ignavibacteriae* Clade	*Chlorobi bacterium OLB6*	KXK33417	---RV----GD--IT-AQ---R-	GFE	GGL--ER-V-A----------
	Chlorobi bacterium OLB5	KXK55587	----VE--DV-E-ID-AKK--Y-	DE-E	KQK--E--T-A----------
	Chlorobi bacterium OLB7	KXK54381	----VE---PDM-IE-AKR--R-	GAE	GE---EQ-V-A----------
	Chlorobi bacterium NICIL-2	KXB98252	----IE--SP-E-IE-ARK-RQ-	GPE	GEA--E--V-A----------
	Melioribacter roseus	397689183	----VE---P---IE-AIK--K-		DQK--E--V-A----------
	Ignavibacterium album	385811725	----V----PQ--ID-AIR--K-		DEK-KE----A----------
Other Bacteria	*Gillisia marina*	497916484	----V--I---E--E-AQK--A-		DD---E--T-A----------
	Nafulsella turpanensis	516339844	----V----PDE--E-AKR-R--		DQM--E--T-A----------
	Psychroflexus tropicus	517868227	----V--I---E--E-AQR--A-		DD---E--T-A--------S-
	Gramella forsetii	120434745	----V--I--DE--E-AQR--A-		DD---E--T-A----------
	Fulvivirga imtechensis	497263748	----V----PDE--E-AQR----		DQL--E--T-A----------
	Zhouia amylolytica	568803407	----V--I---E--E-AQR--A-		DQ---E--T-A----------
	Polaribacter porphyrae	105016810	-VS-I--I--DE--E-AVK----		SQ------V-A----------
	Sediminibacterium ginsengisoli	217698805	----V--ISP-E--Q-S-R-R--		CQ-S--R---S----------
	Tenuifilum thalassicum	173075478	----V------E--T-ARK----		DQE--E--V-A----------
	Saccharicrinis aurantiacus	075601683	----VK-----E----AKR--D-		DTV---R--NA----------
	Labilibaculum manganireducens	101308830	----V--IS--E--Q-ARE--K-		N-K---R---C--------S-
	Flavobacterium psychrophilum	123910415	----V--I--DE--E-AQR--A-		DQ---E--T-A----------
	Myroides marinus	074744386	----V--I---E--E-AQR--A-		DQ---E--T-A----------
	Aureitalea marina	104811626	----V--I---E--E-AQR--A-		DQ---E--T-A----------
	Planktosalinus lacus	188440396	----V--I--DE--E-AQR--K-		DQ---E--T-A----------
	Tenacibaculum skagerrakense	132792729	D---I--I--DE--E-SQQ--K-		NQ---E--VNA----------
	Psychroflexus salis	188406779	----V--I---E--E-AQR--A-		DDL--E--T-A--------S-
	Massilibacteroides vaginae	085537662	---RE--I-V-E--E-AQ---R-		DR---E--TRA----------
	Parabacteroides distasonis	121736482	---RE--I-V-E--E-AQ---R-		DR---E--TRA----------
	Salmonella enterica	MBH0619490	----V----PDE--T-AQR-RD-		DQK--E--T-A----------
	Chryseolinea serpens	SHG77818	---RV--I--DE-----QR--A-		DQA--E-MV-A----------
	Pukyongia salina	105216487	----V--I--DE--E-AQR--A-		DQ---E--T-A----------
	Flavobacterium haoranii	072781367	----V--I--DE--E-AQR--A-		DQ---E--T-A----------
	Marinirhabdus gelatinilytica	115124369	----V--I--DE--E-AQK--A-		DQ---E--T-A----------
	Catalinimonas alkaloidigena	089683757	----V----PDE--E-AKR-R--		DQ---E--T-A----------
	Ancylomarina salipaludis	129253240	----V--V---E--A-ARQ--K-		N-T--NR---A--------S-
	Imtechella halotolerans	008238527	----V--I--DE--E-AQR--A-		DQ---E--T-A----------
	Dokdonia pacifica	089373128	----V--I--DE--E-AQR--A-		DQ---E--T-A----------
	Ginsengibacter hankyongi	150415004	----V--I-P-E----ALR--Q-		DQQ-----T-A----------
	Pustulibacterium marinum	093022816	----V--I--DE--E-AQR--A-		DQ---E--T-A----------
	Robertkochia marina	136335350	----V--I--DE--E-AQR--A-		DQ---E--T-A----------
	Tenacibaculum adriaticum	148869371	----V--I--DE--E-AQR--A-		DQ---E--T-A----------
	Polaribacter porphyrae	105017184	----V--I--DE--E-AQL--A-		DQ---E--T-A----------
	Tenacibaculum dicentrarchi	101902490	----V--I--DE--E-AQR--A-		DQ---E--T-A----------
	Hymenobacter norwichensis	551195676	----V----PDE--T-AQR----		DQQ--E--T-A----------
	Cesiribacter andamanensis	496485220	----V----PDE--E-AKR-RQ-		DQM--E--T-A----------
	Porphyromonas macacae	517172454	---RE--I-V-E--E-AQ---R-		DR---E--TRA----------
	Rhodonellum psychrophilum	518430040	----V-----DE--V-AKR-R--		DQL--E--T-A----------
	Belliella baltica	390944276	----V-----DE--V-AKR-R--		DQL--E--T-A----------
	Microscilla marina	488786349	----V----PDE--E-AKR-R--		DQV--E--T-A----------
	Echinicola vietnamensis	431798761	----V-----DE--V-AKR-R--		DQL--E--T-A

Figure S5

Partial sequence alignment of the protein RNA polymerase sigma factor RpoD/SigA showing a 12 aa insertion (boxed) that is exclusive to all members belonging to the order *Chlorobiales*

			284		333
	Chlorobium limicola	189347814	QGNLDPTVLYGTPEKIKSEAAKVLKQFGQ	HTE	SSGHVFNLGHGILPDVDP
	Chlorobium phaeoclathratiforme	194337695	-------------DR--A--S-I--S---	---	H--------------M--
	Chlorobium ferrooxidans	493409671	-------------AL-----S-I--S---	---	H--------------M--
	Chlorobaculum parvum	193213554	---M------------------I------	--D	C-----------------
	Chlorobaculum tepidum	21674849	---M-------------------------	-ND	H-----------------
	Chlorobium phaeobacteroides	119356021	---M---------D--R-----I------	--A	T-----------------
	Chlorobium luteolum	78187834	---M-------------A------R----	--A	Q-----------------
	Chlorobium phaeovibrioides	145220438	---M-------------A--G-I------	--A	N-----------------
	Prosthecochloris aestuarii	194333095	---M--------HD---A----I------	--A	E-----------------
	Chlorobium phaeobacteroides	189499267	---M--------HD--RA--G-I-ER---	-S-	C-----------------
	Chlorobaculum thiosulfatiphilum	139456015	---M-------------------------	-ND	H-----------------
	Prosthecochloris ethylica	175187185	---M---I----HDR--A----I------	--Q	K-----------------
**Order *Chlorobiales***	*Chloroherpeton thalassium*	193214637	----------AS--R-RE--G-I-AK---	---	K---------------N-
	Chlorobium chlorochromatii	78188113	------------Q-R--I--G-I--S---	-NH	H--------------M--
	Chlorobaculum limnaeum	2501895585	---M------------------I------	---	N-----------------
	Candidatus Thermochlorobacter. GBChlB	KER09257	-------I--AS--R-REH--FI---F-E	--D	T--------------IE-
	Candidatus Chlorobium masyuteum	166807571	------------QAL--A--SKI--S---	TH-	H--------------M--
	Prosthecochloris marina	110022929	---M--------HD--RA----I-ERF-K	--D	T-----------------
	Chlorobium sp. 445	PI048272	-------I--APHDV-RK----I-QRF-E	-SD	---------------I--
	Chlorobium sp. KB01	076789795	-------------AL--A--SKI--SF--	TH-	H--------------M--
	Chlorobaculum sp. 24CR	129052099	---M--------------------R----	-ND	H-----------------
	Chlorobium sp. N1	131355033	---M-------------A----I------	--A	Q-----------------
	Prosthecochloris sp. ZM_2	114607870	---M---I----HDR--A----I------	--Q	K-----------------
	Prosthecochloris sp. GSB1	094081779	---M---A----H-R-RA----I-Q----	---	K-----------------
	Prosthecochloris sp. HL-130-GSB	085658882	---M---M----HDR-RA----I-R----	--G	N-----------------
Ignavibacteriae	*Ignavibacterium album*	385809123	----------ANKNY--Q-VIS--QS--E		G---I------V------
	Melioribacter roseus	397689611	----------AGKD--FE-TVRT-NSY-K		GP-------------I--
	Microbulbifer variabilis	521082728	------S---AS--R-RQ-VETI-QA--H		G-----------T-K---
	Flagellatimonas centrodinii	228348101	------S---AS--R-RL-V-R--QD--N		G-----------T-G-N-
	Pseudomonas putida	167036113	---M------AK--A-RQ-V-RI-ASY--		GT----------T-E---
	Arsukibacterium ikkense	046558556	---M--S--L-S--R-RQ-VQSI-DSY--		G-----------T-----
	Piscirickettsia salmonis	017376242	---I--A------AG-RH-V-R--GE--H		G-----------Y--IN-
	Marinobacter nanhaiticus	490718280	---M--SM--AP--R-RQ-V-DI-SRY-E		G---I-------T-----
	Pseudomonas fuscovaginae	516515253	---M------AR--A-RA-V-RI-ASY-E		G-----------T-E---
	Marinobacter hydrocarbonoclasticus	387813140	---M--AM--APKAR-RQ-V-DI--RY-S		G-----------T-----
	Azomonas macrocytogenes	183165880	---M------AN-QA-RA-VGRI-ERY-K		G-----------T-E---
	Teredinibacter waterburyi	188150035	---M--S---AS-DR-RA-V-RI-SE--R		GN----------T-E---
	Marinobacter aquaeolei	120553768	---M--AM--APKAR-RQ-V-DI--RY-S		G-----------T-----
	Microbulbifer agarilyticus	497818265	---M--SI-LAN--R-RE-V-SI-ASY-R		G---I------VT-K---
	Gilvimarinus chinensis	519053976	---M---I--AS--R-RE-VGRI-SSY-S		G---I-------T-E---
**Other Bacteria**	*Pseudomonas aeruginosa*	564955370	---M--S---AN-AA-RA-V-RI-AAY-E		GT----------T-E---
	Thioflavicoccus mobilis	431932858	------C---AS--Q-RA-V-R--ESY-R		GH----------T-----
	Teredinibacter purpureus	045857385	---M---I--AS-DR-RE-VGRI--SY-A		G-----------T-E---
	Hydrogenovibrio kuenenii	024850106	---M--SI--AS--R-RQ-V-TI-EKY-K		G-----------H-E---
	Cellvibrio zantedeschiae	189419012	---M---I--AS--R-RT-VGDI-QAY-K		G-----------T-E---
	Priestia aryabhattai	QPN45859	---M------AK--A-RN-V-RI-ASY-K		GT----------T-E---
	Rheinheimera nanhaiensis	008223154	---M--AI-L-S--R-RQ-VQMI-DG---		G-----------T-----
	Idiomarina loihiensis	56461406	---M--S---AK-AR-EE-VQSI-QSY-S		GT----------H-E---
	Pseudomonas syringae	330938623	---M------AR--A-RQ-V-RI-ASY-S		GT----------T-E---
	Thiococcus pfennigii	MBK1702036	------C---AS--R-RA-V-R--ESY-R		GH----------T-----
	Microbulbifer taiwanensis	193190112	------A---AS--R-RA-V-A--ES--S		G-----------T-K---
	Alishewanella longhuensis	189433774	---M--S--L----R-RQ-VQTI-DG---		GT--I-------T-----
	Gilvimarinus chinensis	020209851	---M---I--AS--R-RE-VGRI-SSY-S		G---I-------T-E---

Figure S6

Partial sequence alignment of the protein uroporphyrinogen decarboxylase, showing a 3 aa insertion (boxed) that is exclusive to all members belonging to the order *Chlorobiales*

Group	Species	Accession	211		250
Order *Chlorobiales*	*Chlorobium phaeoclathratiforme*	194336061	ITNRTLSKAETLAEELGTNKV	L	PFETFREHLHEFDIIITA
	Prosthecochloris vibrioformis	139626275	-----I-------------Q-	-	----------D-------
	Chlorobium chlorochromatii	78189476	-----I----A--------Q-	-	-Y-SYK------------
	Chlorobium limicola	189346423	----------A-------G--	-	---SYK-Y----------
	Chlorobium luteolum	78187285	-----Q----A--------R-	-	---SYK------------
	Chlorobium phaeobacteroides	189499890	----------A--------Q-	-	-Y-SYKD-----------
	Chlorobaculum tepidum	21674247	-----Q----A--------R-	-	-Y-SYK------------
	Chlorobaculum parvum	193212402	-----Q----A--------R-	-	-Y-SYK------------
	Chlorobium ferrooxidans	493411236	------A---A-S--------	-	-Y-SYK------------
	Prosthecochloris aestuarii	194334345	-----M----A--------Q-	-	------DN----------
	Chlorobium phaeovibrioides	145220049	-----R--S-A--------Q-	-	-Y-SYK------------
	Chlorobium phaeobacteroides	119357592	----------A-------K--	-	---SYKDY----------
	Chloroherpeton thalassium	193215988	-----YE---L--Q----G-I	-	-L-EMT-R------VV--
	Chlorobaculum limnaeum	501894998	----------A--------R-	-	-Y-SYK------------
	Prosthecochloris vibrioformis	139626275	-------------------Q-	-	----------D-------
	Chlorobaculum parvum	012501988	-----Q----A--------R-	-	-Y-SYK------------
	Chlorobaculum thiosulfatiphilum	139457331	-----Q----A--------R-	-	-Y-SYK------------
	Chlorobium limicola	012465852	----------A-------G--	-	---SYK-Y----------
	Prosthecochloris marina	110023205	----------A--------Q-	-	-Y-SYK------------
	Candidatus Chlorobium masyuteum	166808915	------A---A-S--------	-	-Y-SYKD-----------
	Cand. Thermochlorobacter aerophilum	RFM23265	-----PEH--A--RKI--SNI	I	T-DY---R------V-S-
	Chlorobium sp. N1	131353836	-----Q----A--------R-	-	---SYK------------
	Chlorobium sp. KB01	076790786	------A---A-S--------	-	-Y-SYK------------
	Chlorobaculum sp. 24CR	129052243	-----Q----A--------R-	-	-Y--YK------------
	Prosthecochloris sp. CIB 2401	068866687	-----I-------------Q-	-	----------D-------
	Prosthecochloris sp. HL-130-GSB	085659333	-----M----A--------Q-	I	------D-----------
	Prosthecochloris sp. GSB1	094082178	----------A--A-----Q-	-	---SYK------------
Ignavibacteriae	*Ignavibacterium album*	385809127	L----FE---K--T--N-AVF		--D--K----K-----S-
	Melioribacter roseus	397689614	-A---MER--K---KVNGGVI		--PSLK----NY----S-
Other Bacteria	*Denitrovibrio acetiphilus*	291288797	V----M----K--C-V-GDTL		---SY--S-EKY--V-SS
	Deferribacter desulfuricans	291279208	V----F-R--I----INGEAI		--DN-YNK-N-V--V-SS
	Flexistipes sinusarabici	336323776	V----Y-R--K--Q-INGIP-		D--R-T-Y-KKT--V-SS
	Calditerrivibrio nitroreducens	313673929	V----FER-LQ--K-VNGEA-		-I-K-V-Y-K-V--V-SS
	Mucispirillum schaedleri	555554431	V----FE--LK----FSGDA-		T-DR-AQK-P-I----SS
	Thermodesulfovibrio yellowstonii	206890822	L----FQR--EI-K-FNGVA-		--GNLK-Q-VKT--V-CS
	Geobacter lovleyi	189423574	V----FER-QR----F-GEAI		--DELFL---KA--VL-S
	Geopsychrobacter electrodiphilus	522166020	V----FAR--K--T-FKGRA-		A--N-QDQ--QA--VLSS
	Desulfuromonas acetoxidans	492848965	V----FAR--K--S-FSGQAI		L--N-HD--QQV--LLSS
	Desulfovibrio desulfuricans	518845794	VA---FARGQA---QFNGEAI		--DQLF-Q-EKV----SS
	Geobacter uraniireducens	148262443	V----FER-VK----FDGKA-		NY-ELFD---KA--VLSS
	Pelobacter propionicus	118581777	VA---FA-G-R--A-F-GEAI		---ELFQE--RA--VLSS
	Geobacter metallireducens	404498082	V----YER-VK----FEGKP-		S-DDLFDQ--KA--VLSS
	Bilophila wadsworthia	491168655	VA---YQR-KE--A-FKGEA-		---HLF---ADV----SS
	Geobacter bemidjiensis	197116805	V----YER--K----FDAKP-		H--ALM-T-PKA---LSS
	Desulfovibrio gigas	545642716	VA----ER-VH---QFRGEA-		---LLH-R---V----SS
	Geobacter daltonii	222054399	V----FER-VK----FDGKA-		NY-DLFDQ--KA---LSS
	Methanocaldococcus jannaschii	15668315	VA---YE--K--K---GMAI		K-DKLE-A-RYA--V-S-
	Methanotorris formicicus	494103966	-A---YER--C--R--RGIAI		H-DKL--A-KYA--V-S-
	Algoriphagus aestuariicola	206567720	-----Q----QI-A---FEV-		---NC--AMN-A-V-VCS
	Xanthovirga aplysinae	155206362	-----F----S--A-C-LKTI		---QTF-VMKD--V--SS
	Pontibacter roseus	018476482	------T--QH----C-LEVL		---DMVQG-K-A-VV-SS
	Botryobacter ruber	114777573	------A--VP----C-MEVL		---DIVQG-K-A-V--SS
	Desulfuromonas soudanensis	053551860	V-----ER-VK----FNGRAL		---D-TH--QQV---L-S
	Taibaiella koreensis	118973184	LI---EG--QA------LHTA		---ML-QYIR-C----V-
	Aeromonas diversa	005351703	VA----QR-QG----F-AQVM		TL-AIP-----A--V-SS
	Methanothermococcus okinawensis	336121948	VA---YER--R--N--KGMA-		H-DKLN-A-NFS----C-
	Methanococcus voltae	297619964	-S---YER--R--K--KGIAI		N-DKLK-AINYS----C-
	Nitritalea halalkaliphila	496347363	-A---FE--QA--AG--YSVI		--DEVN-AMAAA-VVVSS
	Mariniradius saccharolyticus	495899215	-----FE--KE--D---FEVI		---RCV-AMKDA-VVVSS
	Microscilla marina	488784857	-C----A---K--A-C-AKVI		---NV-QAIL-A--V-SS
	Chryseobacterium taeanense	515974948	-A----ERG-K--AQF-AGTM		RLADLP-----Y-AV-S
	Nitrospina gracilis	491151936	VAS--YER-AG--KT-NGSAL		G-DE-K-E-YRA--VVSS
	Magnetococcus marinus	117926918	V----F-R-VD--QQFDGHAF		-I-ALA-N--RA--V-SS
	Photobacterium damselae	491440282	VA---RER--KFST-F-AEVI		GL-QIP----RA--V-SS
	Aeromonas diversa	491493979	VA----QR-QG----F-AQVM		TL-AIP-----A--V-SS
	Photobacterium leiognathi	496274273	VA---RER--GI-R-F-AEVI		SL-EIP----RA--V-SS
	Alteromonas macleodii	406596336	VA---VAR--A---T-DASVF		TLSQVP---KD---V-SS
	Acidovorax ebreus	222109996	-A----ERG-K--TRF-GEVM		RLADLPD------AV-S

Figure S7

Partial sequence alignment of the protein glutamyl-tRNA reductase, showing a 1 aa insertion (boxed) that is exclusive to all members belonging to the order *Chlorobiales*

			60		109
**Order *Chlorobiales***	*Chlorobaculum tepidum*	21674908	GSWGPFILGSMHPRVTAAIEYTLR	N	IGTSFGTPIELEIEIAELLCKIVPS
	Prosthecochloris vibrioformis	139626633	--------------I---L-H--T	K	----------M--------TQ----
	Chlorobium ferrooxidans	493409722	--------------I---L----K	-	----------M--------------
	Chlorobium phaeoclathratiforme	194337772	--------------I---L----K	-	----------M--------------
	Chlorobium chlorochromatii	78189921	--------------I---L----K	-	----------M---------Q----
	Chlorobium phaeobacteroides	119358325	--------------I---L----K	-	----------M--------------
	Chlorobium limicola	189347866	-----------Q------------	-	--S-------M--------SR----
	Prosthecochloris aestuarii	194333039	--------------I---L-H--T	K	----------M---------Q----
	Chlorobium luteolum	78187880	--------------I---L-H--T	K	----------M--------T-----
	Chlorobium phaeobacteroides	189499186	--------------I-----N--T	K	----------M--------TQ----
	Chlorobium phaeovibrioides	145220484	--------------I---L-R--T	T	----------M--------VE----
	Chlorobaculum parvum	193211823	--------------L---------	-	----------I---------Q----
	Chloroherpeton thalassium	193213745	---------HS----IE---R-MK	A	H-----A-T-----L---II-V---
	Chlorobaculum limnaeum	2501894144	--------------L---------	-	-------------------------
	Chlorobaculum thiosulfatiphilu	139457606	--------------L---------	-	---------------------V---
	Pelodictyon phaeoclathratiform	012509417	--------------I---L----K	-	----------M--------------
	Prosthecochloris marina	110022620	-------------K----L-H--T	K	V------------------T-----
	Cand. Thermochlorobacter aerophilum	RFM25453	---------HS----V----H--K	H	----A---------L---ISRL---
	Candidatus Chlorobium masyuteum	166807515	--------------I---L----K	-	----------M--------------
	Chlorobium sp. 445	PI047274	---------HA----V----H--K	H	V-----A-T----QL---ISTL---
	Chlorobium sp. N1	131355189	--------------I---L-F--T	K	-------------------TR----
	Prosthecochloris sp. GSB1	094081727	--------------I---L-H--T	K	----------M---------Q----
	Prosthecochloris sp. ZM	114617802	--------------I---L-H--T	K	----------M---------Q----
	Prosthecochloris sp. CIB 2401	068868140	--------------I---L-H--T	K	----------M--------TQ----
	Prosthecochloris sp. HL-130-GSB	085658843	-------------K----L-H--T	K	----------M-----Q--TQ----
	Chlorobaculum sp. 24CR	129052311	--------------L---------	-	--------------------Q----
Ignavibacteriae	*Melioribacter roseus*	385811690	-----HLF-HNP-FIKE-LLKA-E		D-----A-T-I-VKM---I-DL---
	Ignavibacterium album	397690482	-----HLF-HNP-FIKK-LAEAFE		K-----A-T---VRM-Q-ITEL---
**Other Bacteria**	*Thermanaeromonas toyohensis*	084665710	-----L---HR--E-V--L-K--K	E	M-----A-T---N-L-KMITQA---
	Pelotomaculum thermopropionicum	147677314	C----L---HR----VG-L-RC-N	E	V-----A-T---NIL--MIVEA---
	Pelotomaculum propionicicum	134213985	-----L---HR--HAVE-LERC--	D	V-----A-T-M-TKL-SMVAEA---
	Burkholderia phytofirmans	187925072	-----M---HV--E-LE-VQRV-G		N-F---A-T-S-V----EI--L---
	Clostridium straminisolvens	575087331	-----M---HS----LE--SK-MV		D-L---AAT---VQM---ILEL---
	Brevibacillus panacihumi	559035961	-----L---HA--Q-L---TEVAA		L-----A-T-R-T-M-K-V-QL---
	Bacillus cereus	507065470	-----L---HA--E-VK--QEAAS		Q-----L-T-A-TKM---I-RSI--
	Histophilus somni	136121970	-----M---HN--SILS-VLK-AE		N-L-----TP----L---I-QL---
	Chloracidobacterium validum	211427812	-----M---HA--E-L---RSVIE		R-----A-T---V-----IVS----
	Andreprevotia chitinilytica	035054799	-----L---HA--A-ID-V-RAA-		N-L---A-T-A--DL-D---ELL--
	Chitinolyticbacter meiyuanensis	148713814	-----L---HA--A-IE-V-R-I-		N-L---A-TAA---L-D---QQL--
	Nitrosovibrio tenuis	090829456	-----L---HA--E-IE-V-AAAQ		N-LT--A-T-A-L--------L-S-
	Formivibrio citricus	091194206	-----L---HA--A-VE-V-RAA-		N-L---A-T-A--DL-D---ELL--
	Gallibacterium salpingitidis	066111049	-----MV--HN--TILQ-VID-AK		N-L---A-T----KL---V--L---
	Azonexus hydrophilus	028993650	-----M---HA--Q-I--VQEAVV		D-L---A-T-R-V---D---EM---
	Brevibacillus antibioticus	137029901	-----L---HA----L---TEVAA		L-----A-T-R-T-M-K-V-E----
	Nitrosospira multiformis	041352651	-----L---HA--E-VD-VYAAAK		N-LT--A-T-A-L-------RL---
	Chloracidobacterium aggregatum	211426713	-----M---HA--E-L---RSVIE		R-----A-T---V-----IVSL---
	Caballeronia terrestris	087655126	-----M---HV--E-LD-VQRV-G		N-F---A-T-A------EI--L---
	Brevibacillus laterosporus	517502562	-----L---HA--K-LQ--SE-AL		L-----A-T-R-TTM-K-V-D----
	Pasteurella multocida	378774174	-----M---HN--TILD-VIS-AQ		N-L---A-TA----L---V-SL---
	Paenibacillus ginsengihumi	518363260	-----L-V-HA----VE-VQRAAE		A-----A-T---T-M-K-VAER---
	Cohnella laeviribosi	517834161	L----L-A-HA--E-VE--KR-AE		K-----A-T---T-M---V-ER---
	Gallibacterium anatis	545092524	-----MV--HN--SILN-VIE-AK		N-L---A-T-----L---V-RL---
	Haemophilus somnus	113461372	-----M---HN--SILS-VLK-AE		N-L-----TP----L---I-QL---
	Gallibacterium anatis	517158368	-----MV--HN--SILN-VIE-AK		N-L---A-T-----L---V-RL---
	Paraburkholderia silvatlantica	133790131	-----M-V-HV--E-LD-VQRV-A		N-F---A-T-A------EI------
	Aggregatibacter segnis	493770001	-----M---HN--TILN-VMK-AQ		N-L---A-TP----L---V-ELI--
	Avibacterium paragallinarum	516416719	-----M---HN--AILS-VQK-AE		K-L---A-TP---DL---V-QL---
	Pasteurella multocida	512748104	-----M---HN--TILD-VIS-AQ		N-L---A-TA----L---V-SL---
	Aggregatibacter actinomycetemcomitans	491728551	-----M---HN--SILN-VIK-AQ		N-L---A-TP----L---V-SL---
	Avibacterium paragallinarum	545595327	-----M---HN--AILS-VQK-AE		K-L---A-TP---DL---V-QL---
	Planctomyces brasiliensis	325111308	-----H---HQ----L---QEE-S		K-----A-TA--TDM---V-EC---
	Paenibacillus vortex	493234377	-----L-M-HA--E-VD-LQE-AA		K-----A-TL--T-M-K-V-ER---
	Planctomyces limnophilus	296122991	-----H---HR--V-----HAAVD		R-----A-C-A-N-L-QMVIEA---
	Desulfotomaculum kuznetsovii	333978951	-----L---HR--E----LQEC-E		------A-T-----L-RAIVEAL-A
	Clostridium thermocellum	125975015	-----M---HS----LE-VSK-MV		D-L---AAT---VQM---ITEL---
	Geobacter uraniireducens	148266255	-----M---HC--Q-V--VKSAVD		N-C---A-T----TL--MVIEA---
	Burkholderia kururiensis	516384471	-----M-V-HV--E-LE-VQR--V		K-F---A-T-A------VI--L---
	Haemophilus somnus	170719024	-----M---HN--SILS-VLKIAE		N-L-----TP----L---I-QL---
	Burkholderia xenovorans	91784895	-----M---HV--E-LE-VQRV-G		N-F---A-T-S-V----EI--L---
	Nitrosomonas europaea	30249394	-----L---HA--D-VR-VQIAAG		H-----A-TAA-L-------RLL--

Figure S8

Partial sequence alignment of the protein glutamate-1-semialdehyde 2,1-aminomutase showing a 1 aa insertion (boxed) that is found in all members belonging to the order *Chlorobiales*. The CSI is not present in any other bacterial species apart from the three exceptions shown above, in the top 500-BLASTp hits.

Group	Organism	Accession	233		282
**Order *Chlorobiales***	*Chlorobium phaeobacteroides*	189500266	DSLAISIGTSHGAFKFKPGE		DPKIRLDILEEIEKRIPGFPIVLHGSSSVP
	Chlorobium chlorochromatii	78186883	--------------------		---------A--------------------
	Chlorobium phaeovibrioides	145219699	--------------------		---------Q--------------------
	Chlorobium chlorochromatii	78188704	--------------------		---------A--------------------
	Chlorobium ferrooxidans	493411307	--------------------		---------S---R----------------
	Chlorobium phaeobacteroides	119357140	--------------------		---------T---R----------------
	Chlorobium limicola	189346770	--------------------		---------S---Q-----------A----
	Chlorobium phaeoclathratiforme	194336449	--------------------		---------T---H----------------
	Prosthecochloris aestuarii	194333871	-----A--------------		-H-------S--------------------
	Chlorobaculum parvum	193212638	-----A--------------		-H-------A---------------A----
	Prosthecochloris marina	110021934	-----A-------Y------		------------------------------
	Chlorobaculum tepidum	21673879	-----A--------------		-H-------A---------------A----
	Chlorobaculum limnaeum	2501894580	-----A--------------		EHS------A---------------A----
	Pelodictyon luteolum	011357757	--------------------		-------------------------A----
	Prosthecochloris ethylica	175187116	-----A-------Y------		---------A---Q----------------
	Chlorobaculum thiosulfatiphilum	139457515	-----A--------------		EHS------A---------------A----
	Prosthecochloris sp. GSB1	094082604	--------------------		-------------Q----------------
	Prosthecochloris sp. HL-130-GSB	085659640	-----A--------------		---------A--------------------
	Chlorobium sp. N1	131353286	--------------------		---------A---------------A----
	Prosthecochloris sp. ZM_2	114606996	-----A-------Y------		---------A---Q----------------
	Chlorobaculum sp. 24CR	129051447	-----A--------------		EHL------R---------------A----
Ignavibacteriae	*Melioribacter roseus*	397689677	-------------N----EQ	CTRN EDGILI	P-PL-F-------------------A----
**Other Bacteria**	*Bacteroides pyogenes*	545405147	-------------Y--T-EQ	CHVDPATGRMV	P-PLAF-V-DAVM-EL--------------
	Barnesiella viscericola	570742111	-------------Y----EQ	CHVDPATGRLV	P-PLAF-V-DAVMEKL--------------
	Bacteroides salanitronis	325299001	-------------Y----EQ	CHVDPKTGRLV	P-PLAF-V-DAVMEKL--------------
	Barnesiella intestinihominis	496138225	-------------Y----EQ	CHVDPKTGRLV	P-PLAF-V-DAVMEKL--------------
	Parabacteroides distasonis	150008558	-------------Y--T-EQ	CTIDPVTGKMV	P-PLAF-V-DAVMEKL--------------
	Bacteroides plebeius	494838222	-------------Y----EQ	CHVDPATGRLV	P-PLEFAV-DAVMEKL--------------
	Parabacteroides johnsonii	495425753	-------------Y--T-EQ	CTIDPVTGKMV	P-PLAF-V-DAVMEKL--------------
	Bacteroides fragilis	492247874	-------------Y--TQEQ	CHIDPKTGRMV	P-PLAF-V-DGVM-EL--------------
	Prevotella saccharolytica	496453479	-------------Y--T-DQ	CTRDPKTGRLV	P-PLAF-V-DA-MEKL--------------
	Prevotella paludivivens	517276035	-------------Y----EQ	CTRDPKTGKLV	P-PLAF-V-DAVMEKL--------------
	Fibrobacter succinogenes	261417271	-------------Y----EQ	CTRN AQGKLV	P-PLAF-V-HA--QKL--------------
	Treponema brennaborense	332297587	------------RQ----EQ	CTRT ADGVLV	P-PLAF-V--A---KL--------------
	Megamonas hypermegale	479206841	-----A-------Y--T-DQ	CTRN EQGILV	P-PL-F-----VA--L-N-------A----
	Barnesiella intestinihominis	HAC12973	----------Y----EQ	CHVDPKTGRLV	P-PLAF-V-DAVMEKL--------------
	Phocaeicola coprophilus	204440964	-------------Y----EQ	CHVDPATGRLV	P-PLAF-V-DGVM-EL--------------
	Prevotella conceptionensis	044259939	-------------Y--T-EQ	CTRDPKTGRLV	P-PLAF-V-DA-M-KL--------------
	Pseudoprevotella muciniphila	111897780	-------------Y----EQ	CTRDPKTGKLV	P-PLAF-V-DA-MEKL--------------
	Parabacteroides distason	195473517	-------------Y--T-EQ	CTIDPVTGKMV	P-PLAF-V-DAVMEKL--------------
	Bacteroides faecichinchi	073349791	-------------Y--T-EQ	CHIDPVTGVMV	P-PLAFEV-DAVMEKL--------------
	Cand. Avibacter. excrementipullorum	HIS09802	-------------Y----EQ	CTVDPKTGRLV	P-PLAF-V-DGVM-EL--------------
	Coprobacter secundus	021930517	-------------Y----EQ	CTVDPKTGRLV	P-PLAF-V-DGVM-EL--------------
	Parabacteroides johnsonii	EEC95866	-------------Y--T-EQ	CTIDPVTGKMV	P-PLAF-V-DAVMEKL--------------
	Marseilla massiliensis	205111000	-------------Y----EQ	CTRDPKTGRLV	P-PLAF-V-D--MQKL--------------
	Cand. Prevotella stercoripullorum	HIW44521	-------------Y----EQ	CTRDPKTGRLV	P-PLAF-V-D--MQKL--------------
	Cand. Cryptobacter. intestinipullorum	HIS23577	-------------Y----EQ	CTLDPKTGRLV	P-PLAF-V-DGVM-EL--------------
	Prevotella buccae	048798599	-------------Y--T-EQ	CTRDPKTGRLV	P-PLAF-V-DA-MVQL--------------
	Brachyspira aalborgi	147526563	-------------Y-----Q		N--------K----K---------------
	Streptobacillus notomytis	172793479	-------------H-----D		---------K---E----------------
	Fusobacterium necrogenes	115269440	-------------H------		---L---------R-------------A--
	Sebaldella termitidis	012861482	-------------H-----D		N-----------------------------
	Caproicibacterium amylolyticum	212507242	-------------Y-----Q		---L-------VS—L----------ASSVP

Figure S9

Partial sequence alignment of the protein class II fructose-1,6-bisphosphate aldolase showing a 11 aa deletion (boxed) that is found in all members belonging to the order *Chlorobiales*

Group	Species	Accession	325	Insertion	365
Order *Chlorobiales*	*Chlorobaculum parvum*	193212482	SRSLMARLTPKEHRTEFFGFYD	G	SFGKASAVIGPLVFGFVS
	Chlorobium phaeovibrioides	145219537	---M------PS-V--------	-	T-------A------L--
	Chlorobium tepidum	21674159	-------------V--------	-	-------I-------V--
	Chlorobium ferrooxidans	493410723	---M------A--V--------	-	T------IA------L--
	Pelodictyon phaeoclathratiforme	194336684	---M-T----Q--V--------	-	T------IA------L--
	Prosthecochloris aestuarii	194334279	---M---------V--------	-	T------IL--VI--V--
	Chlorobium limicola	189347022	---M--K---R--VA-------	-	T------IV------M--
	Chlorobium phaeobacteroides	119357455	---M------Q--V--------	-	T------IA---L--M--
	Chloroherpeton thalassium	193213931	----------A--SA-------	-	-------IV--WI--LI-
	Chlorobium chlorochromatii	78188904	---M-T----Q--V--------	-	T------IV--FL--VI-
	Chlorobium luteolum	78187183	---M------RA-LA-------	-	T------IA------L--
	Prosthecochloris marina	PWW81998	---M------PK-V--------	-	T------IA--VI--VI-
	Prosthecochloris ethylica	194185816	--AM------PK-V--------	-	T------I---VF--MI-
	Chlorobaculum thiosulfatiphilum	139457788	-------------V--------	-	-------I-------I--
	Prosthecochloris vibrioformis	TNJ37359	---M-------K-V--------	-	T------IL--VL---I-
	Candidatus Chlorobium masyuteu	208019732	---M------A--V--------	-	T------IA------L--
	Chlorobium sp. N1	131353654	---M------RA-LA-------	-	T------IA------L--
	Chlorobium sp. KB01	076790259	---M------A--V--------	-	T------IA------L--
	Chlorobaculum sp. 24CR	129051526	---L---------V--------	-	-------I-------I--
	Prosthecochloris sp. HL-130-GSB	ARM31897	---M--K------V--------	-	T------IV------LI-
	Prosthecochloris sp. ZM	114617037	---M---------V--------	-	T------IL--VI--V--
	Prosthecochloris sp. CIB 2401	083188230	---M-------K-V--------	-	T------IL--VL---I-
	Prosthecochloris sp. GSB1	094082256	---M--K----A-V--------	-	T-------A--MI--LI-
Larger *Ignavibacteriae* Clade	*Chlorobi bacterium OLB7*	KXK57455	--T---L---ADKK--------	-	F------TL---IY-
	Ignavibacterium album JCM 1651	385810439	-----SK---P-KK-------S		F---S---L------L--
	Melioribacter roseus P3M-2	397689128	-----SK---YDKK-------S		F---S--IL------IT-
Other Bacteria	*Candidatus Kryptonia bacterium*	HHD65179	---L---V--AA-------	-	IA-N--------T--AI-
	Candidatus Kryptonium thompsonii	047133172	-----SKII-P-KK-------S		F---S--IL---------
	Weissella cibaria	498059571	---YLGQ-V---RSS-----FN		I---F--I---AI--I--
	Paenibacillus larvae	558474612	--T-IVQ-S-P-K-GQ---LFA		FS--V-SIV---LY-SIT
	Saccharibacillus kuerlensis	517806976	--TMIVE-S-P-K-GQ---LFA		FS--L-S-L----Y-S-T
	Listeria fleischmannii	494740195	---FYGKII---RSN--Y---N		I---F--I---ALL-VIT
	Lactobacillus oris	489811830	---YFGQ-I---ASS------N		IL--F--IL----V-L-T
	Bacillus thuringiensis	489348371	---YF-K-V---SAN------N		I---FA-IM--VLV-VTT
	Lactobacillus oris	489810428	---YFGQ-I---ASS------N		IL--F--IL----V-L-T
	Bacillus anthracis	497564380	---YF-K-V---SAN------N		I---FA-IM--VLV-VTT
	Bacillus cereus	488127310	---YF-K-V---SAN------N		I---FA-IM--VLV-VTT
	Weissella confusa	489703767	---YLGQII---RSS-----FN		I---F--I---AI--I--
	Paenibacillus mucilaginosus	337750739	--TMIVE---P-R-GQ---LFA		FS--V-SIV---LY-SIT
	Lactobacillus parabrevis	518933426	---YFG--V---RSS------N		IL--F---L--ILV-VIT
	Clostridium acetobutylicum	15894863	---YLGK-V--NKAN------N		I---F--IL--FMV-I--
	Caloramator australicus	496185404	---YFGK-V---NSN------N		I---FA-IM--FLV-L-A
	Nevskia ramosa	551356333	---YF---I-P-QAGQ-----N		ML--FA--L----V-I-A
	Marinitoga piezophila	374340297	----YGK-I---KSA------N		I---FA-I---FLV--F-
	Caldithrix abyssi	493986443	--A--GK-I-RGMEA--Y---A		LM--F-SIL--FT--L--
	Flexistipes sinusarabici	336324118	---FF---I-NDKSG------N		ML--FA--L--FMM-L-A
	Rhodospirillum centenum	209966532	A--M----S-P-MQ-----L-G		LT-R-IGF----AY-LAT
	Syntrophus aciditrophicus	85858063	---FFG--I---KSA------N		I---FATIL---LMALA-
	Desulfohalobium retbaense	258405958	----F--MV-E-KKA-----FS		ISA-FA-IV--F--AS-A
	Candidatus Methylomirabilis	392374612	--TF--S-I--GREG----C-A		LC--TASIL------A--
	Mariprofundus ferrooxydans	497536274	---MY--MI-SHQSA-----FN		ML--FA--L---MM-MA-
	Aminobacterium colombiense	294101413	----Y-S-L-PSRSA-------		ISS-FAG----AI--LIT
	Thermanaerovibrio acidaminovorans	269792021	----FS-IV---RSA-L-----		LSS-FAG-L--MA--VI-
	Thermanaerovibrio velox	493632134	----FS-IV--DRSA-L-----		LSS-FAG-L---A--VI-
	Sphaerochaeta globosa	325970677	---YYT--I--QRSA-----FN		ML--FA-I----LM-V-T
	Candidatus Cloacimonas	218961816	--TMLSL---RDRQA------T		LT-RL-SI---ILY-WIA
	Cand. Kryptobacter tengchongensis	CUS92867	-----SKII-P-KK-------S		F---S--IL------L--
	Cand. Thermokryptus mobilis	140944683	-----SKII-PSKK-------S		F---S--IL------T--
	Vagococcus salmoninarum	126781782	---YF--II---RSN------N		I---F---L---I--VMT
	Marivibrio halodurans	210683211	------HIA-P-L------L-A		LS---T-FM---IV-W-T
	Carnobacterium alterfunditum	143298371	----FGQ-I--Q-AN------N		I---FA--V---LV-II-
	Ferruginivarius sediminum	114580575	--------A-E-VK--M--L-A		LS--MT-F---AIV-W--
	Oenococcus kitaharae	OEY83698	---YFGK-L----AS-----FN		I---F--I---FL--
	Leuconostoc suionicum	211636353	---YFGQII--KYSG------N		I---F---L------S-A
	Xylanibacillus composti	213413321	---YF-KII----SN------N		I---FASIM---LV-
	Acidaminobacter hydrogenoformans	207646431	---YFGK-V---QAN------N		I---FA-IM--FLV---T
	Bacillus mesophilus	163180499	---YF-K-V--Q-SN------N		I---FA-IM---LV-VTA
	Psychrobacillus insolitus	111438262	---YFGK-V---NAN------N		I---FA-IM---IVA-T-

Figure S10

Partial sequence alignment of the protein major facilitator superfamily transporter, showing a 1 aa insertion (boxed) that is specific for members of the order *Chlorobiales*. Except for an isolated exception shown here, the CSI was not found in any other bacterial species in the top 500-BLASTp hits.

Group	Species	Accession	294	(insert)	340
Order *Chlorobiales*	*Chlorobium luteolum*	78187124	YLRHLTWEGAREKYG	DNPE	DGITPEDVKERIELELGVIEKMGFSSYF
	Prosthecochloris vibrioformis	139626774	------Y---KV--A	-PAS	Q-L-WQ--EA---R--D--TS-------
	Chlorobium phaeobacteroides	642923410	------Y---KK--A	-HDS	Q-LSW-KIE----K-----IS-------
	Chlorobium chlorochromatii	78188863	----------A---A	KSE-	E--SQ-E---------------------
	Chlorobium phaeobacteroides	119357011	----------K---A	-SGA	---SS---EA------------------
	Chlorobium limicola	189346342	----------A---A	GSEA	----E---RA------------------
	Chlorobium ferrooxidans	493409850	--S-------K---A	-SEA	E--SK-E--S------------------
	Chlorobium phaeovibrioides	145219820	------A---K---P	GIS-	-EPSQ-E-RD------------------
	Chlorobium phaeoclathratiforme	194336619	------L---K---A	-ADA	---SA---RA------------------
	Prosthecochloris vibrioformis	139626774	------Y---KV--A	-PAS	Q-L-WQ--EA---R--D--TS-------
	Prosthecochloris marina	110023626	------Y---K---A	-PES	Q-LSEKM-N----K--ET-SS-------
	Chloroherpeton thalassium	193215617	------Y---KK---	-LDA	M-EKGAE-R----Y--SI-C---YAT--
	Prosthecochloris aestuarii	194333751	------Y-------A	-PES	Q-L-WQQ-ED---K-----IS-------
	Chlorobaculum thiosulfatiphilum	TNJ36552	-----------N---	V-	--AI--E--A-----F-------Y-A--
	Chlorobaculum tepidum	21673669	----------K----	AA	--TI--E--A------------Y-A--
	Chlorobaculum limnaeum	2501894946	---------------	S-	--AV-D---A-V----------Y----
	Chlorobaculum parvum	193212527	-----------N---	V-	--AI--E---------------Y-A--
	Candidatus Chlorobium masyuteum	166808752	--S-------K---A	-SEA	E--SK-E--S------------------
	Cand. Thermochlorobacter aerophilum	RFM23112	---Y--YQ--LR---	NLDA	L-ERG-QIRN---Y--NT-A--------
	Chlorobium sp. N1	131353551	----------K----	Q--S	----K-----------------------
	Chlorobium sp. KB01	076790384	--S-------K---A	-SEA	E---K----A------------------
	Prosthecochloris sp. CIB 2401	068867018	------Y---KV--A	-PAS	Q-L-WQ--EA---R--D--TS-------
	Prosthecochloris sp. GSB1	094082691	------Y-------A	-PEA	G-L-WKM-E----K-----IS-------
	Prosthecochloris sp. HL-130-GSB	085659708	------Y----K--A	-PES	Q-L-WQQ-ED---K-----TS-------
	Prosthecochloris sp. ZM	167399480	------Y-------A	-PES	Q-L-WQQ-ED---K-----IS-------
Ignavibacteriae	*Melioribacter roseus P3M-2*	397689293	--TQ-AY--LHRRFK		KVTREIED-MKY--D--I---YAG--
Other Bacteria	*Porphyromonas asaccharolytica*	332299966	------Y---KKR--		DPV-PE-Q---DM-----QN---PG--
	Gracilimonas tropica	521069907	--K---Y----PR-K		EID-E-T--------I--E---AG--
	Porphyromonas endodontalis	490463816	--Q---Y---KMR--		DPI--E-----DF--NT-KS---PG--
	Dokdonia donghaensis	85818192	F----VY---KKR--		EELS-E-T--LDF--D----T-YPG--
	Capnocytophaga sputigena	488757420	------Y---KR--P		EITDE-R--LDF--SI---T-YPG--
	Capnocytophaga ochracea	488750278	------Y---KR--P		EITDE-R--LDF--SI---T-YPG--
	Niabella aurantiaca	517457778	------Y---QIR--		VM-DST----DF--FT-KT---AG--
	Chryseobacterium gleum	489070762	--T---Y---KNR--		EEGITDEI---LDF--E--ANT-YPG--
	Croceibacter atlanticus	298207508	F-K---Y---K-R--		EITPEI---LDF--SI---T-YPG--
	Kordia algicida	494155458	------Y---KKR--		EITPEIE--LDF---T--NT-YPG--
	Rhodothermus marinus	268317469	---D-VF----RR--		DPL-HE-A--L-H--AI-K----AG--
	Cecembia lonarensis	496475077	------Y---KKR-P		VITDEI---LDF--ET--RT-YPG--
	Porphyromonas catoniae	491609864	------Y---KR---		DELT-T-R---DF--ET-AN---PG--
	Empedobacter brevis	518818409	--K---Y---KKR-V		ELTDEI----DF--AT---T-YPG--
	Niabella soli	495857618	--K---Y---QKR--		SM-DST----DF--FT-KT---AG--
	Ornithobacterium rhinotracheale	392390690	------Y---KQR--		EITPEIT--LDF--AT--NT-YPG--
	Zobellia galactanivorans	340618163	----I-Y---KKR--		EITPEID--LDF--K----T-YPG--
	Bizionia argentinensis	495915021	F-----YM--KKR--		EELT-EIT--LDF--S---NT-YPG--
	Polaribacter franzmannii	517772471	F-K---FV--KKR--		EIT-SI---LDF--S----T-YPG--
	Microscilla marina	488781602	------Y---TKR--		EIT-EIR--LDH--NI-KS---PG--
	Chitinophaga pinensis	256423694	----I-M---HK--A		EVTAE-E---NF--S---N---AG--
	Galbibacter marinus	496279563	--K---YQ--KRR-A		EIT-EI---LDF--S---NT-YPG--
	Psychroflexus tropicus	517866879	--K---F---K-R--		ELS--I---L-F--S--ANT-YPG--
	Joostella marina	495886298	------Y---KRR-P		EIT-SI---LDF--S---NT-YPG--
	Lewinella cohaerens	522026781	------Y---KRR-D		TITPAI---LDF--K----S-YPG--
	Eudoraea adriatica	518500380	----I-Y---KKR--		EITDEIR---DF--KTV-NS-YPG--
	Elizabethkingia anophelis	496382600	F-----Y----K--E		EITDEIR--LDF--DI-A-T-YPG--
	Indibacter alkaliphilus	496327208	------Y---KKR-K		EITDEI---LDF--AT--NT-YPG--
	Elizabethkingia meningoseptica	544939559	F-----Y----K--E		EITDEIR--LDF--DI-A-T-YPG--
	Saprospira grandis	379731533	------Y---KRR--		SITPAI---LDF--E--RQS-YPG--
	Cyclobacterium marinum	343084733	F-----Y---KKR--		EITDEIR--LDF--ET--RT-YPG--
	Cellulophaga lytica	325286607	F-----Y---KIR--		EITPEID--LDF--K----T-YPG--
	Cesiribacter andamanensis	496488129	------Y----KR-P		AITPEIQ--LDF--ET-A-T-YPG--
	Porphyromonas gingivalis	34539911	---F--Y----R---		EDLSDEI----DF--ET-KG---PG--
	Planctomyces limnophilus	296121580	--Q----Q--EQR-P		HGL--KIRHLL-H--NL--ELRYEP--
	Rhodopirellula europaea	495942656	H-KR-------GRWP		-GV--K-I-TLRH-VTL--DLQYEA--
	Dictyoglomus turgidum	217967773	-FEK-C----KKRF-		ENI-QEI---LSY-IS--KQ----G--
	Patulibacter medicamentivorans	494846815	---AQVE--L-AR--		DPI-AAAR--A-M-----NR--YDA--

Figure S11

Partial sequence alignment of the protein DNA polymerase III subunit alpha, showing a 2-4 aa insert (boxed) that is specific for all members belonging to the order *Chlorobiales*

Group	Species	Accession	103		137
Order *Chlorobiales*	*Chlorobium phaeobacteroides*	119356086	DDKAIVGGFAILED	IPSGFS	QPVMIIGHQKGRDTK
	Chlorobium ferrooxidans	493410149	----------RI--	SA----	---------------
	Chlorobium luteolum	78187798	----------R---	RA--Y-	----V----------
	Chlorobium phaeovibrioides	145220402	----------RI--	RV----	----V----------
	Chlorobium phaeoclathratiforme	194337654	-----I----RI-E	SAT---	---------------
	Chlorobaculum tepidum	21673002	-----I----R---	ESA---	-S--V----------
	Chlorobium limicola	189345751	----------R--E	SA----	-T--M----------
	Chlorobium chlorochromatii	78188341	-----I----R---	EAA---	-S--M----------
	Chlorobaculum parvum	193213516	-----I--L-R---	KD----	-S--V----------
	Prosthecochloris aestuarii	194333138	----------RI--	SEAS--	-T--V----------
	Chlorobium phaeobacteroides	189499305	----LI----RI--	EERD--	-T--V----------
	Chloroherpeton thalassium	193215260	----------K-T-	DEQR--	KT-------------
	Chlorobaculum limnaeum	2501894381	N----I----RI--	EAT---	-S--V----------
	Prosthecochloris marina	110022889	----------RI-E	EE---K	-T--V----------
	Chlorobaculum thiosulfatiphilum	139457840	-----I----R-A-	EAT---	-S--V----------
	Candidatus Chlorobium masyuteum	166807605	----------RI-E	PST---	---------------
	Candidatus Thermochlorobacteri GBChlB	KER09150	-----I----K-S-	GQ---	-G-AV----------
	Cand. Thermochlorobacter aerophilum	RFM23425	----------RMI-	EDGRA	-T-------------
	Chlorobium sp. N1	131354938	----------R---	RA--Y-	----V----------
	Chlorobium sp. 445	PI048713	--------L-RMV-	DDHH--	-T-------------
	Chlorobium sp. KB01	076789707	----------RI--	SA----	R--------------
	Chlorobaculum sp. 24CR	129050980	-----I----R---	EAT---	-S--V---------R
	Prosthecochloris sp. HL-130-GSB	085658914	----------R---	RDLS--	RT--V----------
	Prosthecochloris sp. GSB1	094081818	----------TIG-	EADD-F	-T--V----------
Ignavibacteriae	*Ignavibacterium album*	78187798	--------L-Q-GN		HK-V-M-Q-------
	Melioribacter roseus	397689297	----------K-DG		-K-------------
Other Bacteria	*Glaciecola polaris*	494165670	-----I--L-L-DE		--I-V----------
	Salinimonas chungwhensis	517813267	-----I--L-M-D-		----L---------
	Glaciecola chathamensis	495264166	-----I--L-M-DE		--I-V----------
	Pseudoalteromonas atlantica	109897590	-----I--L-M-DE		--I-V----------
	Erwinia piriflorinigrans	562748821	--------I-R---		R-----------E--
	Morganella morganii	571171262	--------L-R--G		R---V----------
	Erwinia pyrifoliae	259907558	--------I-R---		R-----------E--
	Vibrio metschnikovii	490529482	--------I-R-D-		------------E-R
	Erwinia amylovora	312173371	--------I-R---		R-----------E--
	Providencia sneebia	496196770	--------I-R--G		----V-------E--
	Alteromonas macleodii	406595876	--------P-M-D-		--------------N
	Dickeya zeae	515508915	--------I-R-D-		R-----------E--
	Glaciecola arctica	494895163	--------L-KIDG		---------------
	Dickeya dadantii	271499511	--------I-R-D-		R-----------E--
	Nitrococcus mobilis	491140726	--P-----V-R--G		----V----------
	Salmonella enterica	487762226	--------I-R--G		R-----------E--
	Yersinia mollaretii	491012799	--------I-R--G		R-----------E--
	Shigella flexneri	491248512	--------I-R-DG		R-----------E--
	Yersinia frederiksenii	490844621	--------I-R-DG		R--------------
	Yersinia ruckeri	490857600	--------I-R--G		R-----------E--
	Geobacter daltonii	222056374	--H-----L-R-DG		E---V----------
	Geobacter uraniireducens	148264551	--H-----L-R-DG		V---V----------
	Solitalea canadensis	387792181	----------TIDG		----------KN--
	Thermotomaculum hydrothermale	201328645	----------F-D-		I--C---Q-------
	Alteromonas macleodii	HBI76693	--------P-M-D-		--------------N
	Salinimonas lutimaris	137167805	-----L--L-M-D-		--I-L---------
	Morganella morganii	HBH7053723	-----I--L-R--G		R---V----------
	Vibrio metschnikovii	217523606	--------I-R-D-		------------E-R
	Bowmanella denitrificans	102796617	--------L-R-D-		--------------
	Klebsiella pneumoniae	NGE24729	--------L-R--G		R---V----------
	Providencia sneebia	008916607	--------I-R--G		----V-------E--

Figure S12

Partial sequence alignment of the protein acetyl-CoA carboxylase carboxyltransferase subunit alpha, showing a 6 aa insertion (boxed) that is found in all members belonging to the order *Chlorobiales*

Group	Species	Accession	88	Insertion	132
**Order *Chlorobiales***	*Chloroherpeton thalassium*	193214252	LHVHYAIPHAMSALLAKEMLKEKEDRH	QNF	KLVTTLHGTDITIVG
	Chlorobium phaeoclathratiforme	194335958	V-A-------L--M--RQ-IED-CSEL	KC-	-----------V--
	Chlorobium phaeobacteroides	189500750	V-A-------L--M--RQ--EDSCSEA	KC-	--A---------V--
	Chlorobaculum tepidum	21673383	V-A-------I--M--RQ--E--CPAA	EC-	RI-------------
	Chlorobium luteolum	78186436	V-A---------M--RQ--EDRCPDA	SC-	R-A---------V--
	Chlorobaculum parvum	193213088	V-A-------I--V--RQ--E-RCPES	-C-	RIA------------
	Chlorobium ferrooxidans	493410448	V-A---------M--RQ--ED-CCGS	NC-	-IA---------V--
	Chlorobium limicola	189346053	V-A-------L--M--RQ--ED-CAEA	RC-	R-A---------V--
	Chlorobium chlorochromatii	78189259	V-A-------L--M--LQ--ED-CAAA	HC-	------------V--
	Chlorobium phaeovibrioides	145219409	V-A---------M--RQ--ED-CPQR	DC-	R-A---------V--
	Prosthecochloris marina	110023977	V-A-------L--M--RQ--E-SCSDA	RC-	R-A---------V--
	Prosthecochloris aestuarii	194333524	V-A-------L--I--RQ-VED-CSKA	-C-	RIA---------V--
	Chlorobaculum limnaeum	2501893378	V-A-------I--M--RQ--E--CPAA	EC-	RI-------------
	Chlorobaculum thiosulfatiphilum	139456871	V-A-------I--M--RQ--E--CPAS	EC-	RI-------------
	Candidatus Chlorobium masyuteum	166808429	V-A---------M--RQ--ED-CSSS	KC-	R-A---------V--
	Chlorobium sp. N1	131358741	V-A---------M--RQ--ED-CPER	GC-	R-A---------V--
	Chlorobium sp. KB01	076792893	V-A---------M--RQ--ED-CCGS	NC-	-IA---------V--
	Prosthecochloris sp. ZM	114617556	V-A-------L--I--RQ-VED-CSKA	-C-	RIA---------V--
	Prosthecochloris sp. GSB1	094082046	V-A-------L--I--RQ--ED-CSES	RC-	R-A---------V--
	Chlorobaculum sp. 24CR	RXK88293	V-A-------I--M--RQ--E--CPAS	EC-	RIA------------
	Prosthecochloris sp. HL-130-GSB	085659945	V-A-------L--M--RQ--ADTCRDA	DC-	RIA---------V--
**Larger *Ignavibacteriae* Clade**	*Chlorobi bacterium NICIL-2*	KXB98016	----------V-GY---QV-ARDGIQL		RV----------L--
	Chlorobi bacterium OLB6	KXK33709	V---------V-GF--SLI--D-YPF		--L---------L--
	Chlorobi bacterium OLB7	KXK52241	----------S--YM--LI-N-QGRDI		-II---------LI-
	Melioribacter roseus	397689377	M---------V--Y---QIR-KSNGDI		-FI---------LM-
	Ignavibacterium album	385811286	V---------T--Y------ KGYCDL		-II---------L--
**Other Bacteria**	*Runella slithyformis*	338212610	----------S--Y---QI-SQGIHI		PFI---------L--
	Marivirga tractuosa	313677397	----------SA-YM--QI----NINI		PV----------L--
	Leadbetterella byssophila	312129374	----------T--F---QI-QQEGLDI		PVI------------
	Chitinophaga pinensis	256420235	----------ST-Y---QIVSKQGRIV		PFI---------L--
	Nafulsella turpanensis	516341204	----------SA-YM--QI--TQGIDI		PV----------L--
	Flexithrix dorotheae	522020948	----------SA-YM--QI--T-GINI		PF----------L--
	Ornithobacterium rhinotracheale	392389713	----------YA-YI--QI---RGKDL		PII---------L--
	Echinicola vietnamensis	431796158	----------SA-YM--QI--TQGIEI		PV----------L--
	Flexibacter litoralis	392399217	----------S--YM--QI--TEGIQI		PV----------L--
	Sphingobacterium paucimobilis	544633139	----------SA-Y---QI-RSYGIDI		PV----------L--
	Algoriphagus machipongonensis	495478511	----------SA-YM--QI---EGIQI		PV----------L--
	Fulvivirga imtechensis	497269414	----------SA-YM--QI--TEGINI		PV----------L--
	Cesiribacter andamanensis	496488412	----------SA-YM--QI--SQGISI		PV----------L--
	Emticicia oligotrophica	408674449	----------T--F---QI--CHGINI		PII------------
	Cyclobacterium marinum	343085183	----------SA-YM--QI--KQGVII		PV----------L--
	Cyclobacterium qasimii	527057196	----------SA-YM--QI--KQGIVI		PV----------L--
	Microscilla marina	488785813	----------SA-YM--QI--SEGISI		PVI---------L--
	Capnocytophaga granulosa	512456577	I-A-------YT-YM--QI-EKEGLDV		-II---------L--
	Fluviicola taffensis	327405499	----------SA-FM-QQI--AQGINI		PFI---------L--
	Mariniradius saccharolyticus	495902631	----------SA-YM--QI---EGIHI		PV----------L--
	Niabella aurantiaca	517456348	----------SA-YM--KI-EKEGKHI		PVI---------L--
	Rhodonellum psychrophilum	518426151	----------SA-YM--QI---EGIHI		PV----------L--
	Bacillus selenitireducens	297584395	----------I--Y---Q-V-KDV		-II---------VL-
	Terriglobus roseus	390959900	---------SV-----RQ--EA-GI-L		PFI---------L--
	Acidobacterium capsulatum	225872421	----------I--F--R---AKRGLHI		PI----------L--
	Thermincola potens	296133833	----------V--Y---Q---GELEL		PVI------------
	Lutibacter agarilyticus	089380012	----------YA-YM--Q-----GIDI		-V----------L--
	Acidipila dinghuensis	129208410	---------SI--Y--RQ--A---RRL		PF----------L--
	Aureivirga marina	196892692	----------YA-YM--Q-----GIDI		-VI---------L--
	Polaribacter sejongensis	208889400	----------YA-YM--Q-----GLDV		RV----------L--
	Marivirga tractuosa	013455427	----------SA-YM--QI----NIKI		PV----------L--
	Flavicella marina	152286514	----------YA-FM--Q----QGIDI		-V----------L--

Figure S13

Partial sequence alignment of the protein N-acetyl-alpha-D-glucosaminyl L-malate synthase BshA, showing a 3 aa insertion (boxed) that is found in all members belonging to the order *Chlorobiales*.

Group	Organism	Accession	228	Insert (boxed)	278
**Order *Chlorobiales***	*Chlorobium limicola*	WP_041466021	`KTLRGPRGGMIMMGSDFENPLGI`	`TI`	`KTKTGSRVKMMSEVMDAEVMPGIQGG`
	Chlorobium luteolum	WP_011358316	`--------------K-----M--`	`--`	`---N-P--------------------`
	Candidatus Chlorobium masyuteu	WP_166806951	`--------------------M--`	`--`	`--------------------------`
	Chlorobium sp. KB01.	WP_076791726	`--------------------M--`	`--`	`--------------------------`
	Chlorobium sp. N1	WP_131354081	`--------------R-----M--`	`-V`	`---N-P--------------------`
	Chlorobium phaeobacteroides	WP_011744490	`-----------------------`	`--`	`-----------T--------------`
	Chlorobium phaeovibrioides	WP_011890615	`--------------K-----M--`	`--`	`---N-Q--------------------`
	Pelodictyon phaeoclathratiforme	WP_012507517	`--------------------M--`	`--`	`-------L------------------`
	Chlorobium ferrooxidans	WP_006365450	`--------------------M--`	`--`	`--------------------------`
	Chlorobaculum tepidum	WP_010933254	`--------------K-------L`	`--`	`N--N----------I-----------`
	Chlorobaculum thiosulfatiphilum	WP_139456325	`--------------K-------L`	`--`	`N--N-P--------------------`
	Chlorobaculum parvum	WP_012502749	`--------------K-----M--`	`-V`	`N--N-P--------I-----------`
	Chlorobaculum limnaeum	WP_069808880	`-------------DK-------L`	`--`	`N--N-P--------------------`
	Prosthecochloris ethylica	WP_114608737	`--------------Q-----M--`	`--`	`-----R-T------I---I-------`
	Prosthecochloris vibrioformis	WP_139626252	`--------------K--------`	`-V`	`---N-P-T------I---I-------`
	Prosthecochloris aestuarii	WP_012505177	`--------------E-----M--`	`--`	`---A-R-T------I---I-------`
	Prosthecochloris marina	WP_110024280	`--------------Q--------`	`-V`	`---K-V-T------I---I-------`
	Chloroherpeton thalassium	WP_012500494	`-----------L--K-----M--`	`KA`	`-----E-I--V--LL--M--------`
	Cand Thermochlorobacteriacea GBChlB	KER09762.1	`-----------L--K-----F--`	`KL`	`-----E-L-L----L-T-----V---`
	Chlorobium sp. 445	PI047396.1	`-----------L--K-----F--`	`KV`	`-----E-L------L-T-----V---`
**Larger *Ignavibacteriae* Clade**	*Chlorobi bacterium OLB7*	KXK56915.1	`---------V-L--K-----F--`		`VAPKSG-T-----II-SW--------`
	Chlorobi bacterium OLB4	KXK04106.1	`---------I-L--K----TF--`		`VA-S- -T-----LI-SM--------`
	Chlorobi bacterium OLB5	KXK53076.1	`  ---------L--A-----F-L`		`TAPKSG-------LI-SM-I--V---`
	Chlorobi bacterium OLB6	KXK35191.1	`---------L-LL-K-V---F-V`		`AP-S- -------LI-SM--------`
	Ignavibacteriales bacterium UTCHB3	OQY75246.1	`---------V-L--K-----F--`		`TVPKTG-A-----LI-STI-------`
	Melioribacter roseus	WP_014854729	`---------V-LI-K-K---M--`		`--VK-D-L-L---IF-GM--------`
	Ignavibacterium album	WP_014560320	`---------I-LI-K-K---W-L`		`T-PK-D-------LI-GM--------`
**Other Bacteria**	*Cand. Kryptonium thompsoni*	WP_047133370	`---------L-L-HK-R---F--`		`--PK-D-LR----II-GT--------`
	Cand. Kapabacteria thiocyanatum	OJX61272.1	`---------L-I--KNY---F-V`		`AP-S- -T-L-G-LI-SM--------`
	Rhodothermus marinus	WP_012842647	`-----------LI-R--D--F--`		`TA-S- -I-K---LL-SA-F--T---`
	Bacteroides fragilis	WP_008768732	`---------V----K--P--W-K`		` KPK- EI----QLL-SA-F------`
	Phocaeicola massiliensis	WP_117462813	`---------V----K--P--W-K`		` -PK- EI----QLL-SA-F------`
	Salinibacter ruber	WP_118838368	`  ---------LL-D-Y---M-K`		`TARKSG-T-----LL-SA-F--T---`
	Cytophaga aurnatiaca	WP_018341732	`---------V----K-----F-L`		` -PK-E -R---N-L-MG-F--T---`
	Sinomicrobium oceani	WP_072318454	`--------------K-----F--`		` -LKNGKPR---SLL-SA-F--N---`
	Thermaurantimonas aggregans	WP_124398693	`---------L-L--K-----F-K`		` -PK- EIR---SLI--A-F--V---`
	Seonamhaeicola marinus	WP_148541715	`--------------Q-----F--`		` -LKSGKLRK--SLL-SA-F--N---`
	Schleiferia thermophila	WP_037361247	`---------L-L--N-----F-K`		` -PK- EIR---SLI--A-F--V---`
	Snuella sedimenti	WP_199115674	`--------------E--D--F--`		` -LKSGKLRK--SLL-SA-F--N---`
	Tamlana sedimentorum	WP_044632150	`--------------K-----F--`		` -LKNGSLRK--SLL-SA-F--N---`
	Flavobacterium koreense	AFR46549	`--------------K-----W-L`		` -PK- EIR---H-L-MS-F--N---`
	Seonamhaeila aphaedonensis	WP_116039982	`---------L----K-----F--`		` -LKSGKLRK--SLL-SA-F--N---`

Figure S14

Partial sequence alignment of the protein Serine hydroxymethyltransferase, showing a 2 aa insertion (boxed) that is found in all members belonging to the order *Chlorobiales*

Group	Organism	Accession	94	Insertion	144
**Order *Chlorobiales***	*Chloroherpeton thalassium*	193215546	ALNGAPGVYSARYAATPQKEKPS	YAD	NVDKLLSDMQNLTNRTARFRTVIAL
	Chlorobium phaeobacteroides	189500796	G-----------F-PV-EGTS-T	-D-	--NH--KE-E-V-D-K-T----V--
	Chlorobium ferrooxidans	493409268	--G---------F-PM-EGKS--	-Q-	--RH--AS-AGCS--K-L-------
	Prosthecochloris aestuarii	194334496	--K---------F-PM-EGRE--	-E-	--QH--TT--HTLE-S-T------I
	Chlorobium phaeovibrioides	145219308	--G-----------PVANG---T	-R-	--TH--NE-EGEKD-S---------
	Chlorobium phaeoclathratiforme	194337267	G-D---------F-PV-DGQS-T	-K-	--AH--HC-KGIA--E---------
	Chlorobaculum parvum	193213344	----E-------F-PM-PGQS-T	---	--RH--DR-TGK-D-S--------M
	Chlorobaculum tepidum	21673169	--G-----------PV-EGVART	-E-	--RH---E-RGKSK----------M
	Chlorobium chlorochromatii	78188461	--D-----------P-ADGTA-T	-S-	--NH--KN-AGKEE-S-C--SL---
	Chlorobium luteolum	78186333	Q-G-R-------F-PR-EG----	-S-	--RH--QE-EGKVE-S---------
	Chlorobium limicola	189347225	S-G-E---L---F-PVKTGM--T	-E-	-I-H--LR-NGIKE-N---------
	Prosthecochloris ethylica	175187634	--D---------F-PM-EGQL-A	-E-	--RH--KV--HHS--N-T------I
	Prosthecochloris marina	110024145	G-----------F-PM-GGKP-T	-D-	--NH--KM-ESI-D-K-L-------
	Chlorobaculum thiosulfatiphilum	139457588	--G-----------PV-EGVART	---	--RH---E-RGKSE----------M
	Candidatus Chlorobium masyuteum	166807860	--D---------F-PM-EGKS--	-Q-	--RH--AN-AGCS--N-Q-------
	Cand. Thermochlorobacter aerophilum	RFM23469	--G-E---H--H---AELGRQ-T	-SE	--A---RK---IRQ-Q-V----MAL
	Cand. Thermochlorobacteriaceae GBChlB	KER10877	--G-----------TAELGR--T	---	--E---R-LSGASS-A------LS-
	Prosthecochloris sp. ZM	114616877	--K---------F-PM-EGRE--	-E-	--QH--TT--HTLE-S-T------I
	Chlorobaculum sp. 24CR	129051383	--G-----------PV-EGATRT	---	--RH---E-RGKSE----------M
	Chlorobium sp. KB01	076792242	—G---------F-PM-EGKS--	-Q-	--RH--AS-AGCS--K-L-------
	Chlorobium sp. 445	PI048559	--G-----H-----ERELGR---	-SE	--A---RELKGV-H-E-K-------
	Prosthecochloris sp. HL-130-GSB	085660539	------------F-PM-EGTS-T	-E-	--RH--GT-KGHSD-N-T------M
	Prosthecochloris sp. GSB1	094082037	--D-----------PVDNERS-T	-E-	--RH--AM-R-ER--E-S------M
**Larger *Ignavibacteriae* Clade**	*Chlorobi bacterium OLB7*	KXK51637	--E-----------GPDATYAD		--CR--IDTLADQE--R-Q---LC
	Ignavibacterium album	385810929	Q---E--I------GESADDSL		-NE--IKAI-KFPEPHRAKFVCV-S
**Other Bacteria**	*Bacteroides stercoris*	491891338	--------------GDAHNSEA		-MQ---Q--EGME--K------F--
	Bacteroides clarus	496413113	--------------GDAHNSEA		-MK---Q--EGIE--K-Q----F--
	Bacteroides gallinarum	517495029	--G-----------GNAHDSEA		-MK---Q--EGIE--K------F--
	Porphyromonas levii	517170024	--G-----------GPKCNP-D		-IE---GEL-GI---K---------
	Bacteroides massiliensis	492742940	----E---------GGEGHNAE	A	-MQ---QN--GVQ--K-Q-----C-
	Owenweeksia hongkongensis	375012035	--------------GEGANSEN		-M----TELDGKE--K-Q------Y
	Echinicola vietnamensis	431796557	--D-------G---GA-RSDDR		------EN-EGV-D-K-Q-------
	Gracilimonas tropica	521070784	--D-----F-----GEDASY-D		-LE----E-EGK-V-S-Q----V--
	Parabacteroides distasonis	492483186	V--N----------G-GHDSEA		-MN----E-NHKE--K---------
	Prevotella maculosa	518812447	--G-----------EQDDHNSE	A	-MA---HN-ADI-C-N-------S-
	Porphyromonas somerae	516661827	----R---------GLECRAED		-IQ---GEL-GIQ--S------V--
	Emticicia oligotrophica	408673533	----E---F-----GEHGNHAK		-IE---QTLEGVE--G-Q--S--T-
	Prevotella veroralis	490517531	----E---H-----EGTDHDSE	A	-MR----K-S-VKD---------S-
	Cecembia lonarensis	496476136	--D-------G---GE-RSDER		-IN---KE-EGK---K---------
	Parabacteroides goldsteinii	497546258	---N----------GPGHDSEA		-MK---RE-EGME--K---------
	Photobacterium angustum	491513700	Y------I----F-GEGASDAD		-I----AE-KDVPAEQRTA-FHCV-
	Pseudoalteromonas spongiae	498247569	--G----I----F-GVNANDSD		-I-----ELGDNP------WC-LV-
	Vibrio cholerae	446647661	Y------I------GEHASDGD		-LN---AA---VP-DQRSA-FHCV-
	Staphylococcus warneri	488381256	----E---------GLDKNDQD		-IN---KN-E-ISD-N-Q-VC--SM
	Staphylococcus epidermidis	488407224	----E---------GLGKNDED		-IE---TNLEDVQD-R-Q-VC--SM
	Prevotella bergensis	494310511	--G-----H-----EGTDHDSE	A	-MA---HELEGKE----------S-
	Echinicola vietnamensis	041739508	--D-------G---GA-RSDD	R	------EN-EGV-D-K-Q-------
	Flavobacterium cerinum	128389128	----E---------GEQKSAE		--M----KALEDS---N---K-----
	Enterococcus faecalis	RXF53884	--G-----------GENKSFD		--IE---RE-NGAND-N---------
	Niabella yanshanensis	114790679	----E---K-----DNEPEFA		--I----NK-K-QG--N-------S-
	Hymenobacter busanensis	151078078	--------------GPQRDAA		--IQ---LEL--Q-D-A-Q----V--
	Porphyromonas levii	134849553	--G-----------GPKCNP-		--IE---GEL-GI---K---------
	Chitinophaga silvisoli	116851431	--------------GEHKSSE		--I--A--E-KGK-D-R-Q-----S-

Figure S15

Partial sequence alignment of the protein deoxyribonucleotide triphosphate pyrophosphatase, showing a 3 aa insertion (boxed) that is found in all members belonging to the order *Chlorobiales*

Group	Species	ID	37		69
Order *Chlorobiales*	*Chlorobium limicola*	189346136	LGDTMVLATVVSSKKTPP	P	NQDYFPLQVEYREK
	Chlorobium phaeoclathratiforme	194335788	Q-----I-----------	-	--------------
	Chlorobium ferrooxidans	493409513	------------------	-	--------------
	Chlorobium chlorochromatii	78189646	------------------	L	G--F----------
	Chlorobium phaeobacteroides	119356388	--------------TP--	A	--------------
	Chlorobium phaeovibrioides	145220228	M-----I----------S	-	---F----------
	Chlorobaculum parvum	193212209	-N------------TP--	-	S--F----------
	Prosthecochloris aestuarii	194333349	-N------------TP-S	-	--SF----------
	Chlorobium luteolum	78187489	M-----I------R---S	V	---F----------
	Chlorobaculum tepidum	21674467	MN------------TP-S	-	---F----------
	Chlorobium phaeobacteroides	189499525	M-----I------RTP-S	-	---F--M-------
	Chloroherpeton thalassium	193214790	-EE---------K-DA--	A	--NF----------
	Chlorobaculum limnaeum	2501894253	MN----------TRTP-S	-	---F----------
	Chlorobaculum thiosulfatiphilum	139456275	------------T-TP--	L	---F--M-------
	Prosthecochloris marina	110023770	M-------------TP--	-	---F----------
	Cand. Thermochlorobacter aerophilum	RFM23016	-AE--------ARKDD-V	—	G--FI-----F---
	Candidatus Chlorobium masyuteum	166806995	------------------	-	--------------
	Chlorobium sp. N1	131354161	M-----I------R-A-S	V	---F----------
	Chlorobium sp. KB01	076791792	------------------	-	--------------
	Chlorobaculum sp. 24CR	129050239	-N-------------P-S	-	---F----------
	Chlorobium sp. N1	131354161	M-----I------R-A-S	V	---F----------
	Prosthecochloris sp. ZM	114618152	-N------------TP-S	-	--SF----------
Larger *Ignavibacteriae* Clade	*Chlorobi bacterium OLB5*	KXK53677	---I--C---GADE--K	E	GM--------F---
	Chlorobi bacterium OLB4	KXK02521	V--NV--C-A-TA-EE-A	—	G--F---S-D----
	Chlorobi bacterium OLB7	KXK53815	-------VA-AAG---R	E	GI-FL-----F---
	Ignavibacterium album	385809228	Y------V-A-A-EDVKD		D--F----------
	Melioribacter roseus	397690716	Y------V-A-AGE VRD		DI-F---S------
Other Bacteria	*Marinimicrobia bacterium*	551209032	Y------V-A-ANYFSKS		E--F---------R
	Kiloniella laminariae	522079354	Y---V--C-A-GARDVK-		G--F---T-N-Q--
	Magnetococcus marinus	117926996	Y---V--V-A-AETSMRA		G--F---S-H-Q--
	Acetobacter pasteurianus	517918340	Y-E-V--C-A-GA-DVK-		G--F---T-N-Q--
	Gluconobacter oxydans	414341474	Y---V--C-A-GA-SVK-		G--F---T-N-Q--
	Oceanicaulis alexandrii	550952395	Y-E-T----A-AQ-DVK-		GM-F---T-N-Q--
	Gluconobacter frateurii	565862949	Y---V--C-A-GA-SVK-		G--F--MT-N-Q--
	Gluconobacter thailandicus	494494374	Y---V--C-A-GA-SVK-		G--F--MT-N-Q--
	Acetobacter pomorum	493059067	Y-E-V--C-A-GA-EVK-		G--F---T-N-Q--
	Saccharibacter floricola	517809778	Y-E-V--C-A-GA-TVKA		G--F---T-N-Q--
	Gluconacetobacter hansenii	489712894	Y---V--C-A-GA-SVK-		G--F---T-N-Q--
	Gluconacetobacter diazotrophic	162147166	Y---V--C-A-GARSVK-		G--F---T-N-Q--
	Elioraea tepidiphila	517846087	Y---V------GA-T-K-		G--F---S-H-Q--
	Bartonella schoenbuchensis	498389360	Y-E-I-------A-NPK-		D--F---T-N-Q--
	alpha proteobacterium	496820156	W---VIIS-A-A----N-		DT-F---T-N-Q--
	Xanthobacteraceae	517199826	Y---T----A--A-SAK-		G--F---T-N-Q--
	Bartonella bovis	498386729	Y-E-I-------A-NPK-		D--F---T-N-Q--
	Acetobacter tropicalis	493605112	Y-E-V--C-A-GA-NVKA		G--F---T-N-Q--
	Azospirillum lipoferum	374293408	Y---T--C-A-AA-APK-		GV-F---T-N-Q--
	Bartonella melophagi	494740673	Y-E-I-------A-NPK-		D--F---T-N-Q--
	Azospirillum amazonense	490393941	Y---V--C-A-GA-SVK-		G--F---T-N-Q--
	Agrobacterium tumefaciens	523694192	Y-E---------A-SPK-		G--F---T-N-Q--
	Methylovorus glucosetrophus	253997787	Y---V--V---GKTDVK-		G--F---T-D-Q-R
	Methylobacillus flagellatus	91774427	Y---V--VS--GKREVKE		G--F---T-D-Q--
	Thiobacillus denitrificans	74316711	MD-----V---AKNEVK-		G--F---T-D-Q--
	Leeia oryzae	516890550	M---V--VS--AATNVK-		G--F---T-D-Q-R
	Selenomonas bovis	518371421	Y---V--V-ATA--EPRE		GV-F---T-D-E--
	Mitsuokella multacida	492423460	Y---V--V-ATA-AEPRE		GV-F---T-D-E--
	Selenomonas flueggei	493740328	Y---V--V-ATA--APRE		GV-F---T-D-E--
	Caldithrix abyssi	493987866	Y------V---ANDEMTE		QR-FM--T------
	Rhodothermus marinus	268316873	Q------C-A-LADEPRE		G-SF---T------
	Enterococcus faecalis	514895238	Y---V--SAA-A--EAKD		T-F---TIN-E--
	Haemophilus influenzae	270315521	MD--T-FV---AK-DVKE		G--F---T-N-Q-R
	Pasteurella multocida	401694220	MD--T-FV---AK-DVKE		G--F---T-N-Q-R
	Megamonas funiformis	547819988	Y-E-V--V-ATA-AEPRE		GV-F---T-D-E--
	Acidithiobacillus ferrivorans	344200724	S---V--V-A-ARREMK-		G--F---T-N-Q--
	Stenotrophomonas maltophilia	491555837	M---V--V-A-G--RAKD		GL-F---T-----R
	Fangia hongkongensis	517111971	A---V--V-A-AK-EVKD		GA-F---T-N-Q--
	Roseiflexus castenholzii	156743526	Y----L---A-A--EPRT		DT-F---T-D-E--
	Thermovibrio ammonificans	319789227	Q---I--V-A-M-DEPRE		DV-F---L-----R

Figure S16

Partial sequence alignment of the protein polynucleotide phosphorylase/polyadenylase, showing a 1 aa insertion (boxed) that is found in all members belonging to the order *Chlorobiales*

Group	Species	ID	264	Insertion (boxed)	335
Order *Chlorobiales*	*Chlorobaculum parvum*	193212209	KEERSDRTKAIYKETIAKTLEHFSS	ITAEEIAANPEKALCQS	EHMIDECIHDVEKKVMRHMILDDSKRLDGR
	Chloroherpeton thalassium	193214790	----A-K-AEV-ADAS-AV-RKYKAE	--D-IL-E-----IYLN	-KA-NDY---I--H---E---A-A------
	Prosthecochloris aestuarii	194333349	----AEK-AE---NAK-Q--QRYQQE	--P-V---E-----YLN	-QI-GDA--SI--Q---E-----A------
	Chlorobium phaeobacteroides	189499525	----AEK-T-V--SITEQI-GRYKNE	----D---D-S---YLN	-QI-SGH--AI--Q---------A------
	Chlorobium phaeoclathratiforme	194335788	----A-Q-A---H-I-ES-I---KAT	CSS--FSTD-A----IN	P-I---Q--M---R---------A------
	Chlorobium luteolum	78187489	--V-A-E-----S--VK-AI----AA	FGP-AL--D-S----LN	--I-E-E--SF--N---R-----G------
	Chlorobium phaeovibrioides	145220228	----AE---E--G--LKSAV----AA	FSS-DL--D-S----TN	--I-E-E--S---D---R-----A------
	Chlorobium chlorochromatii	78189646	----AE--A---N--L-A-----RAT	-SS--AQ-DTA----LN	--I-EDQ--A---E---------A------
	Chlorobium ferrooxidans	493409513	--D-A-Q-AH--R---QAIV-K-K-S	-SS-D---D-A----LN	--I-E-E--S--------------------
	Chlorobium limicola	189346136	--D-AEQ-A---R-I-QS-V---KAA	-SP---Q-D-S----LN	--I---Q--A-------------A------
	Chlorobaculum tepidum	21674467	----AEK-----TQ--R---T--TDR	VGPDQ-E-D-TS-F-LN	----E----A-------------G------
	Chlorobaculum limnaeum	2501894253	----AEK-----TQ-LR---T--TDR	VGPDQ-E-D-SS-F-LN	----E----S--------------------
	Chlorobaculum thiosulfatiphilum	139456275	----AEK-----TQ-LR---T--TDR	VGPDQ-E-D-SS-F-LN	----E----A--------------------
	Candidatus Chlorobium masyuteum	166806995	--D-A-Q-AH--R---QAIV-KFK--	-SS-D-----A----LN	--I-E-E--S--------------------
	Cand. Thermochlorobacter aerophilum	RFM23016	-DV-AEK-----D--L-AV--KYKAE	--P-VL--S-----YLN	-KL-RQV---C-RDA--QT--N-RV-----
	Chlorobium sp. N1	131354161	----A-E-----G--VQQ-I----AA	L----L--DSS----LN	--I-E-E--SI------R-----G------
	Chlorobium sp. KBO1	076791792	—D-A-Q-AH--R---QTIV-KF---	-SS-D---D-A----LN	--I-E-E--S--------------------
	Prosthecochloris marina	110023770	----AEK-SE---SVTQ-I-ARYKEE	-S-DD---D-A---YLN	-QI-SGH---I------------A------
	Prosthecochloris sp. ZM	114618152	----AEK-AE---NAK-Q--QRYQQE	--P-V---E-----YLN	-QI-GDA--SI--Q---E-----A------
	Prosthecochloris sp. GSB1	094083107	--K-AEK-SE-TSSAFD----RYRNE	--D-ALR-D-SR-IYLN	-QI-S-Q--KI--Q---------E------
Ignavibacteriae	*Melioribacter roseus*	397690716	-----QKN-ELEE-VVEQLA-K-PEQ		-KV-KTIL--M--EL--QR--SEGL-----
	Ignavibacterium album	385809228	-----AKN-ELAEFVKTSLA-KYPEQ		-KV-A-LL--M--EL--QR--TEGI-----
Other Bacteria	*Gordonia polyisoprenivorans*	378717577	-Q--D-K-DELKEQILTQLA-Q-EGR		-KE-GAAFRS-T--LV-QR--T-HF-I---
	Gordonia paraffinivorans	493955506	-Q--D-K-DELKADILEQLADK-EGR		-KE-GGAYRALT--LV-QR--T-HF-I---
	Gordonia bronchialis	262202098	-Q--DEK-DELKDDIL-QLG-Q-EGR		-KE-GAAYRS-T--LV-QR--T-HF-I---
	Gordonia araii	494531914	-Q--DEK-DELKA-LLEQLGGQ-EGR		-KE-GAAYRSLT--LV-QR--T-HF-I---
	Mycobacterium gilvum	145224603	--A-D---NE-KA-VVERLA-QYAGR		-KE-GAAYRSLT--LV-QR--T-HF-I---
	Corynebacterium bovis	497951292	-Q--DEALA-NLD-VVETLAGQ-PDR		-RE-RAAHN--T-TIV-DR--TEGF-I---
	Corynebacterium resistens	336325779	-Q--D-ALAENMQA-VDEL--E-PER		-GE-RAAHN--T-QLV-QR--EEGF-I---
	Nocardia farcinica	54025857	-Q--EEKIDE-KLAVLDRLA-Q-AGR		-KELGAAFRS-T--LV-QR--T-GF-I---
	Mycobacterium liflandii	443490346	-A--NQ--EE-KA-VLERLADTYEGR		-KE-GAAFRSLT--LV-QR-VT-HF-I---
	Rhodococcus coprophilus	555592905	-Q--D---DE-KV-VLEQIAPK-EGR		-KE-GAAFRSLT--LV-QR--R-QF-I---
	Gordonia terrae	490121385	-Q--D-K-DELKADIL-QLG-Q-EGR		-KE-GGAYRSLT--LV-QR--T-HF-I---
	Gordonia soli	494899217	-Q--DSK-DELKADVLEQLAGQ-EGR		-KE-GAAYRSLT--LV-KR--T-HF-I---
	Cellulomonas fimi	332670053	-QT-EN-LDE-KA-VVGQLA-Q-EGR		-KELSAAYRS-Q--LI-QR--T-GF-I---
	Rhodococcus ruber	490033249	-Q--DE--DE-KV-VLEQVAPN-EGR		-KE-GAAFRSLT--LV-QR--R-QF-I---
	Carboxydothermus hydrogenoformans	78044607	-Q--DEYLENLK--LTT-F--TYPEE		-LT-KGILDSIQ-EIV-QK--KEGI-I---
	Clostridium papyrosolvens	490755727	-QD-DAKVSELTE--Q-ALA-QYPEM		-NK-KDA-YKL----V-EY--KEG--V---
	Candidatus Latescibacter	551163936	-T--NATLAT-FEKA-E-FE-KYPES		-TLLKNAFD-IVREI--S---QE-V-I---
	Alicyclobacillus acidoterrestris	544884231	-LA-EAAISEVNE-VQEHFA-Q-PEM		-ND-A-VL--IL-EEV-SA--NEGI-P---
	Alicyclobacillus hesperidum	493492321	-LA-EEALSNLNE-VQ-HFA-A-PER		-AE-A-IM--IL-EEV-RA--QEGI-P---
	Kyrpidia tusciae	295696164	-LA-E-AIR-VEE--QEHFA-Q-PEG		AAD-A-VL--IL-EEV-SA--NEGM-P---

Figure S17

Partial sequence alignment of the protein polynucleotide phosphorylase/polyadenylase, showing a 17 aa insertion (boxed) that is found in all members belonging to the order *Chlorobiales*

Group	Species	Accession	119		164
Order *Chlorobiales*	*Chlorobium phaeobacteroides*	500067752	DLKPPSSGVCERNCPENITLA	LHAGKKL	SQTFEFKIVVASREDYDW
	Chlorobium limicola	189347255	---A------NK-N-Q--E--	MRS--AE	ISS--------D----L-
	Chlorobium chlorochromatii	78188483	---A-----A-K-N-A--R--	-E-APEE	QRR----M-I-N----E-
	Chlorobium phaeoclathratiforme	194337298	----------DQ-N----E--	-A-VLPH	R-N------------E-
	Chlorobium ferrooxidans	493409287	------T--SDK-N----A--	-KQKDHA	NRLI-L-----D----L-
	Chlorobium phaeovibrioides	145219296	--------ESGK--M------	FK---E-	QKS----M---D-N--L-
	Chlorobium phaeobacteroides	189500807	---A----ESG-I-TS--E--	VS-AEP-	KKS----LLL--------
	Chlorobium luteolum	78186318	--------RDKE--M---DI-	-N--EGE	RKRY-----A-D----L-
	Chloroherpeton thalassium	193215257	-I-T---EMS-W--YR-LE-S	IAEA-SG	VLKT-----LS-EG--R-
	Prosthecochloris aestuarii	194334507	--------SSSN-HET--RI-	-K-PPAE	QAL------I-GK---E-
	Prosthecochloris ethylica	175187643	-I-------SQN--TQ--L--	-Q-PPEQ	RH-W-----I-D-N--N-
	Prosthecochloris marina	110023272	---A----ES-K--LK--EYV	IASSVLQ	KK-----LII---Q--E-
	Chlorobaculum thiosulfatiphilum	139457424	-I-A-----AAE--AT-FA--	-DT	PER------------E-
	Chlorobaculum parvum	193213357	-I-T-----A-Q--MD--P--	-AS	PKR---------E---L-
	Chlorobaculum tepidum	21673157	-I-A-----M-H--AA-F---	-NE	PER---------EA--L-
	Candidatus Chlorobium masyuteum	166807843	------T--SDK-N----A--	-KQKDHA	NRSI-L---I-D----L-
	Chlorobium sp. N1	TCD47685	--------CA-E--M--VE--	-A-VTAE	RKR------A-D-R--L-
	Chlorobium sp. KB01	076792264	------T--SDK-N----A--	-KQKDHA	NRLI-L-----D----L-
Ignavibacteriae	*Ignavibacterium album*	385808941	---C----MMKK-LYD--YFL		KP-D-V-F-IGT------
	Melioribacter roseus	397689554	---C---KMEKK-RY---EFL		KPGD-V-F-IGD-N--E-
Other Bacteria	*Uliginosibacterium gangwonense*	517434067	---A-G--EVSK-RL---P-L		RASD-L--IISDEQ--L-
	Methylotenera mobilis	253997441	-V-T-D--ELKN-LWG-LAHL		KKAD-V-F-LC--A--E-
	Sulfuricella denitrificans	543962518	-I-T-G--EA-K-RWK-LE-L	T	P-D -V-F-LCGE---Q-
	Nitrosospira multiformis	82703824	---T-G--EV-K-LWS-VQYL		TSHD-V-F-LCDED--R-
	Methylovorus glucosetrophus	254000097	-I-T-G--EL-K-LWG-LAHL		KAQD-V-F-LCDHA--L-
	Thauera linaloolentis	490464234	---A-G--EV---LYA--A-L		TPHD-I---L-GEA--E-
	Thauera terpenica	544831712	---A-G--EL---L-A--P-L		RAQD-V-M-L-DEA----
	Azoarcus toluclasticus	517818178	---A-G--EVA--RW--LAHL		AAHD-I---L-DEA--A-
	Methylotenera versatilis	297539673	-V-T-D--ELKN-VWS-LDHL		KSSD-V-F-LC-----Q-
	Methylobacillus flagellatus	91776688	-I-T-D--EVAK-HWD-LSRL		KPND-V---LC--A--E-
	Sideroxydans lithotrophicus	291612993	-I-T-G--EVQK-LWG-LQHL		GRHD-I-F-LCGEA--Q-
	Thiobacillus thioparus	517331384	-I---E--EEA--RW--VAHL		TSRD-I-F-L-N-A--E-
	Dechlorosoma suillum	372487166	---A-G--EV---LWS-LEYL		TPHD-L-L-L-D-A----
	Aromatoleum aromaticum	56476931	---A-G--EVA--RWD-LARL		NERD-V---L-D-L--E-
	Dechloromonas aromatica	71909659	---A-D-AESA--LW--L-VL		TPRD-I---I---G--E-
	Thauera phenylacetica	490513686	---A-G--ELA--RL--LA-L		NARD-L-F-L-DAA--A-
	Xanthomonas campestris	498060970	-I-T-A--EEA--RW--LP-L		TARDQI-F-IC--A--E-
	Stenotrophomonas maltophilia	344208553	-I-T-G-AEAA--RL--LP-L		TARDQI-F-ICN------
	Acidithiobacillus ferrooxidans	MBU2824517	---S-D--E----LW--LP-L		NPQDQI-F-LC--A----
	Fluviibacter phosphoraccumulans	WP_162050708	---A-G--ETDK-LLS--PDL		R--D-V---I---A----
	Sulfuritalea hydrogenivorans	WP_041100787	---A-G--EEAK--W---G-L		TAHD-L-F-L--------
	Azonexus hydrophilus	WP_205203486	---A-A--E----LWS-LRHL		KAGD-I--II-D-A----
	Acidithiobacillus ferriphilus	MBW9249360	---S-D--E----LW--LP-L		NPQDQI-F-LC--A----
	Sulfuritalea hydrogenivorans	BA031070	---A-G--EEAK--W---G-L		TAHD-L-F-L--------
	Corallococcus coralloides	AFE10952	-M-T----E-D--DYR-L-SM		NAND-L---IG--V--E-
	Xanthomonas gardneri	493497825	-I-T-A--EEQ--RW--LP-L		TARDQI-F-IC--A--E-
	Xanthomonas vasicola	515684535	-I-T-A--EEA--RW--LP-L		TARDQI-F-IC--A--E-
	Dyella ginsengisoli	516030927	---A-D--ESA--LWA-LAHL		LPHDQV---I---A--E-
	Xanthomonas axonopodis	346725939	-I-T-A--EEA--RW--LP-L		TARDQI-F-IC--A--E-
	Rhodanobacter spathiphylli	495083596	---A-G--ESA--LWS-LDHL		LPHDQI---I------E-
	Stigmatella aurantiaca	488692977	-M-T----ESD--DVR-FSSM		NARD-M-F-IG-----E-
	Myxococcus stipitatus	442317511	-M-T----ESS--DLR-FASM		NARD-L-F-IG-----E-
	Corallococcus coralloides	383452467	-M-T----E-D--DYR-L-SM		NAND-L---IG--V--E-
	Desulfobacca acetoxidans	328953829	---C-G--MAQH-YLK-LDYL		TEKD-L-F--SN-R-F--

Figure S18

Partial sequence alignment of the protein 7-carboxy-7-deazaguanine synthase QueE, showing a 3-7 aa insertion (boxed) that is found in all members belonging to the order *Chlorobiales.* While other *Chlorobiales* species contain a 7 aa insertion, *Chlorobaculum* species have a 3 aa insert in this position

Group	Species	ID	47	(boxed)		93
Order *Chlorobiales*	*Chloroherpeton thalassium*	193213861	RGVGKTTAARILAKGLNCEKILKD	EA	YRETVSE	PCGECESCRDFDAG
	Pelodictyon phaeoclathratiforme	194336682	----------VF--A---Q-MME-	AE	-LQQ-T-	--------------
	Chlorobium ferrooxidans	493410721	----------VF--AV--QRMME-	PV	-LQE-T-	------------S-
	Chlorobium chlorochromatii	78188902	-----------F-RA---Q-LID-	AD	-LQQ-T-	--------------
	Prosthecochloris aestuarii	194334277	----------VF--AV--Q-MID-	PV	--QE-T-	--------L-----
	Chlorobaculum parvum	193212992	----------VF--AV--QRMIE-	PK	-LKE-T-	--------------
	Chlorobium limicola	059138195	----------VF--AV--QR-ID-	PE	-LRK-T-	--------------
	Chlorobium phaeobacteroides	119357453	----------VF--AV--I-MTE-	KD	-LQH-T-	-----------E--
	Chlorobium luteolum	78187181	----------VF--A---QRMID-	AD	WLRD-T-	-----------ET-
	Chlorobium phaeovibrioides	145219538	----------VF--A---QRMIE-	AD	WLRD-T-	--------------
	Chlorobium tepidum	21674147	----------VF--AV--QRMID-	PQ	-LKE-T-	---V----------
	Prosthecochloris marina	110023130	----------VF--AV--SRMIE-	PV	-LKE-T-	-----------EQ-
	Chlorobaculum thiosulfatiphilum	139457273	----------VF--AV---RMID-	PK	-LKE-T-	--------------
	Prosthecochloris ethylica	175186743	-----------F--AV--HRMIE-	-T	--QE-T-	-----------E--
	Chlorobaculum limnaeum	069809131	----------VF--AV---RMID-	PK	-LKE-T-	--------------
	Candidatus Thermochlorobacteri GBChlB	KER09939	-------S------A-----V-T-	D-	--RD-A-	---V-D------
	Chlorobaculum sp. 24CR	129051538	----------VF--AV---RMIE-	PK	-LKE-T-	--------------
	Chlorobium sp. 445	PI047289	-------T------A----R--T-	--	--HE-A-	---M----------
	Chlorobium sp. N1	131353650	----------VF--A---QRMIE-	PD	WLQN-T-	------------S-
	Prosthecochloris sp. HL-130-GSB	085659383	-----------F--AV---RMIA-	-T	--HD-T-	------------S-
	Prosthecochloris sp. ZM_2	114608329	-----------F--AV--HRMIE-	-T	--QE-T-	-----------E--
	Prosthecochloris sp. GSB1	094082258	----------VF--AV--SRMIE-	PV	-LKD-T-	------------S-
Ignavibacteriae	*Ignavibacterium album*	385809646	-------T------A---LQPVD		F-	--NK-DM--AINEM
	Melioribacter roseus	397690783	-------T------V---LNP-D		G-	--N---M-KS-HNS
Other Bacteria	*Cesiribacter andamanensis*	496488478	-------C------TI--QNL-S-		T-	--N-----KS-N-S
	Odoribacter splanchnicus	325279833	-------C---F--TI--LNLQPN		A-	--N-----KA-N--
	Odoribacter laneus	496427542	-------C---F--TI--QNLQPN		A-	--N-----KA-NT-
	Sphingobacterium paucimobilis	544633475	-------C------TI---N-SSS		T-	A-------KS-AG-
	Flexibacter litoralis	392399356	-------CS-----TI--LN-TPE		T-	--D--S--KS-N--
	Rhodothermus marinus	268318150	----------L---AI--QTP-EE		--DHA-	--RR--A-VA-EE-
	Nafulsella turpanensis	516343857	-------C------TI--QNLQAE		GGP-DI-	--N-----KS-NTS
	Butyricimonas synergistica	517147223	-------C---F--TI--MNLQPN		A-	--N-----KA-ND-
	Salinibacter ruber	294508590	--------------AI---TPREE		--DGA-	--C--D--ES-E--
	Leadbetterella byssophila	312129467	-------C------TI--QNVSA-		T-	--N--T--KN-AQN
	Pontibacter roseus	517290706	-------C------TI--QN-TPE		V-	A-N------S-NSN
	Algoriphagus machipongonensis	495478098	-------C------TI---NRQD-		Q-	A-------VS-KNN
	Segetibacter koreensis	517442100	-------C------TI---N-SP-		G-	A-NH-H--VS-NE-
	Chitinophaga pinensis	256426033	-------C------TI---NLQP-		G-	A-NQ-H--TS-NE-
	Runella slithyformis	338214761	-------N------TI--QSLTDE		V-	--------VA-QNN
	Emticicia oligotrophica	408674356	-------C------TI--QNLTE-		IS	--N-----KS-NNN
	Cytophaga aurantiaca	517153747	-------N------TI--TN-TA-		T-	A-DQ----KS-NNN
	Cytophaga hutchinsonii	110637639	-------N------TI--TN-TA-		I-	A-D-----KS-NNN
	Niabella aurantiaca	517460269	-------C------TI---HSTA-		G-	A-N--P--IS-NQ-
	Dyadobacter fermentans	255038674	-------C------TI--QNLGD-		V-	A-------VS-QNN
	Arcticibacter svalbardensis	510820038	-------C------TI---NLSAE		T-	A--V-P---A-LN-
	Sphingobacterium spiritivorum	489092257	-------C------TI---H-TSE		V-	A--V-D--KS-QN-
	Cecembia lonarensis	496476319	-------C------TI--QNLTA-		F-	A-N--D--KA-NNN
	Pedobacter heparinus	255530648	-------C------TI--TSLTAE		Q-	A--T----VS-QT-
	Solitalea canadensis	387789585	-------C--V---TI--QTPTA-		F-	A-------LS-KH-
	Nitritalea halalkaliphila	496346710	-------C------TI---HLTE-		F-	A-NQ-D--KA-NQN
	Dyadobacter beijingensis	518785034	-------C------TI--QNLGD-		V-	A-------VS-QNN
	Niabella soli	495860953	-------C------TI---HATPE		G-	A-N--N--IS-NL-
	Alistipes senegalensis	517981349	-------C---F--AI--LAPNGA		-	A-N------S-NE-
	Indibacter alkaliphilus	496326470	-------C------TI---HLTA-		H-	A-NK-D--LA-NNN
	Saprospira grandis	488734264	-------C------L----NPTS-		T-	--D--P--TA-REN
	Porphyromonas gulae	517795486	------SC---F-RAI--LER-P-		G-	A--R----KA--EQ
	Prevotella multisaccharivorax	494849999	-------C---F--AI---NPTQ-		G-	A-N------A-NE-
	Bacteriovorax marinus	374286880	--I---SV---F--AIR--SR-E-		-N	A--T----Q---SA
	Alistipes finegoldii	547311906	-------C---F--AI--LSPNGA		-	A-N------S-NE-
	Rudaea cellulosilytica	517804372	-------I---F--S-----GQSS		D	-----ST---I---
	Xanthomonas sacchari	498026990	-------I---F--S----QGTSA		D	---Q-AA-L-I---
	Arenimonas oryziterrae	551348563	-------I---F--S----RGTGA		-	-----NA-V-I---
	Stenotrophomonas maltophilia	544778658	-------I---F--S----QGTSA		D	---Q-AA-L-I---
	Xanthomonas translucens	489564082	-------I---F--S----QGTSA		D	---Q-PA-L-I---

Figure S19

Partial sequence alignment of the protein DNA polymerase III subunits gamma and tau, showing a 2 aa insertion (boxed) that is found in all members belonging to the order *Chlorobiales*

Group	Organism	Accession	220		268
Order ***Chlorobiales***	*Chlorobaculum parvum*	193212992	GSMRDAQSILDQVIAFA	IDSE	GERAIRYDKVSDLLSYIDDEHFFMVTDA
	Chlorobaculum tepidum	21674147	-----------------	----	-----------E----------------
	Chlorobium luteolum	78187181	----------------S	AQ-G	-DG--S-QN-AE--N-----QL-A----
	Chlorobium phaeobacteroides	189499967	-----------------	VEQD	D--S---E--AE--N----D---A----
	Chlorobium ferrooxidans	493410721	----------------T	AEND	H-GS-S-QS-AE--N------M-G----
	Chlorobium phaeoclathratiforme	194336682	----------------S	AEND	HTGS-T-SG-AE--N------L-E----
	Chlorobium chlorochromatii	78188902	----------------S	SENA	L-GS-T-RG-A---N----DTM-A----
	Chlorobaculum limnaeum	069809131	-----------------	L-N-	-------E-----------DH-----
	Chlorobium limicola	059138195	----------------S	F-R-	SDG--S-A--AE--N----D---A----
	Prosthecochloris aestuarii	194334277	----------------S	VGND	A-KV-A-QG-AE--N----DQ--D-S--
	Chlorobaculum thiosulfatiphilum	139457273	-----------------	L---	---V---E------------------E
	Prosthecochloris marina	110023130	-----------------	-EQD	E--S-H----AE--N----DQ--E--A-
	Chlorobium phaeovibrioides	145219538	----------------S	--	SGEG-S-QG-RE--N-----QL-A----
	Chloroherpeton thalassium	193213861	-A------L-----S-F	L-T	ENEK-E-TN-AA--GD-------A---S
	Prosthecochloris ethylica	175186743	-----------------	-GEN	H-EG-S-QR-A---N-V------A----
	Cand. Thermochlorobacter aerophilum	RFM23307	-A------L-------C	TE-	-KGK-E-AA-A---NI----R--D-S--
	Chlorobaculum sp. 24CR	129051538	-----------------	L---	---V---E--A---------------E
	Prosthecochloris sp. GSB1	094082258	-----------------	-ER-	-D-T---ET-AE--N----DH--A----
	Prosthecochloris sp. HL-130-GSB	085659383	----------------S	----	-RT-VS-EN--G--N--------A-S--
	Prosthecochloris sp. ZM_2	114608329	----------------S	-GEN	H-EG-S-QR-A---N-V------A----
	Chlorobium sp. N1	131353650	----------------T	AEGD	--A--S-RG-A---N-----QL-Q----
	Candidatus Thermochlorobacteri GBChlB	KER09939	-A------L-----S-C	TQD	-V-D-N-AA--E--NV----K--D----
	Chlorobium sp. 445	PI047289	-A------L-------C	TEN	-KGK-E-VA--E--NV--E-RL-D-S--
Larger ***Ignavibacteriae*** Clade	*Chlorobi bacterium OLB7*	KXK57074	---------F---T--C		GTT-T-HQAN-A-NL--QD-L-R---
	Ignavibacterium album	385809646	-G------YF------C		GENVDSET--KI-NL--E-TY-NIS--
	Melioribacter roseus	397690783	-AL---E-LF--I-SYS		GKKVNSEEI-QM-NL--QDIY-RIS--
Other Bacteria	*Rhodothermus marinus*	345304445	-AL---L-AF--AVSLC		GTTL--AELAQA-GVV-LDLY-A---H
	Parabacteroides distasonis	150008698	-G----L--F---VS-T		NGN-T-QA-I-N-NVL-Y-YY-RL---
	Parabacteroides johnsonii	495429919	-G----L--F---VS-T		NGN-T-QA-I-N-NVL-Y-YY-RL---
	Fibrisoma limi	496578583	-GL---L-MF-LNVT-S		AD-T---KE-L-N-HIL-YDYY-KL--Q
	Parabacteroides merdae	547920597	-G----L--F---VS-T		NGN-T-QA-I-N-NVL-Y-YY-RL---
	Capnocytophaga gingivalis	488745336	-A----L--F-R-VS-C		GKT-TRQA--EI-NVL-YDTY-K---L
	Spirosoma luteum	517446762	-GL---L-MF-LNVT--		AD-V---KE-L-N-HIL-YDYY-KL--L
	Thermophagus xiamenensis	498213992	-A----L--F--IV--S		GKR-S-QQ-IEN-NVL-YDYY-KLV--
	Spirosoma linguale	284034966	-GL---L-MF-LNVT--		AD-I---KE-L-N-HIL-YDYY-KL--L
	Clostridium botulinum	153941061	-A----L-------STG		DGKVE--Q-L-M-GLVTN-NLLRI--S
	Clostridium sporogenes	489578394	-A----L-------STG		DGKVE--Q-L-M-GLVTN-NLLII-NS
	Candidatus Symbiobacter	550990475	--L---L-LT--A---G		GG-VQTPS-REM-GCV-RS-VYALL--
	Verminephrobacter eiseniae	121607506	------L-LT--A---G		SGQLQEAA-RQM-GAV-RSYV-RLI--
	Aromatoleum aromaticum	56476074	------L-L---A--HG		SGRVEEEQ--HM-GTVG-D-LYA-L--
	Nitrosospira multiformis	82702979	------L-----A---G		-GK-EEAG-R-M-GAV-QGYL-DLLE-
	Cupriavidus taiwanensis	516633018	------L-LT--A--YS		AGQVSEEA-RGM-GA--QGYLVQLL--
	Methylibium petroleiphilum	124267720	------L-LT--A---G		AGTLQEAG-RQMIGAVGRS-VYALI--
	Cupriavidus necator	339326421	------L-LT--A--YS		AGQVSE-A-RGM-GA--QGYLVQLL--
	Ralstonia pickettii	549447903	------L-LT--A--YS		AGQVSEEA-RGM-GA--QGYLVQLL--
	Caldithrix abyssi	493984161	-----S------L-S-S		GNE-KFED-AKA-GV-HQDE--HL--Y
	Geobacter metallireducens	404498270	-----SL-V----L--C		GDQV-DEE-VS--GVV-RRLLLDA-A-
	Desulfococcus multivorans	527022898	------L-L----MTCT		-GE-THEG-LN--GAA-R-VL-RI-A-
	Geobacter sulfurreducens	39995205	-----SL-V----L--C		GETVND-D-VS--GVV-RRLLLEASA-
	Bdellovibrio bacteriovorus	426405587	-----S--L-----T--		NGPLTRAS-VEI-GLT-RALL-ETLN-
	Mobiluncus curtisii	490107874	--V--SL-----L--GE		A-PKLH--RAVS--G-T-SGLLGGMV--
	Hyphomicrobium denitrificans	488578002	--V--GL-----A--MG		-GHVTAMS-R-M-GLA-RARI-DLAE-

Figure S20

Partial sequence alignment of the protein DNA polymerase III subunits gamma and tau, showing a 4 aa insertion (boxed) that is found in all members belonging to the order *Chlorobiales*

Group	Organism	Accession	146	Insert	199
Order *Chlorobiales*	*Chlorobaculum tepidum*	21673125	LKIFLIADPAERAKRRHAEL	LLKA	G GAAVPSVEALEEEIKQRDRDDEQRTHAPL
	Prosthecochloris vibrioformis	139626144	-----V-------R--Y---	ES-H	— AEGL--PDE--Q--I------RG------
	Chlorobaculum parvum	193213387	--------------------	----	- -------DE-------------------
	Chlorobium ferrooxidans	493410280	-----V--AR------Y---	---S	PE--EL-DLDL--D------EE-TL-E----
	Chlorobium chlorochromatii	78188549	-------NAR------Y---	-A-L	PA-STM-TL-E--A--R---L---T-Q----
	Chlorobium phaeoclathratiforme	194335600	---Y-V--AR----------	TA-S	AE-VEL-DLDS--Q--TK-----AE-E----
	Chlorobium limicola	189345891	--------AR------Y---	AA-G	AS-SEL-D-AI--Q--VE---A-AR--L---
	Chlorobium phaeovibrioides	145220319	-----V--AR-------L--	QA-S	PE-FG--DL-T--A--VE---A-AL-E----
	Chlorobium phaeobacteroides	119356255	--V--T--VRQ--M--Y---	AV-S	-DPSEL-CL-D--R--LD---A-AE-E----
	Chlorobium luteolum	78187587	-----V--ARQ--M--LL--	QA-S	PD-TGL-DL-T--R--ME---A-AE-EL---
	Chloroherpeton thalassium	193213832	--V-M---AK------Y---	KA-S	PS-DPGVTL-T--Q--L---E--RT-AI---
	Chlorobaculum limnaeum	2501895123	-----V--------------	S---	- ------L-E--A--VK--K---E------
	Chlorobaculum thiosulfatiphilum	139456778	-----V-----------V--	S---	- ------L-E--A----------E------
	Prosthecochloris marina	110022325	-----V--AS------YT--	VR-S	-GSNN---IKE--Q--L----S-AE------
	Prosthecochloris ethylica	175187789	-----V--A----R--Y---	VQ-N	-NP--L--LDE--R--V------AE-A----
	Prosthecochloris sp. CIB 2401	068867779	-----V-------R--Y---	ES-H	- AEGL--LDE--Q--I------RE------
	Prosthecochloris sp. HL-130-GSB	085660087	-----V---S------Y---	RQ--	-SND-L-TLAE-----V------AE-R----
	Chlorobaculum sp. 24CR	129050436	-----V---R---R------	S---	S ------I-E--A--VK--------E----
	Prosthecochloris sp. GSB1	094081959	-----V--V----R--YE--	I--K	-TKEGL-PLGE-TL--EK-----AT------
	Chlorobium sp. 445	PIO46941	----MT-SVK------YE--	-E-S	KT-TVDITIAE-----A---KA-TE-SI--M
	Candidatus Chlorobium masyuteum	NHQ59323	-----V--AR------Y---	---S	PE--EL-DLDL--D------EE-TL-E----
	Prosthecochloris sp. ZM_2	114608949	-----V--A----R--Y---	VQ-N	-NP-SL--LDE--R--V------AE-A----
Larger *Ignavibacteriae* Clade	*Chlorobi bacterium OLB6*	KXK35372	V-V--V--IE--TR--F---	K	S SGQEV-MDVVRKQLEE--AK-S--ENS--
	Chlorobi bacterium OLB7	KXK53034	----MV-NLDA--A---V--	Q	H QGTAIGLDN-AN-LAE---Q-ST-H-S--
	Ignavibacterium album	385809645	V----T-SID---R--AL-F		AQKGQQVDI-KVKQN-LE---I-SS-DVS--
	Melioribacter roseus	397690792	V--Y-T-SLD---R--FK-Y		QE-SVNI-FDDVKANLAK--EI-SG-DV---
Other Bacteria	*Alistipes senegalensis*	517981532	--L-MT----V--D--YK--		TGKGMTVTM-EV-RN-RE--KA-MS-AVS--
	Anaerophaga thermohalophila	498105936	----MT-S-EV--R--YD-M		VAKGES--FDEILKNV-E--EI-ST-SES--
	Alistipes shahii	479186333	--L-MT--T-V--C--YK--		TGKGMSVTM-EV-RN-RE--KA-MS-AVS--
	Bacteroides gallinarum	517495756	----VT-S-EI--Q--YE--		KAKGEEAGFDEIL-NV----YI-QN--VS--
	Anoxybacillus ayderensis	085787979	V----L-SVE-------E-N		VARGIPSNL-Q-K---A----I-SE-SV---
	Peribacillus faecalis	190999908	--V----SVD-------T-N		LAKGFPSDL-T-KQ--AD---Y-SE-EV---
	Pseudomonas aeruginosa	190306863	V-V----SVE------YE-N		VKKGIPSDL-T-KK--AE--KR-ME-E----
	Weizmannia coagulans	013859522	-----L-SAD---------N		VAKGIPSDLSR-KQ--EE---L-SE-EVS--
	Lactococcus hircilactis	153497060	-----V-SVD------YK-N		KLKGIPTEL-K-KA--AE---K-ST-ANS--
	Thermolongibacillus altinsuensis	132947059	V----L-SVE---------N		IARGFPSDL-Q-K---AR--KI-SE-AV---
	Peribacillus muralis	057914352	V-V----SVD---Q---Q-N		LLKGFPSDI-K-R---AT--KL-SE-EVS--
	Pseudomonas oryzihabitans	145153512	-----T-GAE---Q--YLQ-		KQKGENV-LP--L---RE--AR-S--SV---
	Mesobacillus selenatarsenatis	041965682	V-V--L-SVE---Q-----N		IQKGFPSDL-K-K---AA--KI-SE-VV---
	Vibrio vulnificus	TDL92960	V-V----SVD---Q---Q-N		ILKGFPSDI-K-R---AT--KL-SE-EVS--
	Gimesia panareensis	145449005	C-F------E---R--YD-M		QAQDKKITL-EILQQ-Y---QR--S--V---
	Anaeromyxobacter dehalogenans	011420571	-F--V-T-E---R------		LAKGDPSTF-EVLADQLK---Q-SE-EI---
	Anaerophaga thermohalophila	515147035	----MT-S-EV--R--YD-M		VAKGER--FDEILKN--E--ER-ST-SES--
	Alistipes finegoldii	390946600	----MT---KV--C--YD--		RAKGDDV-L-EI-RNVRE--KA-MS-AIS--
	Bacteroides fragilis	492365035	----VT-S-EI--Q--YD--		KAKGQEA-F-EIL-NV----HI-QT-EVS--
	Paraprevotella xylaniphila	495905258	----VT-S-EI--R--YD--		QAKGEEG-Y-EIL-NV-E---I-TS--VS--
	Bacillus coagulans	516119894	-----L-SAD---------N		VAKGIPSDLSR-KQ--EE---L-SE-EVS--
	Lactobacillus paracasei	511484647	V--YMV-SV-------YK-N		IQKGIETPLAT--Q--AE---K-SH-AVS--
	Clostridium ultunense	491760968	-----T-SIQ---M--YR--		WEKGHS-NLSQ------K---L-AE-S-S--
	Eubacterium cellulosolvens	490739887	---Y-T-STRT--E--YK--		LEKGQEADLA-I-KD-E---YQ-MH-E-S--
	Clostridium lentocellum	326791349	-----T-SVE---Q--YK-Y		VQKGEK-CL-T-KV---E--YQ-SH-KIS--

Figure S21

Partial sequence alignment of the protein cytidylate kinase, showing a 4 aa insertion (boxed) that is found in all members belonging to the order *Chlorobiales*.

			264		311
Order *Chlorobiales*	*Prosthecochloris aestuarii*	194333263	LFNICKASNPRTYFAEDEKDIQDEWL	LEN R	QPVQSAGVCGATSTPMWL
	Chlorobium phaeobacteroides	189499431	-----RE----S--IA-QTGL----F	YD- G	R--G-V-----------
	Chlorobium limicola	189345888	--G--RSA---S--I--IE-LE-S-F	RD-EG	S--E-I-I----------
	Chlorobium ferrooxidans	493410283	-Y---RDA---S--I--VEE--P---	-SLNG	SG-A---I----------
	Chlorobium luteolum	8187590	-Y---RDA---S--I--TG-LDEG--	K-ANG	S--G-V-I----------
	Chlorobaculum parvum	193213390	-YS---DA--HS--I--VDE--P--F	VGENG	K--E-V-I----------
	Chlorobium chlorochromatii	78188557	-------A----F-I--IEE-NP--F	AAHEG	KA-E-V-I---------H
	Chlorobaculum tepidum	21673122	-YS---DA--HS--I--VDE-RP--F	VGENG	K--E-V-I----------
	Chlorobium phaeovibrioides	145220322	-YA---EA--ESH-I--VEEL-S--F	TTTQG	KT-E-I------------
	Chlorobium phaeoclathratiforme	194335597	-YH---EA---S--I-EIEE-EER--	RNSDG	RA-ATV-----------H
	Chloroherpeton thalassium	193213833	--EV--DA-SN-H-I-NQSELERA-F	-RPDD	SL-E-V-----------V
	Prosthecochloris ethylica	NUK47677	-Y----S----S--V-N-E--DRD--	MRN G	S--DNV------------
	Prosthecochloris marina	110022730	------Q--S-S--I-N-N-L----F	SDN G	ST-ENV------------
	Chlorobaculum thiosulfatiphilum	139456775	-YS--RDA--KS--I--VDE--P--F	VGENG	K--E-V-I----------
	Chlorobaculum limnaeum	2501895126	-YS--RDA--KS--I--VE--LP--F	VGDDG	K----V-I----------
	Prosthecochloris aestuarii	012505013	--------------------------	--- -	------------------
	Cand. Thermochlorobacter aerophilum	RFM22955	—-GV---A--------H-DEL-AD-F	MREDG	TL-E-V-----------
	Chlorobium sp. 445	PI048543	—-GV---A--------H-GEL-A--F	QRSDG	L--E-V-----------
	Chlorobium sp. KB01	076792162	-Y---RDA---S--I--VEE--P--F	-SLND	SS-A---I----------
	Prosthecochloris sp. ZM	114617735	--------------------------	--- -	------------------
	Prosthecochloris sp. HL-130-GSB	085660515	-Y----E--S-S--V---H---R---	I-K G	---DNT------------
	Prosthecochloris sp. ZM_2	114607604	-Y----S----S--V-N-E--DRD--	MR- G	S--DNV------------
	Chlorobaculum sp. 24CR	129050439	-YS--RDA--HS--I--VEE-LP--F	VGDDG	K----V-I----------
	Chlorobium sp. N1	131354410	-Y---REA--ESH-I--VEE-DP--F	RLKDG	GTAE-V------------
Larger *Ignavibacteriae* Clade	*Chlorobi bacterium NICIL-2*	KXB97964	--DVARSE------I--VSEL-S--F		EGAE-V-IT------Q-
	Chlorobi bacterium OLB6	KXK35371	--EVSRSA--A-H-I-EIDE--P--F		TN-NTV-IS------Q-
Other Bacteria	*Pontibacter roseus*	517290586	-YSV--QH--NS--V-N-EEL-K--F		ENA--V-I----------
	Nitritalea halalkaliphila	496346574	-Y-V--ER--N-H-VSNSAQV--S-F		G-G--V-I----------
	Cyclobacterium marinum	343084681	-YQV--HI---S--V-N-TE-NSD--		N-ADKI-I----------
	Cyclobacteriaceae bacterium	499125948	-Y-V--ER--N---VSNPEEVSAD-F		S-N-TV------------
	Rhodonellum psychrophilum	518425835	---V--ER-QN---VSNSEQVDK--F		LEG-TI-I----------
	Cand. Azobacteroides pseudotrichonymphae	212550484	---E-RRA-LN--LIS------S--F		NGIK-V-I-------K--
	Echinicola vietnamensis	431797370	-YQV---Q---S--V-N-GE-DPD-F		ANGDKV-I----------
	Echinicola pacifica	517284044	-YQV---E---S--V-N-NELDPK-F		D-QDKV-I----------
	Cesiribacter andamanensis	496487984	-YAV--HH--QS--I-N-SE-DPA--		EGK--V-I----------
	Hymenobacter norwichensis	551196148	--SVVNKT---S--V-N-QE-DE--F		HGAE-V-I----------
	Mariniradius saccharolyticus	495907062	--QV-LSE---S--V-N-TE-DPS-F		T-QDKV-I----------
	Cecembia lonarensis	496476169	-Y-V--EK--N---VSNPDQVLSD-F		VSNETV-I----------
	Marivirga tractuosa	313675572	-Y-V--DK--N---ISNTTELDKS-F		-END-V-I----------
	Flexithrix dorotheae	522016070	-Y-V--KF---S--I-N-SE-AS--F		S-NDKV-IS---------
	Cyclobacterium qasimii	527058107	---V--EK--Q---VSCPEEVDAK-F		KAGE-V-I----------
	Thermonema lapsum	166917974	--EV-RSE--HS--I---AE--E---		EGAR-V-I----------
	Chitinophagales bacterium	MBX7109056	---V---A--NS--VSE-A-L-Q--F		RSA--V-I-------L-
	Hymenobacter wooponensis	135529319	--SVVNKT---S--V-N-QEL----F		-GME-V-I----------
	Candidatus Kapabacteria bacterium	TAE32473	-Y-VA---------I--VQEV-----		EG-KTV-IS------Q--
	Cyclobacterium plantarum	166150147	--QV--GV---S--V-N-Q--NPD-V		R-ED-V-I----------
	Raineya orbicola	101358740	--AV-QKA---S--V----EL-P--F		ENCE-I-I----------
	Algoriphagus aquimarinus	146915010	---V--DT--N-F-VSNSEE-ESD-F		IENDRV-I----------
	Cyclobacterium lianum	073090759	---V-REK------VSSPEEVDA--F		AEGE-V-I----------
	Pontibacter korlensis	046308989	-YSV--QH--NS--V-N-EEL-R--F		AE-G-V-I----------
	Nitritalea halalkaliphila	009055752	-Y-V--ER--N-H-VSNSAQV--S-F		G-G--V-I----------
	Catalinimonas alkaloidigena	089684683	-Y-V--ET--H---VSTRSEV-SA-F		--HDTV-I----------
	Cyclobacterium plantarum	166146163	---V--EK--Q---VSSPEEVNPD-F		SEG--V-I----------
	Solirubrum puertoriconensis	059067372	--GVVNHE---S--V-N-QEL----F		DDAA-V-I----------
	Marivirga sericea	085516784	-YQV---Q--NS--I-N--EVDPL-F		EGQKTV-I----------
	Penaeicola halotolerans	226390616	-YQV-LQE---S--V-N-TE-SN--F		EKSDKV-I----------
	Siccationidurans arizonensis	SFP99765	-YQV-LET--Q-H-ISNVAE--P--F		G-G--V-I----------
	Cecembia rubra	211299928	-Y-V--DK--N---VSNPEQVNLD-F		SANE-V-I----------
	Sporocytophaga myxococcoides	028981416	-YSV---E-EKS--V-N-LELDPN-F		K-SY-V-I----------
	Siccationidurans arizonensis	092675538	--SVVN-V---S--I-N-AEVDE---		AGAEKV-I-------L--
	Aquiflexum balticum	084119878	-YQV--NI--KS--V-N-TE-DLS-I		N-QDKV-I----------
	Mucilaginibacter pankratovii	191189761	-YQV-REA--HC--VSGTN---AG-F		S-G-KV-I----------
	Algoriphagus machipongonensis	495474052	-YQV-LGQ---S--I-N-TELDP--F		KSNDKV------------
	Cytophaga hutchinsonii	110636514	-YGV--QE-E-S--V-N-TE-EPS-I		RATDNV-I----------
	Indibacter alkaliphilus	496324935	-HQV--GV-E-S--V-N-TE-DPR--		MHAD-V-I----------
	Alistipes indistinctus	496424989	-YQT-L-H---S-TV---TEL-PA-F		DNCR-V-I-------K--
	Cytophaga aurantiaca	517152557	-YGV--QE-E-S--V-N-NE-EES-I		RSTDKI-I----------
	Hymenobacter aerophilus	518788809	--SVVNQT---S--V-N-QELDEA-F		HGAD-V-I----------
	Alistipes putredinis	548242045	--EV-RRA---S-NV-EASELRP--F		EGAE-V-I-------K--
	Hymenobacter aerophilus	518790560	-YQV--DT--A-H-ISKID-LDPA-F		--G--V-I---------Q
	Pedobacter heparinus	255533499	-YDV--KY-DNS--ISNTEE-DK--F		S-GDKV-I----------
	Nafulsella turpanensis	516343586	-YSV-RQQ-EKS--I-N-SELEPS-F		NGTK-V-I----------

Figure S22

Partial sequence alignment of the protein 4-hydroxy-3-methylbut-2-enyl diphosphate reductase, showing a 2-5 aa insertion (boxed) that is found in all members belonging to the order *Chlorobiales*

Group	Species	Accession	70		119
Order *Chlorobiales*	*Chloroherpeton thalassium*	193214979	KRFMGRTQDEVPEETKMVPYQIVS		EGNQARVKIGDKTHSPQEISAMILQK
	Prosthecochloris vibrioformis	139626732	------RY----N-K-IA--KVIN		ANGE---QV---NY--------V---
	Chlorobium ferrooxidans	493411055	------KY----N-K--A--E-IN		-SGE-----N--IY----V-------
	Chlorobium phaeoclathratiforme	194337182	------KY--IND-K-LA--E-IN		--GE-----N--IY----V-------
	Chlorobaculum tepidum	4928210	------KY----N-K-LAS-DV-N		--GY-K-------Y-----------
	Prosthecochloris aestuarii	194333589	------KF----N-K-IA--KV-N		INGE---E-D--NY-----------
	Chlorobaculum tepidum	4928210	------KY----N-K-LAS-DV-N		--GY-K-------Y-----------
	Chlorobium luteolum	78186504	---I--KF----N-K-IA--EV-N		--GE-----N---Y----V-------
	Chlorobium phaeovibrioides	145219963	---V--KF----N-K-IA--AV-N		--GE-----N---Y-----------
	Chlorobaculum parvum	193212312	------KY----N-K-FAS-DV-N		--GD------E-SY-----------
	Chlorobium phaeobacteroides	119356695	---V--KF----N-K-LA--D--N		--GE-----N--AY-----------
	Chlorobium limicola	189346291	------KY----N-K-LAS-DVIN		--GE-----N--IY----V-------
	Chlorobium chlorochromatii	78189848	------RF---G--K--A--ELIN		DSGE-----N--VY--------V---
	Chlorobaculum thiosulfatiphilum	_139457351	------KY----N-K-FAS-DV-N		--GD-K---A-KSY-----------
	Chlorobaculum limnaeum	2501895415	------KY----N-K-LAS-DV-N		--GD-K------SY-----------
	Prosthecochloris ethylica	175187396	------KF----N-K-IA--KV-N		VNGD---E-----Y--------V---
	Prosthecochloris marina	110023859	------KF----N-K-IA--KV-N		TNGE---E-D---Y-----------
	*Chlorobium sp. N1_*	131357844	---V--KF----N-K-IA--EV-N		--GE-----N---Y-----------
	Chlorobium sp. KB01	076789965	------KY----N-K--A--E-IN		-SGE-----N--IY----V-------
	Chlorobium sp. 445	PI047222	------GYA--T--I-----EVIN		--GQ---R-SG-VY-----------
	Chlorobaculum sp. 24CR	129051597	------KY----N-K-FAS-DV-N		--GD-K-------Y-----------
	*Prosthecochloris sp. HL-130-GSB_*	085659873	------KF----N-K-IA--KV-N		VNGD---E-----Y-----------
	Prosthecochloris sp. ZM_2	114608885	------KF----N-K-IA--KV-N		VNGD---E-----Y--------V---
	Prosthecochloris sp. CIB 2401	_068867518	------RY----N-K-IA--KVIN		ANGE---EV---NY--------V---
	Prosthecochloris sp. GSB1	094082850	------KY----N-K-IA--KV-N		VNGE--ADV-G-VY-----------
	Cand. Thermochlorobacteriaceae GBChlB	KER10339	------SYS--TS-M-L---EV--		GN-SVT-RADG-EFT----------
	Candidatus Chlorobium masyuteum	_166808359	------KY----N-K--A--E-IN		-SGE-----N--IY----V-------
	Cand. Thermochlorobacter aerophilum	RFM23545	------SYA--T--I-----EVIN		-NG----R-A-KVY-----------
Larger Ignavibacteriae Clade	*Chlorobi bacterium OLB6*	KXK32426	------QMN-ISD-S-S---K--A	G	DN-T---D-DGRHY--P---------
	Ignavibacterium album	385810489	------FY---ER-RSE---EV-R	G	-N-TV------RLY--P---------
	Melioribacter roseus	397691407	------RI---QQ-I-E---EV-G	S	DNGS-------RLY--P---------
Other Bacteria	*Rectinema subterraneum*	158906686	------RYA--QQ-I-----KVID		N--DV--EA-G-LY-------F----
	Prevotella histicola	MBF1400696	-----E-Y-QCKK-AER--FKV-N		--GYP--Q-D-RKYT----------
	Butyricimonas synergistica	517148057	-----E-Y-Q-SK-INR---KVIR	G	-N-TP--E---RKY-----------
	Paraprevotella xylaniphila	495904162	-----E-Y-Q-QK-ISR---SV-R	G	DN-TP--D-EGRLYT-------V---
	Parabacteroides merdae	491875350	-----E-Y-Q-QK-ISR---KV-R	G	DN-TP--D-EGRLYT-------V---
	Porphyromonas gulae	517795685	-----E-Y-Q-SR-VDR--FKV-R	G	DN-TP--D-DSRLYT----------
	Salibacter halophilus	151166926	-----A-W---KD-VSR---EV-K	G	D--TP----D--MYT----------
	Candidatus Termititenax aidoneus	GBR74801	------LHS--G--E-L---HV-D	G	NNGA-A---D---YT-P---------
	Candidatus Kryptobacter tengchongensis	072149596	------FY---I--ARR---KV-R	G	PN-T---Q---RVY--P---------
	Candidatus Thermokryptus mobilis	HGD81894	------FY---I--ARR---KV-R	G	PN-T---Q---RVY--P---------
	Gracilimonas mengyeensis	142454917	------MF---KD---Q-S-E--K	G	DD-T-----E-RMYA-------V---
	Butyricimonas virosa	HJF71204	-----E-Y-Q-AK-INR---KV-R	G	-N-TP--E---RKY-----------
	Blattabacterium cuenoti	185855363	------MYS--S--L-NT--K-IK	G	GN-TP--S--NRLY-----------
	Theionarchaea archaeon	MBU7016505	------YY---ES-RNT-Q-EV-R	G	SS-D--------VY----------K-
	Candidatus Sulcia muelleri	157877757	------NFS--T--L-NI--K-IK	G	-NDTP--S--N-LYT----------
	Candidatus Butyricimonas faecavium	HJA14976	-----E-Y-Q-DK-INR---KV-R	G	-N-TP--E---RKY-----------
	Thermovirga lienii	014163731	------NYS--A--M-----KV-E	G	PH-SV--VV---QYA-E-------R-
	Longimonas halophila	098062166	------NY-DLG--LDE---EV-R	G	D-GTP---L--REYT----------
	Butyricimonas synergistica	018336875	-----E-Y-Q-SK-INR---KVIR	G	-N-TP--E---RKY-----------
	Chryseolinea soli	119753531	-----KKF---S--K-I-S---E-	G	NN-TV--R---RLFT----------
	Tannerella forsythia	375254445	-----E-F-Q-QK-INRI--KV-R	G	DN-TP--D-DGRLYT----------
	Niabella soli	570743717	------KY---KD-LSHIS-KV-R	G	DNDTI-ID-DGRLYT----------
	Porphyromonas gingivalis	545441075	-----E-Y-Q-SR-VER--FKV-R	G	DN-TP--D-DGRLYT----------
	Parabacteroides distasonis	150008611	-----E-Y-Q-QK-INR---KV-R	G	DN-TP--D-EGRLYT----------
	Bacteroides salanitronis	325298699	-----E-Y-Q-QK-ISR----VAR	G	-N-TP--D-DGRLYT----------
	Parabacteroides johnsonii	495424325	-----E-Y-Q-QK-INR---KV-R	G	DN-TP--D-EGRLYT-------V---
	Bacteroides barnesiae	517540391	-----E-Y-Q-QK-ISR---SV-R	G	DN-TP--D-DGRLYT----------
	Paraprevotella clara	547244504	-----E-Y-Q-QK-IAR---SV-K	G	DN-TP--D-EGHLYT----------
	Thermophagus xiamenensis	498214569	-----TMY--I-N-V-R---KV-K	G	DN-TP--Q-D-RQYT----------
	Pedobacter agri	498288294	-----NSY--SAK-AGR---KV-K	G	DN-TP--E-D-RKYT----------
	Candidatus Latescibacter	551164463	------KY---QN-ISE---KVMK	G	NNET---S----EY--P----FV---
	Nitrospina gracilis	491151244	------SYA--S--M-----KV-K	G	PK-DV-IEVQG-QY--P---------
	Candidatus Solibacter	116626521	------RY---N--M-----KV-R	G	-NGD---D-MG-KY--P-------T-
	Caldithrix abyssi	493986463	------FYN--EK-I-E---KVER	G	NN-VVV-EVMG-KYT-P---------

Figure S23

Partial sequence alignment of the protein molecular chaperone DnaK, showing a 1 aa deletion (boxed) that is found in all members belonging to the order *Chlorobiales.* Except for an isolated exception shown here, the CSI was not found in any other bacterial species in the top 500-BLASTp hits.

			16		59
**Order *Chlorobiales***	*Chlorobium chlorochromatii*	78188056	LHLGHYTGALENWVAQQNQCSA	DGNRAYDT	YFLIADYHTLTTSL
	Prosthecochloris vibrioformis	139626009	--------------ER--ETL-	--S---E-	---V----S-----
	Chlorobium phaeobacteroides	189501390	-----F---------R--ELD-	--Q--FE-	F-------N-----
	Prosthecochloris aestuarii	194335025	---------------R--ETGP	----V---	F-------N-----
	Chlorobium phaeoclathratiforme	194337843	-----F-----S--E----LDE	T-A-V-E-	C-------N-----
	Chlorobaculum parvum	193213678	------S------I----LLQP	--S---E-	C-------S-----
	Chlorobium luteolum	162139381	-------------IE---LRAP	--S-L-E-	C-------S-----
	Chlorobium limicola	189347951	--------------H---LLAE	--S-V-E-	C-------I-----
	Chlorobium phaeovibrioides	145220533	--------------V---LTNP	--T-GCE-	C-------N-----
	Chlorobium ferrooxidans	493409794	-----------S--E----RNE	A-E-V-E-	C-------N-----
	Chlorobaculum tepidum	21672854	-------------I----LLHP	--S---E-	C-------S-----
	Chlorobaculum limnaeum	2501894004	-------------I----LLHP	--S---E-	C-------S-----
	Pelodictyon phaeoclathratiforme	012509488	-----F-----S--E----LDE	T-A-V-E-	C-------N-----
	Chlorobaculum thiosulfatiphilum	139456449	-------------I----LLHP	--S---E-	C-------S-----
	Prosthecochloris marina	110024017	---------------R-DELDT	G-K-VFE-	--------S-----
	Candidatus Chlorobium masyuteum	166807408	-----------S--E----RNE	A-E-V-E-	-------CN-----
	Cand. Thermochlorobacteria aerophilum	KER10736	------V-------HL--ERAQ	N-AKT-QN	FH-----A---N
	Chlorobium sp. KB01	076791230	------------W-E----RNE	A-E-V-E-	C-------N-----
	Chlorobium sp. N1	131355342	-------------IE---LTAQ	--T-V-E-	C-------N-----
	*Prosthecochloris sp. ZM_*	114616499	---------------R--ETDP	----V---	F-------N-----
	Prosthecochloris sp. GSB1	094083507	---------------R--ELD-	Q-----E-	W-------S-----
	*Prosthecochloris sp. CIB 2401_*	068868322	--------------ER--ETL-	--S---E-	---V----S-----
	Chlorobaculum sp. 24CR	129051262	-------------I----LLHP	--S---Q-	C--V----S-----
	Cand.Thermochlorobacteriaceae GBChlB	KER10736	------V-------HL--ERAQ	N-AKT-QN	FH-----A---N
Ignavibacteriae	*Ignavibacterium album*	385810136	--I---V-------KL-H-YQS		FY------V---N-
	Melioribacter roseus	397691543	------V------IDL---YDS		-H------V---D-
**Other Bacteria**	*Terriglobus saanensis*	320108440	----N-M---Y---RL-DEYEC		--F---I-A---DY
	Acidobacterium capsulatum	225874157	----N-M---A---RL-HEYEC		--F---W-A---DY
	Granulicella mallensis	374313410	----N-M---Y---RL-HEYDC		--F---L-A---DY
	Rubrivivax benzoatilyticus	497542852	------H---K---RL-D-YDC		FYFV--W-A---HY
	Rubrivivax gelatinosus	383759274	------H---K---RL-D-YDC		F-FV--W-A---HY
	Pseudogulbenkiania ferrooxidans	545114536	------H-VIK---EL-HSHEC		F-MV--W-A---NF
	Chromobacterium violaceum	34497152	------H-VIK---EL-HSHEC		F-MV--W-A---NF
	Sulfuricella denitrificans	543963298	------H-V-K--IKL-HEYEC		L-FV--W-A---HY
	Methylotenera versatilis	297538009	------N-V-K--IKL-HEHEC		L-FV--W-A---HY
	Methyloversatilis universalis	519008995	------H-V-K---KL-HEYPC		L-FV--W-A---NY
	Uliginosibacterium gangwonense	517434921	--I---H---K---RL-EEKEC		F-FV--W-A---AY
	Ralstonia pickettii	241662685	------H-V-K---RL-AEYPC		F-FV--W-A---HY
	Thiobacillus denitrificans	516742345	------H-V-K--LRL--EYQC		L-FV--W-A---HY
	Sideroxydans lithotrophicus	291614089	------H-V-K---QM-H-YEC		L-FV--W-A---HY
	Methylotenera mobilis	518739869	------N-V-K--IRL-HEHEC		L-FV--W-A---HY
	Methylovorus glucosetrophus	253998633	------H-V-K--TRL-HEHEC		L-FV--W-A---HY
	Ralstonia syzygii	344174282	------H-V-K---RL-AEYPC		F-FV--W-A---HY
	Ralstonia solanacearum	489361767	------H-V-K---RL-AEYPC		F-FV--W-A---HY
	Nitrosospira multiformis	82703434	------H-V-K--IEL-H-FEC		L-FV--W-A---HY
	Thauera phenylacetica	490504997	------H-V-K---KL-EEFPC		L-FV--W-A---HY
	Aquaspirillum serpens	550892077	------H---K---KL-SEHEC		F-FV--W-A----Y
	Thiobacillus thioparus	517333907	------H-V-K--LRL--EYQC		L-FV--W-A---HY
	Aromatoleum aromaticum	56478939	------H-V-K---KL-AEYPC		L-FV--W-A---AY
	Marinobacter manganoxydans	495449703	------H-V-K---KL-HEFEC		F-FV--W-A---NY
	Bermanella marisrubri	494075287	------H-V-K---KL-HEYEC		F-FV--W-A---TY
	Marinobacter nanhaiticus	490719494	------H-V-K---KL-HEFEC		F-FV--W-A---NY
	Marinobacter hydrocarbonoclasticus	387814531	------H-V-K---KL-HEYEC		F-FV--W-A---QY
	Hahella chejuensis	83635470	------H-V-K---QL-HEYEC		F-FV--W-A---HY
	Methylophaga lonarensis	497412846	------H-V-K--IKL-HEFDC		F-FV--W-A---HY
	Flexistipes sinusarabici	336323462	--I---F---Q---SL-DKYEC		FYF---W-A---GY
	Deferribacter desulfuricans	291279394	--I---F---K---KL-E-YEC		FYFV--W-A----Y
	Denitrovibrio acetiphilus	291287275	--I---F---K---DL-DKYDC		NYFV--W-A---NY
	Calditerrivibrio nitroreducens	313673527	--I---F---K---KL-DNYDC		-YF---W-A---NF
	Desulfurella acetivorans	573478392	----NLY---Q--INL-DKYKC		-YFV--W-A---GF
	Pelobacter propionicus	118580108	--I--FH-V----IRI-D-MEC		F-FV--W-S---EY
	Syntrophobacter fumaroxidans	116748129	----NLH---D--IQL-Q-YEC		F-FV--W-A---EY
	Geobacter sulfurreducens	39996932	-----FH-V-A--REL-EEYEC		F-FA--W-S---EY
	Desulfurobacterium thermolithotrophum	325295466	I---N-L---KT-LEL-DKAEC		F-F---W-AI---Y
	Thermovibrio ammonificans	319790140	I---N-F---KA-IEL-DRAES		F-F---W-AI---Y

Figure S24

Partial sequence alignment of the protein tryptophanyl-tRNA synthetase, showing a 8 aa insert (boxed) that is found in all members belonging to the order *Chlorobiales.*

Group	Species	ID	153	Insert (boxed)	204
Order *Chlorobiales*	*Chlorobium ferrooxidans*	493409794	VPVGEDQLPHVEITREIARRFNNLY	QHPIL	GEVFPEPAPKITKFSRLPGLDG
	Prosthecochloris vibrioformis	139626009	-------I---------------Q-	---GT	-S--E--E----R----V----
	Chlorobium phaeobacteroides	189501390	-----------------------Q-	---VF	-D--E--E---------A----
	Chlorobium chlorochromatii	78188056	-------I-----------K---HF	P--LY	-N--A--E------A--A----
	Chlorobium luteolum	78167834	-------I-----------K---H-	P--TK	----L--E---------V----
	Chlorobaculum parvum	193213678	-------I-----------K--SH-	---E-	-D-----------A--V----
	Chlorobium phaeoclathratiforme	194337843	-------I--------V--K---HF	P--L-	----A--E---------V----
	Chlorobaculum tepidum	21672854	-------I-----------K--SH-	---E-	-D-----------A--V----
	Chlorobium limicola	189347951	-------I-----------K--FH-	P--ET	-N-----E---------V----
	Chlorobium phaeovibrioides	145220533	-------I-----------K--SH-	P--QS	-P--V--E---------V----
	Prosthecochloris aestuarii	194335025	-------I--------------KH-	LL-DG	VA--D--E---------M----
	Prosthecochloris marina	110024017	----------------------KQ-	---E-	-D--E--E---------A----
	Chlorobaculum thiosulfatiphilum	139456449	-------I-----------K--SH-	---E-	-D-----S------A--V----
	Prosthecochloris aestuarii	012506768	-------I--------------KH-	LL-D-	VA--D--E---------M----
	Chlorobaculum limnaeum	2501894004	-------I-----------K--SH-	---T-	-D-----------A--V----
	Candidatus Chlorobium masyuteum	166807408	-------------------------	-----	----------------------
	Candidatus Thermochlorobacteri GBChlB	KER10736	----------------L-----AT-	PK-DG	NG-----EG--------V----
	Chlorobium sp. N1	131355342	-------I-----------K---H-	P--TK	----V--E---------V----
	Chlorobium sp. KB01	076791230	-------------------------	---L-	----------------------
	Chlorobaculum sp. 24CR	129051262	-------I-----------K--SH-	---A-	-D-----S------A--V----
	Prosthecochloris sp. CIB 2401	ANT65961	-------I---------------Q-	---GT	-S--E--G-----R---V----
	Prosthecochloris sp. GSB1	094083507	----------L-----L------HF	P--AT	-G--E--E-R-------A----
	Prosthecochloris sp. ZM	114616499	-------I--------------KH-	LL-DG	VA--D--E---------M----
	Cand. Thermochlorobacter aerophilum	RFM23396	----------I-----------QQ-	PK-NG	QA-----EGRL---A--V----
Ignavibacteriae	*Ignavibacterium album*	385810136	-------V--I--S---------Q-		-------E-LL---A--Q----
	Melioribacter roseus	397691543	-------V--I------------Q-		-------E-LL-S-A-------
Other Bacteria	*Paenibacillus terrigena*	517587309	I---------L-SS-Y-V----E--		-S-----QALLS--P--I----
	Paenibacillus alvei	544846112	-------V--I-Q--K-V----Q--		KP-LV--K-LVGDVP--V----
	Candidatus Kryptonium thompsoni	CUT00468	----------L----D------QT-		AP-----E-LL-E-A-------
	Marinitoga litoralis	205100913	--------Y-I-LS-----------		K------D-IV-RIPK---T--
	Candidatus Chrysopegis kryptomonas	092346731	---------------D--K---QV-		AP-----E-LL-E-A-------
	Orenia marismortui	134118996	----------I-----------Y--		D------EA-LG-VTK-L----
	Acetivibrio cellulolyticus	026073659	----------L-L---------FF-		-------Q-IL--AKV---T--
	Candidatus Kryptobacter tengchongensis	CUU06683	----------L----D--K---QV-		AP-----E-LL-E-A-------
	Hydrogenothermus marinus	170145601	----------I-L------------		K-I----KAML-EEAK-L-I--
	Persephonella atlantica	200673337	----------I-L------------		EP-----QALL-EEAK---I--
	Acetivibrio saccincola	101301108	----------L-L---------HM-		-------EAIL--AKV---T--
	Persephonella atlantica	MBK3331940	----------I-L------------		EP-----QALL-EEAK---I--
	Sporohalobacter salinus	204989152	I---------I-----------Y--		E-I----E--LGEVPK-L----
	Pelosinus propionicus	090937075	I---------L-LS---V-------		-PI----K-SLS-SAI---I--
	Oceanidesulfovibrio indonesiensis	144303480	----Q-----L-L---------H--	A	T-L----QA-L-EQAK------
	Halanaerobium hydrogeniformans	312143949	-------I--I-Q--K-V-K-----		-------KAL-SD-P--M----
	Halanaerobium saccharolyticum	491631366	-------I--I-Q--K-V-K---M-		--IL---EAL-SD-P--M----
	Acetivibrio cellulolyticus	497929432	----------L-L---------FF-		-------Q-IL--AKV---T--
	Acetohalobium arabaticum	302392435	I---------I-----------H--		--T----E--LGEVPK-L----
	Clostridium phytofermentans	160881602	----------M-M--K-V----D--		--TLH--KA-LSSC---V----
	Halobacteroides halobius	435853355	----------I----D------Y--		E------ES-LA-VP-------
	Clostridium clariflavum	374295865	----------L-L---------F-F		-------QAIL--AKV---T--
	Halothermothrix orenii	220931740	----------I-Q--R-V-K-----		AP-----EALVSDIP--V----
	Caldicellulosiruptor saccharolyticus	146295971	----------L-L---------Y--		-QI----Q-ILNTVKV-I-T--
	Persephonella marina	225850029	----------I-L------------		DQI----EALL-EE-K---I--
	Sulfurihydrogenibium azorense	225849351	----------I--------------		KP--V--KALL-EA-K-L-T--
	Thermocrinis albus	289548704	----------I-L---------H--		--T----QALL-ETP-V--T--
	Aquifex aeolicus	15606299	----------I-LS--------R--		-KI----KALL-ETPKI--T--
	Sulfurihydrogenibium yellowstonense	494812488	----------I--------------		SPI-K--QALL-ES-K-L-I--
	Spirochaeta thermophila	307719108	----K-NV----L---------F--		-----V-D-LVGEVPT-V-I--
	Geobacter daltonii	222056098	----Q--I--L-L---------Y--		-------SALL-ETPKVV----
	Geobacter bemidjiensis	197118679	----Q--I--L-----------Y--		--------ALL-ETPKVL-T--
	Pelobacter propionicus	118580108	----Q-----L-----------H--		-Q------ALL-ETPK-L----
	Desulfovibrio desulfuricans	376296380	-------I--I-Q-----H---T-F		-----I-EYRVSA-P-------
	Anaeromyxobacter dehalogenans	86158110	----V--V--L-LS---V-K--FH-		-------Q-LL-AAPKIL-T--
	Mucispirillum schaedleri	555550898	----V--V----LA---V-----F-		--I-V--ESML-NVPK------
	Trichoplax adhaerens	196018955	----------I-Q--R-V-K-----		AP-----EALVSDIP--V----
	Mesotoga prima	389844710	-------IY---L------K---QF		A------E-ILA-VPK---T--
	Roseiflexus castenholzii	156742130	----K--AS-L-V---------T--		---L---ETL-GDVPT-V-I--
	Dethiosulfovibrio peptidovorans	491903638	-------SA-L------------F-		-------DILL-PTPKV--T--
	Slackia exigua	493405898	----K-----I-V--Q------KR-		-K-----EGILSDAIEI-----
	Acetonema longum	490199933	----D-----M-QA-K-V----D--		--TLKV-KELLSATP-------
	Dehalogenimonas lykanthroporepellens	300088125	I---------L-LA---V----DHF		-P-----QG-L-N-PMVV----

Figure S25

Partial sequence alignment of the protein tryptophanyl-tRNA synthetase, showing a 5 aa insert (boxed) that is found in all members belonging to the order *Chlorobiales.*

Group	Species	Accession	31	Insert	87
Order *Chlorobiales*	*Chloroherpeton thalassium*	193216377	YGTNVVAGVTPGKGGMQYNGNEKDPF	IREVPIF	NTVKDAVKKAEANTSVIFVPAAFA
	Chlorobium phaeovibrioides	145220333	---------------VL----DR-S-	C-P--V-	----E--E------T---------
	Chlorobium chlorochromatii	78188444	---------------TL-H--D--K-	C-P--V-	---R---E-T---V-------P--
	Chlorobium limicola	189345876	---------------IL-H--D--R-	C-P--V-	----E--E-T-----------P--
	Chlorobium luteolum	78187602	---------------VL-H--DR-K-	C-P--V-	-------E-----AT---------
	Chlorobium phaeobacteroides	119356239	---------------TI-H---R-H-	C-P--V-	-------D-----AT------P--
	Chlorobium phaeoclathratiforme	194335584	---------------IL-H-----K-	C-P--V-	D--RE--Q-----AT---------
	Chlorobaculum parvum	193213404	---------------IL-------Q-	C-P--V-	D--RE--E-----AT---------
	Chlorobaculum thiosulfatiphilum	139456761	---------------IL-------Q-	C-P--V-	D--Q---D-----AT------P-
	Prosthecochloris marina	110022769	---------------L--T--DR-T-	TSS--VY	-------EAT---A-------P--
	Prosthecochloris aestuarii	194333247	---------------L--------A-	C-P--VY	-------EE-G--A-------P--
	Chlorobaculum limnaeum	2501893444	---------------IL-------Q-	C-P--V-	D--Q---D-----AT------P--
	Chlorobium ferrooxidans	493411426	---------------VL-H-----K-	C-P--V-	---R---E-----AT---------
	Chlorobaculum tepidum	21673108	---------------IL-------Q-	C-P--V-	D--QE--D-----AT------P--
	Prosthecochloris ethylica	175187487	----------------E-------R-	C-P--V-	---RE--EE-G----L--------
	Candidatus Chlorobium masyuteum	166807707	---------------VL-H-----K-	C-PV-V-	---R---E-----AT---------
	Cand. Thermochlorobacter aerophilum	RFM23093	L-----------H-A-----R-	------Y	---RE--AAQRV---I-Y---P--
	Chlorobium sp. 445	PI048084	L-----------Y-A-----R-	-----VY	---RE--AAQKV---I-Y---P--
	Chlorobium sp. N1	131354450	---------------VM----DR-K-	C-P--V-	---RE--L-----AT---------
	Chlorobium sp. KB01	076791543	---------------VL-H-----K-	C-P--V-	---RD--E-----AT---------
	Prosthecochloris sp. ZM_2	114608810	----------------E-------R-	C-P--V-	---RE--EE-G----L--------
	Prosthecochloris sp. HL-130-GSB	085659004	----------------E-T---R-S-	C-P--V-	D--R---EQ----A-------P--
	Chlorobaculum sp. 24CR	129050450	---------------IL-------Q-	C-P--V-	D--QE--D------AT-----P-
	Prosthecochloris sp. GSB1	094081941	---------------L--G--DR-G-	C-P--VY	----E--EE-G--A-------P--
Larger *Ignavibacteriae* Clade	*Chlorobi bacterium OLB6*	KXK34392	--S------------SM-S-KGD-T-	V-PL-VY	---RE--TATG--V-I--------
	Chlorobi bacterium OLB7	KXK56074	---------------TE-HPA-FIRA	--V-	---SE--AATG--A-------P--
	Chlorobi bacterium OLB5	KXK40992	---------------TLFDEKIPV-		--C-E--E--G---------P---
	Ignavibacterium album	385810678	---K-----------TKFE-IPIF		---EE---EQK---------P---
	Melioribacter roseus	397691702	---K----------QEFEDTGIPI-		----E---ETK-TA-A----P---
Other Bacteria	*Croceibacter atlanticus HTCC25*	298208207	------G--------QTHLDKPVF		---DE---E-K-D--I----P---
	Rhodothermus marinus	268318044	---------------QKHLDRPVF		---AE--E-EG----I----P---
	Flavobacterium limnosediminis	559216499	------G--------T-HLDRPVF		-------EQ-G-D--I----P---
	Riemerella anatipestifer	313206103	------G--------TEHL-KPVF		---A---E--D--V-I----P---
	Myroides injenensis	497939601	------G--------TMHLDRPVF		---AE--E--G-D--I----P---
	Riemerella anatipestifer	386321909	------G--------TEHL-KPVF		---A---E--G--V-I----P---
	Flavobacterium cauense	559194736	------G--------T-HLDRPVF		-------DQ-G-D--I----P---
	Elizabethkingia meningoseptica	510824792	------G--------SEHL-KPVF		---AE--E--G--V-I----P---
	Echinicola vietnamensis	431795425	---K--G--------STHL-KPVF		-S-AE---AT--D--I----P---
	Leadbetterella byssophila	312130295	------G--------QTHLDRPVF		---EE---ETG-DV-I----PG--
	Cecembia lonarensis	496475462	------G--------TKHLEKPVF		-S-EE--A-TG----I----P---
	Myroides odoratimimus	493301313	------G--------TTHLDRPVF		---SE--E--G-D--I----P---
	Chryseobacterium indologenes	573452557	------G--------SEHL-KPVF		---A---E--G--V-I----P---
	Bergeyella zoohelcum	488739944	------G--------TEHL-KPVF		---A----Q-G-DV-I----P---
	Chryseobacterium gleum	489067148	------G--------SEHL-KPVF		---A---E--G--V-I----P---
	Marivirga tractuosa	313675255	------G--------QKHLDRPVF		---E----E-G--V-I----P---
	Pontibacter roseus	517290593	------G--------NNHLDLPVF		---EE---RTG-DV-I----P---
	Riemerella columbina	517505678	------G--------SEHL-KPVF		---AE--E--G--V-I----P---
	Fluviicola taffensis	327405863	------G--------SSHLDRPVF		---A---A-TG-DV-I----P---
	Saprospira grandis	488735375	---------------TSHLDRPVF		---E----ETG-DV-I----P---
	Polaribacter franzmannii	517773502	------G--------QEHL-KPVF		---VES-E-VG-D--I----P---
	Spirosoma panaciterrae	522085613	------G--------QTHLDRPVF		---QE--D--G--V-I----P---
	Fibrisoma limi	496584386	------G--------QTHLDRPVF		---QQ--DE-G-DV-I----P---
	Fibrella aestuarina	436835319	------G--------QTHLDKPVF		---YE--E--G-D--I----P---
	Cyclobacterium marinum	343082734	------G--------STHL-KPVF		---E----TKG-D-TI----P---
	Spirosoma linguale	284038109	------G--------QTHLDRPVF		---QQ--DQ-G--V-I----P---
	Gracilimonas tropica	521069933	------G--------QTHLDRPVF		---A----EVQ---------P---
	Muricauda ruestringensis	344204408	------G--------QEHL-KPVF		---E----EVG-D-TI----P---
	Spirosoma spitsbergense	522091410	------G--------QTHLDRPVF		---QQ--DQ-G--V-I----P---
	Empedobacter brevis	518819263	---Q--G--------QTHL-LPIF		-------DQ-G-DV-I----P---
	Desulfobacca acetoxidans	328953555	---K-----------QKMDDVPVF		----E--AA-G----L----P---
	Bacillus cereus	446037332	---KI-G--------TEIE-VPVF		---E----ATG--A--VY--P---
	Thermocrinis albus	289548927	---Q-----------QKVEDIPVF		---DE---ETQ--C-L----P---
	Archaeoglobus fulgidus	11499767	---KI----------TEVL-VPVY		DS--E--RE-D--A-------P--
	Archaeoglobus sulfaticallidus	488601692	----I-G--------EVH-IPVF		----E--EETS--A------P---
	Arenimonas oryziterrae	551350688	---K--G--------TTHI-LPVF		---A---NETG-DA---Y--PP--
	Clostridium ultunense	491759884	---KI-G-TS-----TELE-IPVF		D--AE--A-TG--A-I-Y--P---

Figure S26

Partial sequence alignment of the protein succinyl-CoA synthetase subunit alpha, showing a 7 aa insert (boxed) that is found in all members belonging to the order *Chlorobiales.*

Group	Organism	Accession	2		48
Order *Chlorobiales*	*Chlorobaculum tepidum*	21673819	RIGVAGVGKLGEFHTNLLKQI	AAEA	SDVEFSGVFDLNPERLQEIGKK
	Chlorobaculum parvum	193212872	---------------------	---N	P---L-----------------
	Chlorobium phaeoclathratiforme	194336309	K---I----------K--TE-	-Q-R	P--HCA-L---DSN-AE-MAR-
	Chlorobium phaeobacteroides	119356931	----I-T--------K--GE-	-QNR	H-T-LA-I----RI-AE---H-
	Chlorobium phaeovibrioides	145219610	---II----------K--AE-	-N-Q	PA--CA------TA-AE-MAA-
	Chlorobium luteolum	78187012	---I----------K--AE-	-Q-T	PG-TCA-----D-A-AR--AS-
	Chlorobium limicola	189346519	----I-T--------K--RD-	-L-S	P--HLA-IY-KSSL-AE--AH-
	Chlorobium chlorochromatii	78188699	K---I----------K--TEL	-H-R	T-LHVA-I----TQ-AE-MAQ-
	Chlorobaculum thiosulfatiphilum	139455688	---------------------	-G-S	P---------------------
	Chlorobium ferrooxidans	493411370	Q---I----------K--TE-	-R-L	P--TCA-I---DRN-AD-MAAR
	Chloroherpeton thalassium	193216278	-A-I----------K---E-	C--Q	KN-HLT------QT-AA--A--
	Chlorobaculum limnaeum	2501894514	---------------------	---S	Q----A----------------
	Candidatus Chlorobium masyuteum	166808114	Q---I----------K--TE-	-R-L	P--TCA-I---DRN-AD-MAAR
	Cand. Thermochlorobacter aerophilum	RFM23578	---IV-------L-AK--SEL	SL-R	Q-TA-I----I-A--CA-VA--
	Candidatus Thermochlorobacteri GBChlB	KER10002	-LA-V-------L-AK--SEL	VR-R	N-AR-V----I-GA-AD--ARR
	Chlorobium sp. KB01	076791136	Q---I----------K--TE-	-R-L	P--TCT-I---DRN-AD-MAAR
	Chlorobium sp. 445	PI049003	---IV-------L-AK--REL	SL-R	K-I—V-----CKET-CKDVAR-
	Chlorobium sp. N1	131357401	K---I----------K--AE-	-R-Q	PA-TCA-----D
Larger *Ignavibacteriae* Clade	*Chlorobi bacterium OLB4*	KXK01619	K-SII-L-H--KI-LKI-SEL	SN-R	K-FSI---Y-IDN-K-SGIS
	Ignavibacterium album	385809634	KV--I---H--KL--KMF-S-		ENC-LI--Y-A-EQTAKSVAQE
	Melioribacter roseus	397691384	K---I-T-H--RI-VK---EL		DN--LC--Y-KDF-KSKNVSEE
Other Bacteria	*Candidatus Hydrothermae bacter*	MBP7702301	KF-IV---H---I-VRN--S-	V--T	-QI-LT-I--V-LA-A---SER
	Leptospira terpstrae	489062375	-L--I-T-HM-QY-V-VA--L		A-A-LI-I--A-L--ATQ-AE-
	Leptospira wolffii	514354638	K---I-T-HM-QY-V-VA-TL		--A-LI-I--SDS--ATQ-AE-
	Brachyspira pilosicoli	404475256	-SLI---RM-Q--L-VIN--		NSINLK-IY-ADENH-N-VSN-
	Brachyspira hampsonii	495999332	-SLI---RM-Q--L-VVS--		NQINL--IY-ADENH-N--S--
	Opitutus terrae	182413903	-C------S--QH-ARIYSTL		PN---A-I-ETSDA-AA--CA-
	Nitrospina gracilis	491147715	-A----I--M-QY-VGV-SEM		R-IKLTHIA-V-ED-CR--SNR
	Ureibacillus thermosphaericus	515282761	-V--V-T--M-F--CSK---M		KN-N-I-IY-TDMN-AKK-S-A
	Bacillus massiliosenegalensis	517982370	KV--I-T--M--N-LKNYIE-	S	DQC-VV-IY-V-ER-AS-LAS-
	Acidithiobacillus caldus	340781596	-TA-V---H--R--AQKYAAC		SNLQ----QD---AAAVAAE
	Kordia algicida	494153791	KA--L-A-H--KI-LR--Q-S		-KY-LI-FY-A---YA-K-V-E
	Trichodesmium erythraeum	113475581	-V--I---NM-QH--RV-SLL		K-T-LV--A-I-V--GLDTAS-
	Oscillatoria formosa	518317616	----I---NM-QH--RV-SLL		K---LV--S-I-V--GIDVAS-
	Microcoleus vaginatus	493685732	----I---NM-QH--RV-SLL		K-I-LV--A-I-V--GIDVAS-
	Dactylococcopsis salina	428779731	-V--I---NM-QH--RV-GLL		K---LY--A-I-VT-GLD-AS-
	Cylindrospermum stagnale	434404788	----I---NM-QH--RV-SSM		K---LV--S-I-V--GL-TAS-
	Anabaena variabilis	75908580	-V--I---NM-QH--RV-SSM		K---LV--S-I-V--GL-TAS-
	Raphidiopsis brookii	496702689	-M--I---NM-QH-AR--SSM		K---LV--S-I-V--GI-TASR
	Scytonema hofmanni	516355105	----I---NM-QH-ARI-SSM		K---LV--A-I-V--GL-TAS-
	Peribacillus alkalitolerans	163100509	----V-T--M-TY-CSK---M		KN-D-V----KDLK-AE--SR-
	Candidatus Nitronauta litoralis	QPJ61148	----I----M--Y-VGV-SEQ		-AA-LTL-A-N-EQ-A-----R
	Leptonema illini	KAB2929387	KV--I-T-HM-QY-V-VIVN-		AQA-VT-C--AD-N--A--S-R
	Pelagicoccus mobilis	200355414	K C------Y--QH-ARIYSEL		EN--LA-I-ET-T--AA--SE-
	Ruminiclostridium cellobioparum	004630087	KAAII-F-GM-S--AQQI---		NSI-IT--Y-I--QKV--GV--
	Brachyspira alvinipulli	028330838	-SLI---RM-Q--L-VIN--		NQINL--IY-E-KNH-D--SQ-
	Calothrix brevissima	096642204	R ---I---NM-QH--RV-SSM		K---LV--S-I-V--GL-TAS-
	Anabaenopsis elenkinii	200988229	R V--I---NM-QH-ARI-SSM		K----V--S-I-V--GL-TAS-
	Nitrospina gracilis	005006113	-A----I--M-QY-VGV-SEM		R-IKLTHIA-V-ED-CR--SNR
	Thermodesulfobacterium commune	038060646	-V-II-I-H--R--AEK-A--		DKADLVALV-I----V--AV--
	Leptospira ryugenii	108977756	-L--I-T-HM-QY-V-VA-ML		--ATLE-I--AST--ASQ-AE-
	Cuspidothrix issatschenkoi	104386150	R ---I---NM-QH--RV-SSM		K---LV--S-I-V--GL-TAS-
	Cylindrospermum stagnale	015208246	R ---I---NM-QH--RV-SSM		K---LV--S-I-V--GL-TAS-
	Brachyspira hampsonii	008728832	-SLI---RM-Q--L-VVS--		NQINL--IY-ADENH-N--S--

Figure S27

Partial sequence alignment of the protein Gfo/Idh/MocA family oxidoreductase, showing a 4 aa insert (boxed) that is found in all members belonging to the order *Chlorobiales.*

Group	Species	Accession	209	Insert	249
Order *Chlorobiales*	*Chlorobaculum tepidum*	21673819	ANVTASRLSRSKLRKMRFFTRN	PK	SYASLDFTSGKSEVFRL
	Chlorobaculum parvum	193212872	---------------L---S-K	--	-------N---------
	Chlorobium chlorochromatii	78188699	------------M--L---CTE	--	------L------IY--
	Chlorobium ferrooxidans	493411370	-----------RM--L---GT-	--	------L-T--------
	Chlorobium limicola	189346519	----------NRY------CNE	--	------L----------
	Chlorobium phaeoclathratiforme	194336309	----------NRM--L---CND	--	------L-T--------
	Chlorobium phaeobacteroides	119356931	----------KSM--L---CND	--	------L-T--------
	Chlorobium phaeovibrioides	145219610	----------CRQ--L---GTD	--	------L-T--------
	Chlorobium luteolum	78187012	-----------RS--L---GTA	--	------L-T--------
	Chloroherpeton thalassium	193216278	---S---I--T-S--V---SK-	-N	------L-N----I--I
	Chlorobaculum limnaeum	2501894514	-------------------S--	--	-----------------
	Chlorobaculum thiosulfatiphilum	139455688	-------------------S--	--	-----------------
	Candidatus Chlorobium masyuteum	166808114	-----------RM--L---GT-	--	------L-T--------
	Cand. Thermochlorobacter aerophilum	RFM23578	-T-----I--TRV------CK-	-P	------LM---------
	Cand. Thermochlorobacteriaceae GBChlB	KER10002	-T-----I--T-V----L-CAD	-N	------L-T--A-----
	Chlorobium sp. 445	PI049003	-T-----I--TRV------CK-	-Q	------LIT--------
	Chlorobaculum sp. 24CR	129051696	-------------------S-K	--	-----------------
	Chlorobium sp. KB01	076791136	-----------RM--L---GT-	--	------L-T--------
	Chlorobium sp. N1	131357401	-----------RS--L---GT-	--	------L-T--------
Larger *Ignavibacteriae* Clade	*Chlorobi bacterium OLB4*	KXK01619	-------I-Q--M----I-QK-		A-I-I--VDNST-I-S-
	Chlorobi bacterium OLB7	KXK54677	--I----I-QRPM--L-M-Q-D		--I----AKPSV-I--I
	Chlorobi bacterium NICIL-2	KXB98010	--L-----TPKP---L-L-QPM		A-M----SVPDL--Y-I
	Chlorobi bacterium OLB6	KXK35631	--L----I-AKPM--L-L-QNE		--I-I-LAQSTA-M---
	Ignavibacterium album	385809634	-------I-QK-M----I-QKD		N-L----VT-V---Y--
	Melioribacter roseus	397691384	-------I-QKRM----M-QQD		T-F-V--IE-S--II--
Other Bacteria	*Niastella koreensis*	375147951	--L-S--I-MK-M----V-QKD		A-IGI--LNK---IIK-
	Empedobacter brevis	518819185	V--ST--M-MKNM----V-Q--		A-I-I--LAK-T-IIKM
	Haliscomenobacter hydrossis	332663296	--L----I-MKQM--V---Q-D		A-I----LEKNAQ-V--
	Lewinella persica	522055502	--L----I-LKNM--V-L-QPD		A-I---LLEKE-QIV--
	Gramella forsetii	120435026	--L----I-MKNM--S---Q-D		A-I-V--LEK-C--VKM
	Leeuwenhoekiella blandensis	497466201	--L----I-MK-M--A---Q-D		A-I-V--LEK-C--V-M
	Formosa agariphila	537742421	--L----I-LKSM--T---Q-D		A-I-V--LEK-C--VKM
	Chitinophaga pinensis	256420576	--L-S--I-LK-M----L-QKD		A-IGI--LDK-T-IIK-
	Weeksella virosa	325954811	V-IST--M-LKNM----V-Q--		A-I-I--LNK-T-IIKM
	Flavobacterium rivuli	519055416	--L----I-MKNM--S---QKD		A-I-V--LDKVC--VKM
	Candidatus Chrysopegis kryptomonas	092348819	-------I--KNE----I-QK-		A-I-I--LQ-IA-I---
	Candidatus Kryptobacter tengchongensis	072149809	-------I--KNE----I-Q-D		A-I-I--LQ-VA-I---
	Candidatus Kryptonium thompsonii	047133254	--I----I--KNE----I-QKD		A-I-I--LQ-IT-I---
	Caldithrix abyssi	HED11325	-------I-AK-M----V-QK-		A-I-F--SD-EA-A-
	Caldithrix abyssi	HHJ53862	--L----I-AK-M----L-QKD		A-I-M--ND-L--I-Y-
	Nonlabens xiamenensis	124980683	--L----I-LK-M--A---Q-D		A-I-V--L-K-V--V-M
	Leeuwenhoekiella blandensis	009780399	--L----I-MK-M--A---Q-D		A-I-V--LEK-C--V-M
	Leeuwenhoekiella nanhaiensis	099646996	--L----I-MK-M--A---Q-D		A-I-V--LEK-C--V-M
	Taibaiella lutea	150030949	--L----I-LK-M----L-Q-D		A-IGI--LDK-T-II--
	Gemmatimonas groenlandica	171224539	--I----V--ER---L-I-Q-S		G-I---LAA-TG-F---
	Luteitalea pratensis	AMY11198	-------I--DRV--V--IEPA		A-V-V-YA-REL--W--
	Owenweeksia hongkongensis	375011569	--L----I-LKNM--T-I-Q-D		A-VTI--LEKS---I-M
	Zunongwangia profunda	295135065	--L----I-MKNM--S---Q-D		A-I-V--LEK-C--VKM
	Capnocytophaga cynodegmi	517090214	--L----I-LKNM--S---QKD		A-I-V--LYK-V--V-M
	Psychroflexus tropicus	517867825	--L----I-LKNM--S---Q-D		A-V-V--LEKHT--VKM
	Bizionia argentinensis	495914711	--L----I-LKNM--T---Q-D		A-I-V--LEK-C--VKM
	Flavobacterium frigoris	494229777	--L----I-MKNM--T---QKD		A-I-V--LEK-C--VKM
	Lewinella cohaerens	522022900	--L----I-LKQM--V---QKD		A-I---LLEKQAQLI--
	Niabella soli	495859222	--L-S--I-MK-M----L-QKD		A-IGI--LNK-T-IIK-
	Zhouia amylolytica	568804158	--L----I-LKNM--S---Q-D		A-I-I--LEK-V--VKM
	Bergeyella zoohelcum	488740381	--L-T--I-MKAM--S---QKD		A-I-V--LEK-A--I-M
	Elizabethkingia meningoseptica	510825000	--L-T--I-MKAM--S---QKD		A-I-I--LEK-A--I-M
	Nonlabens dokdonensis	443244512	--L----I-LK-M--A---QKD		A-I-V--LTK-V--V-M
	Polaribacter franzmannii	517771826	--L----I-MKNM--T---QKD		A-I-VN-L-KE---V-M
	Croceibacter atlanticus	298208473	--L----I-LKNM--A---QKD		A-I-V--LTK-C--VKM
	Riemerella anatipestifer	407452326	--L-T--I-MKAM--S---QKD		A-I-V--LEK-A--I-M
	Ornithobacterium rhinotracheale	392389643	--I-T--I-MKNM----I-QKD		A-I-V--LEK---II-M
	Caldithrix abyssi	493985549	--L----I-AK-M----I-QKD		A-I-M--NE-Y--I-Y-
	Singulisphaera acidiphila	430746299	--L----A-FQAM----LWSAE		G--M---MTKQGTLV-K
	Marinimicrobia bacterium	551209323	--L----V-AKQM-R--I-Q-D		A-F-I--LKNST-IY--
	Nitrosococcus oceani	77165232	-------V-MKAQ----V-QQD		A-I-I--QDKVLS-Y-K
	Thioalkalivibrio thiocyanodenitrificans	517042302	----S--V-MKTE----I-QQ-		A-I-V--QNRGLA-H-V
	Nitrosococcus halophilus	292491337	-------V-MKAQ----I-QQD		A-I-I--QDKILSIY-K

Figure S28

Partial sequence alignment of the protein Gfo/Idh/MocA family oxidoreductase, showing a 2 aa insert (boxed) that is found in all members belonging to the order *Chlorobiales.*

Group	Species	Accession	176	Insert (boxed)	223
Order *Chlorobiales*	*Chlorobaculum parvum*	193213033	GQSLHGDEVEVLVTKVPET	YVSKQSP	HQRCEGLIVNVVSRKLTTIVGT
	Chlorobium tepidum	21673344	-----L-Q---Q------S	-A-R---	-------V---IE-R-V-V---
	Chlorobium phaeobacteroides	119357755	NS-M-------Q-------	-A--ST-	------IVKS--K-RV------
	Prosthecochloris aestuarii	194333503	SDAI---D---------Q-	-QTQ---	---------K------RSV---
	Chlorobium limicola	189346025	NSAI----I--V-------	-SA-ST-	------IV----T--ISA----
	Chlorobium phaeovibrioides	145219389	NSAI---------A---SS	-S-RST-	--------T-IIN-RI------
	Chlorobium luteolum	78186405	NSAI------LT-----K-	-S-RST-	------MVTKIIQ--VS-----
	Pelodictyon phaeoclathratiforme	194336956	-L-I---------S---D-	-S--ST-	------AVQK-LT-RI------
	Chlorobium ferrooxidans	493409164	NF-I------------D-	-S--ST-	-------VQK-I--RI------
	Chlorobium chlorochromatii	78188726	-AA---------SST---	-TA-A--	------VVQR-IN-H--M----
	Chloroherpeton thalassium	193216324	NGA-D--I---R--S--S-	-S-RST-	-E----V-TQ-QK-VRQEF--V
	Chlorobaculum limnaeum	2501894184	-EA--L-------------	---R---	-------V--------------
	Prosthecochloris marina	110024153	-DAI-------------K-	-Q-Q-T-	--------TK-IN-A-H-----
	Prosthecochloris ethylica	198911867	AI-R---D-------P-	-Q-Q---	-------VTA-RQ-N--SVI--
	Chlorobaculum thiosulfatiphilum	170181222	--A--L-QI----------	---R---	-------V---IE-R-A-V---
	Candidatus Chlorobium masyuteum	166807966	-I-------------D-	-S--ST-	-------VQR-I--RI------
	Chlorobium sp. N1	131358104	AI------LT-----K-	-STRST-	------A-TRIIQ--VSV----
	Chlorobium sp. KB01	076791152	-I-------------D-	-S--ST-	-------VQK-I--RI------
	Chlorobaculum sp. 24CR	167599335	-----------------	-------	-------V----------V---
	Prosthecochloris sp. ZM	114617576	AI---D---------Q-	-QTQ---	----------------RSV---
	Prosthecochloris sp. ZM_2	114608494	AI-R---D-------P-	-Q-Q---	-------VTA-RQ-NM-SVI--
	Prosthecochloris sp. HL-130-GSB	085659969	AI-K---Q-------P-	-Q-Q---	------VVTA-IE-RIQS----
	Prosthecochloris sp. GSB1	094082062	-G-V-------V--T--K-	-R-QVT-	-------V-K--N-S-HSV---
Ignavibacteriae	*Ignavibacterium album*	385809169	-TAFD--K---ILFAKQKG		RNL--Q----L---RKEY--I
Other Bacteria	*Coprococcus comes*	547810973	NGAMQ------VI--A--G		K-R--K-TKI-Q-GTQR---Y
	Dorea formicigenerans	491476797	-GA-D--T---II--ASDR		DGK-R--K--KI-E-G---V--L
	Ruminococcus gnavus	547306355	NGAFQ-----F-I--S--G		R-K--K--R---HGT-KVI-L
	Ruminococcus lactaris	491807899	NGAFQ-----CII-GS-SG		R-Q--K--GII-HQV-KV--L
	Brevibacillus borstelensis	489487643	NGA----T-L-RME-ISGG		N-A--R-IR--E-GI-EV---
	Bacillus methanolicus	489446679	NNAM---T-L-RISTESSG		--R--TV-R-IE-GTQQ----
	Brevibacillus brevis	515820898	NGA----T-F-R-E-EAGG		N-L--Q-IRI-E-GIKQV---
	Capnocytophaga granulosa	512458271	NKA----L---Y-FPRRRE		KL--E-TQ-IE--R--F--L
	Gillisia limnaea	494046916	NKA-N-----IYIYNKRNK		RKS--EV-KIIK--R-EF--I
	Salinibacter ruber	83815922	-EA----L-R-A-AARAPE		DKKR-CEVLE--E-RC-EV---
	Capnocytophaga gingivalis	488743213	NKA----V---Y-FPRRRE		KL--E-TQIIE--R--F--V
	Pedobacter heparinus	255531532	HNA----K-K-YIYAKKSG		RKN--EV-EIIE-SK-DFI-V
	Mucilaginibacter paludis	495785702	RNA----R-K-Y-YAKSKG		K-K--EVIEIIK-AKMEFT-I
	Joostella marina	495886911	NKA----L---Y-FPRRRK		GKKI--E-TKILE--K--F--I
	Muricauda ruestringensis	344202636	NRAF---T---FIKPKRKS		KKM--EVSK-LE--K-SY--I
	Veillonella parvula	544718202	-TAM-N-K-R-RIVPSDY-		KHKR--I--D-IE-ANE-----
	Dorea formicigenerans	117980906	-GA-D--T---II--ASD		RDGK-R--K--KI-E-G---V--
	Candidatus Blautia avicola	HJD28867	-GAF---Q-QI-LK-EER		-GK-R--RV-KILE-GMEE---
	Veillonella dispar	MBF1741846	-TAM-N-K-R-R-IPSNY		TKHKR--I----IE-ANE-V--
	Blautia faecis	148463538	-TAM-Q-K-R-II--E-Q		EGK-R--IV-K-LE-GM-Q---
	Hespellia stercorisuis	084533899	AFQ--K---I---S--		ER-K--K--KILE-GT--L--
	Zhouia amylolytica	038268184	KA--------Y-FPRRR		GSK-L--E-TK-IN--R--F--
	Veillonella dispar	566235315	-TAM-N-K-R-RIVPSDY-		KHKR--I--D-IE-ANE-----

Figure S29

Partial sequence alignment of the protein ribonuclease R, showing a 7-8 aa insert (boxed) that is found in all members belonging to the order *Chlorobiales.*

			558		608
**Order *Chlorobiales***	*Prosthecochloris aestuarii*	194333503	KVAILANFVKKIGYTLQLTGKG	KNGAP	VVKAKALRDLLKQVHGTNVEFLVN
	Chlorobium phaeobacteroides	189499728	-----T---RR-----K-D--A	-G---	K-SSQ---N---------------
	Chlorobaculum parvum	193213033	R-QV-----R---FD-K-DR--	-DS A	T-SSR---E--QK-----------
	Chlorobaculum tepidum	21673344	--QV--S--R---FD-K-DR--	-DS A	T-SS----E--QK-R---------
	Chloroherpeton thalassium	193216324	RIVQ-SE--T-L-FK-E-KKNA	L-N -	T-SS-G--S--E--K-S---I---
	Chlorobium luteolum	78186405	R-I----------D-KIN --	-D- -	I-S--S--E--QK-----------
	Chlorobium phaeovibrioides	145219389	R-T--------M--E-K-N --	-D- -	I-S-----E--QK-R-S-------
	Chlorobium limicola	189346025	R-LV-----R------KIN RE	-T- -	I-S-----Q--Q----S-------
	Chlorobium ferrooxidans	493409164	--I--S----RF-FD-K-N R-	-E- -	I-T-----Q--Q--K--------S
	Prosthecochloris marina	110024153	--G------R----S---N---	-G-V-	KISS----------------M--
	Chlorobaculum thiosulfatiphilum	TNJ39330	R-TV-----R---FD-K-DR--	-ES-	T-S-----E--QK-----------
	Prosthecochloris ethylica	198911867	—-G------R----Q---D--S	-----	N-TS---QG-------S-------
	Chlorobium phaeoclathratiforme	194336956	--L-------RL-FD-K-N R-	-E- -	I-S-S---Q--Q--K-S-I----S
	Chlorobium chlorochromatii	78188726	--VMV-----RL--D-KIN RS	-E-I	I-S-QS--Q--Q--K--------S
	Chlorobaculum limnaeum	2501894184	R-TV-----R---FE-K-DR--	-ES-	T-SS-S--E--QK-----------
	Cand. Thermochlorobacter aerophilum	RFM24073	-IKT--A--RL--E-E-KKNA	F-N E	TATSQ---K--AD-K--S--Y--S
	Chlorobium sp. N1	131358104	R-IV----------E-K-NKGK	EGP	I-T-----E--QK-----------
	Prosthecochloris sp. ZM	114617576	----------------------	-----	------------------------
	*Prosthecochloris sp. GSB1_*	094082062	--G------R----N---D--S	-K-G-	T-TS--------------------
	Prosthecochloris sp. ZM_2	114608494	--G------R----Q---D--S	-----	N-TS---QG-------S-------
	Chlorobaculum parvum	HHE33015	R-QV---------FD-K-DR--	-ES-	T-SS----E--QK-----------
	Chlorobaculum sp. 24CR	RXK82182	R-QV-S---R---FD-K-DRR-	-ES-	T-SSR---E--QK-R---------
	Chlorobium sp. KB01	076791152	—-I--S----RF-FD-K-NRGK	EG P	I-T-----Q--Q--K--------S
Ignavibacteriae	*Ignavibacterium album*	385809169	-IMEFVR---SL--QVSQNLIK		-TSEFQK--D--K-KEE-P-I-
	Melioribacter roseus	397690865	-INEF-R---SL--SFDPNAAN		-PMEFQK--DAAK-SEE-AVI-
**Other Bacteria**	*Rhodothermus marinus*	345303465	-IRQ--QY-RVF--H-E--ED-		T-SS---NE--QH-K--PE-PVIE
	Rhodonellum psychrophilum	518430380	RLGTF---A--F-HEVKIDDEQ		KVS---NK--DEIQ-KPEQNVLE
	Porphyromonas levii	517169631	-L-D--E--GRL--K-RT--TP		EMI----N---EK---KPEAE-IE
	Marinilabilia salmonicolor	498351409	-YESFSK--R-F-FEAAPKQSE		TISSS-NRV-GE-Q-KKQQNI-E
	Dysgonomonas gadei	493853178	-MEN-SE-IRRF--KIKVD-SK		TNVS-SINH--DE-S-KPE-N-IS
	Dysgonomonas mossii	493896130	-MKN-SE-IRRF--KIKVD-TK		TNVS--INH--DE-S-KPE-N-IS
	Gracilimonas tropica	521070004	-L-GI-EQ--P--IKFDVEKNI		SP-KIN---GK-EN-SL-YI--
	Candidatus Azobacteroides	212550560	-IKN-KQL-RSF--K-NIKRIN		NETS-NISS-F---Q--REQN-IE
	Belliella baltica	390945305	RLETFS--A--F-HEIKVGEGN		RIS---NQ-MDEIQ-KPEQNVLE
	Joostella marina	495886911	-LML-QGVIS-F--SIDMKS-K		AVTSS-NA--EE-Q--KEQN--D
	Thermophagus xiamenensis	498212010	-FETFSR--R-F-LEAKPK-QE		TISHS-NR--DK-Q-QKHQNI-E
	Mariniradius saccharolyticus	495907092	RLETF-T-A--F-HD-KISEGA		KIS---NQ-MDEIV-KPEQNVLE
	Echinicola vietnamensis	431796052	RLETF---A--F-HEVSI-EAT		RVSAT-NK-MGEIE-KPEQN-LE
	Emticicia oligotrophica	408673906	-LQNF---AGRF-FSIRTDSDK		SLSNSMNKMMSE-E--P-QNVLE
	Anaerophaga thermohalophila	515148300	-YETFSK--R-L-FEAMPK-RE		TISHS-NR--DE-Q-KKQQNI-E
	Ornithobacterium rhinotracheale	392390324	-L-S-KQ-IHQF--K-E-GDRK		KTTASINQ--HD-K-KGE-NMIE
	Alistipes indistinctus	496424899	-L-Q-RS--MRF--QMKSDAV-		KAL--DINK-MTN---KQE-N-IS
	Porphyromonas bennonis	516799870	RLQNF-EY-GRL--KFIY--DA		QSISDN-NK--EEIK-KPE-NVIQ
	Owenweeksia hongkongensis	375012907	RLMD-S----QF--QVDTKNRN		GISRS-NKM-SD-K-KGEANMIE
	Dyadobacter beijingensis	518787359	RLQTF----A-L-LK-EVEEES		KI--SMNAM-AK-E-KPEQN-IE
	Balneola vulgaris	516842897	-L-GI-GQ-APL-IKFEVRD-I		TP-YINN--QK-KE-P--MI--
	Muricauda ruestringensis	344202636	-LMA-NGVISRF-HSIN-KDPK		SINQS-NK--ED-K-KKEQN--D
	Halanaerobium hydrogeniformans	312143213	RMIQFKE-IHNF--E-KGIKN-		-HPRS-QAI-EE-R-SKE-KIIE
	Dorea formicigenerans	491479248	-IRA--T-INNF-FSMHIGANE		-RP-EIQK--GK-E--PQ-A-IS
	Peptoniphilus rhinitidis	497928569	-ISE-NSYLRPL--VINYDE-V		EPVDVQKV-EKAK--KE-MFIS
	Stomatobaculum longum	497218087	-LQV-KT--ANF-LN-P-KNGE		-TPREIQK--LR-E--ET-D--S
	Novispirillum itersonii	518473365	RLEA-RS-LQT-NLN-PTGALR		PQNFNTVISK-R--SQ-H---
	Bartonella elizabethae	492187130	-QE--R--LQSL-IS-SKSAEL		TSAR-NRI-EK-AH-EQQE---
	Bartonella tribocorum	163868292	-QE--RT-LQSL-IS-SKSAEL		TSAR-NKI-EK-AH-EQQE---

Figure S30

Partial sequence alignment of the protein ribonuclease R, showing a 4-5 aa insert (boxed) that is found in all members belonging to the order *Chlorobiales.*

			640		720
**Order *Chlorobiales***	*Chlorobaculum parvum*	193213033	HFTSPIRRYPDLVIHRILFEY	ETTR	KKRRKVTPERITEISATITEVCQITNEREKIATEAERE
	Chlorobaculum tepidum	21673344	------------IV-------	----	-----------SQ-T----------------------
	Chlorobium phaeovibrioides	145219389	------------IV--M----	-AF-	-----I--A--A-LTQK-ET--R-S-------V-----
	Chlorobium ferrooxidans	493409164	------------IV--L----	-GL-	-----I-SA-LA-LTDK-QT----S-----S-V-----
	Chlorobium phaeoclathratiforme	194336956	------------IV--M----	-NL-	-----ISAT-LA-LTDK-QT----S-----S-V-----
	Chlorobium luteolum	78186405	------------IV--L----	-AF-	-----MPEA--A-LKQK-DT--R-S-----V-V-----
	Chlorobium chlorochromatii	78188726	------------L---M----	-RM-	-----MSET-LG-LET-MQR----S-----S-A-----
	Prosthecochloris aestuarii	194333503	-------------V--L----	-SL-	-S-K-EPLR-LK--ESK-V-I-K-A-----S-V-----
	Chlorobium limicola	189346025	------------IV--L----	-NL-	NRKK-I-DK-LA-L-EK-RVTSR-S-----S-V-----
	Prosthecochloris ethylica	NUK48115	------------IV--L----	-SF-	-Q-K--PAK-LK--GGV-D---T-A-----S-V-----
	Chlorobium phaeobacteroides	189499728	------------MV--L----	-AS-	-R-K-LSAK-LA---TK-EQI-KNS-----S-M-----
	Chloroherpeton thalassium	193216324	------------IV--L-Y--	-NR-	-S-K--PQK--A-LRSK-PL--EQASAQ-QN-A----D
	Chlorobaculum limnaeum	2501894184	------------IV--L----	-GA-	---K-IS----SA-TGMLGS----S-------------
	Prosthecochloris marina	110024153	-------------V--L----	-AY-	-S---IPSK-L-D---K-EQ---VA-----S-M-----
	Chlorobaculum thiosulfatiphilum	TNJ39330	------------IV--L----	-GA-	---K-IS----SA-TGMLGS----S-------------
	Cand. Thermochlorobacter aerophilum	RFM24073	-------------T--L-Y--	--L-	QSQ--LSKP-LRKLN-L-PAI-EHAS-Q-RNA-----D
	Candidatus Chlorobium masyuteum	166807966	------------IV--L----	-GL-	-----I-SA-LA-LTDK-QT----S-----S-V-----
	Chlorobium sp. N1	131358104	------------LV--M----	-AF-	----RMPEA--N-LKGKLEA--R-S-----V-V-----
	Chlorobium sp. KB01	076791152	------------IV--L----	-GL-	-----ITSA-LA-LTDK-QT----S-----S-V-----
	Chlorobaculum sp. 24CR	RXK82182	---------------------	----	--------------------------------------
	*Prosthecochloris sp. HL-130-GSB_*	085659969	------------IV--L----	-AS-	-Q-K--A-K-LKS-GGL-D---R-S-----S-V-----
	Prosthecochloris sp. GSB1	094082062	---------S---V--L----	-HL-	-----MS-G-LAGI-SR-D-----A-----S-V-----
	Prosthecochloris sp. ZM_2	114608494	------------IV--L----	-SF-	-Q-K--PAK-LK--GGV-D-----I-A---S-V-----
**Larger *Ignavibacteriae* Clade**	*Chlorobi bacterium NICIL-2*	KXB97643	-------------V--L-K-		SLG-P--Q--AHLRDRLEPIAEH-S----RH-V----
	Chlorobi bacterium OLB7	KXK55686	------------IV--M-A--		EAGMPVRQRN-YA--LGPIADQCS---RA-V----D
	Chlorobi bacterium OLB5	KXK52504	------------IV------V		LNGLNAK-AQHYHKQLPDI-KQST-T-IN--Q----
	Ignavibacterium album	385809169	---------S--LV--L--K-		IEN-QLPGYTLEELE-I-EHISAC-RT-M----Y
**Other Bacteria**	*Dorea longicatena*	548195207	------------Q----IKDN		L-GRMNESK-EHYQSILP--AKRSS-M-RR-D-----
	Bacteroides dorei	547774176	--------F--MMV--L-TR-		LAGGR-VQETKYE-L-DHSS-M-Q--AN---A
	Megamonas hypermegale	479207615	------------IV--L-K-Q		LNG-M-A--QEKLKSRLP--ATH-SKQ-R--------
	Tepidanaerobacter syntrophicus	059033581	---A--------E-----R-L		QEN-LDLK-QEKL-K-VEK-AK-SS---R--D-----
	Megamonas funiformis	225091553	------------IV--L-K-Q		LNG-M-A--QEKLKSRLP--ATH-SKQ-R--------
	Moorella mulderi	KYH31529	-------------V----R-T		WSPGGL--Q-MA-LE-FVALAAAQAS----V-E-----
	Biomaibacter acetigenes	122015104	------------M----IR-H		LEGRLDAK-QEKL-KIVAR-AK-SS---R--D-----
	Aneurinibacillus aneurinilyticus	ERI11065	------------IV--MIR-S		L-KGGF--Q-ME-WY-KMPQ-AEQSS---RN-VD----
	Megamonas hypermegale	CBL07268	------------IV--L-K-Q		LNG-M-A--QEKLKSRLP--ATH-SKQ-R--------
	Alicyclobacillus mali	206885292	------------A----IR-V		LT-GSLS-K-EA-LREFVA-ASRQSS---R--Q-----
	Thermovenabulum gondwanense	068747371	------------I-----R-Q		LEGGI-EK-QEQL-KILGK-AKLSS---R--DD----
	Moorella stamsii	PRR71716	-------------V----R-T		WSPGGL--Q-MA-LD-FVALAAAQAS----V-E-----
	Alicyclobacillus mali	206881148	------------A----IR-V		LT-GSLS---EAQLREFVADASRQSS---R--Q-----
	Peptococcaceae bacterium	MBU7006244	-----------------IK-N		LAKGRL-EK-K-KLASFVADAAIQSS---R--------
	Paludifilum halophilum	OYD08178	------------I----IW-V	W	ENGGTLSR--KN-LDEWLP-A-KHAS---RL-ID----
	Aneurinibacillus migulanus	KIV50047	------------IV--MIR-S		L-KGGF--Q--E-WY-KMPQ-AEQSS---RN-VD----
	Laceyella tengchongensis	154987632	------------L---VIR-I		VEKGAL-V---D-LN-YL-DAA-QSSI--RV-VD----
	Ruminiclostridium cellobioparum	200859494	------------I----MKQ-		LKGEM-EQ--GQLEGMLP-IAKQCS---RA-D-----
	Brevibacillus fulvus	204519821	------------IV--LIR-W		VE-GEM-G--MSYW-EKLP-IADHSSQ--RL-V-----
	Candidatus Kryptonium thompsonii	CUS84555	-------------V--L-Y--		TEKG-DDK-LR--AQKLPQI-KHSS-M-IR-M-V---
	Effusibacillus pohliae	018132105	-----------------IR-A	I	EHGGTLP----DQLR-KLP-IA-HSS---RL-VD----
	Candidatus Thermokryptus mobilis	HGD82170	-------------V--L-Y--		TEGR-DDK-LN--AEKLPHI-KHSS-M-IK-------
	Megamonas funiformis	495813334	------------IV--L-K-Q		LNG-M-A--QEKLKSRLP--ATH-SKQ-RV-------
	Megamonas rupellensis	517829942	------------IV--L-K-Q		LNG-M-A--QEKLKSRLP--ATH-SKQ-RV-------
	Selenomonas artemidis	496716638	------------MV--L-R-T		FATGHISA--REKLR-ILP-TAEHSSA--R--V-----
	Mitsuokella multacida	492427663	------------IV--L-R-T		FATGSIPA--QAKLRTMLP-IADHAS---R--I-----
	Ruminococcus lactaris	491807899	------------Q----IK-N		L-GRL-G--MEHYRKIL---ASQCSQ--RT-E-S---
	Dorea formicigenerans	491479248	------------Q----IKDN		L-GRMNG--MEHYRKILE--TKHAS-T-RR-D-----
	Clostridium termitidis	490767815	------------I----MKQ-		LKGEM-EQ--GQLEGMLP-IAKQCS---RA-D-----
	Moorella thermoacetica	83589142	-------------V----R-T		WSPAGFSA--MAQL--FVALAAVQAS----L-E-----
	Eubacterium ramulus	545613973	------------Q----IKDN		L-GRMNEK--EHYWKILP--A-Q-SQL-RR-D-----
	Dorea longicatena	493473057	------------Q----IKDN		L-GRMNESK-EHYQSILP--AKRSS-M-RR-D-----
	Alicyclobacillus acidocaldarius	258510746	------------A----IR-V		LT-GSLS-Q-EAHLREFVA-ASRQSS---R--Q-----

Figure S31

Partial sequence alignment of the protein ribonuclease R, showing a 4 aa insert (boxed) that is found in all members belonging to the order *Chlorobiales.*

Group	Species	Accession	128		161
Order *Chlorobiales*	*Chlorobium luteolum*	78186789	KDVDGFHPENLGRLVMGH	LD	KCFVSCTPYGILEL
	Chlorobium phaeovibrioides	145219770	------------------	--	--------------
	Chlorobium limicola	189346631	------------------	--	--------------
	Chlorobium chlorochromatii	78188750	------------------	--	--------------
	Chlorobium phaeoclathratiforme	194336300	------------------	--	--------------
	Chlorobium phaeobacteroides	119356824	------------------	--	--------------
	Chlorobium ferrooxidans	493409873	-----------------E	--	--------------
	Chloroherpeton thalassium	193214168	----------V----L--	--	--------F--I-I
	Prosthecochloris aestuarii	194333994	----------V-QM-L-N	--	---I----F-----
	Prosthecochloris vibrioformis	139626438	----------V-QM-L-N	--	--YI----------
	Prosthecochloris aestuarii	012505742	----------V-QM-L-N	--	---I----F-----
	Prosthecochloris marina	110022066	----------V-QM-L-N	-N	---L----------
	Candidatus Chlorobium masyuteu	166808243	-----------------E	--	--------------
	Cand. Thermochlorobacter aerophilum	RFM24899	----------V----L-R	-E	E---------V---
	Cand. Thermochlorobacteriaceae GBChlB	KER10279	----------V---MI-R	--	E--I----F-VV-
	Chlorobium sp. 445	PI047889	----------V----I-R	-N	D-------L-V---
	Prosthecochloris sp. CIB 2401	068867189	----------V-QM-L-N	--	--YI----------
	Prosthecochloris sp. GSB1	094083698	----------V-QM-L-N	--	--YI----------
	Prosthecochloris sp. ZM	114617229	----------V-QM-L-N	--	---I----F-----
	Chlorobium sp. N1	131353427	------------K----E	--	--------------
Larger *Ignavibacteriae* Clade	*Chlorobi bacterium OLB5*	KXK44039	--------V-A---MI-E		---TP---A--H--
	Chlorobi bacterium OLB6	KXK33887	--------M-A---LI-Q		-G-AP---A--M-M
	Chlorobi bacterium NICIL-2	KXB98178	--------V-V----V-L		PALLP---A--V--
	Melioribacter roseus P3M-2	397690900	--------Q-V-N--I-K		DT-YP------V-I
	Ignavibacterium album JCM 16511	385810387	--------MSV-N--I-K		ET------A--Q--
Other Bacteria	*Thermoflexus hugenholtzii*	088572012	--------A-----GLKG	K-	P--IP---L---AM
	Roseburia inulinivorans	547180385	--------Q-V-K--I-E		EG------A-VIQ-
	Parabacteroides goldsteinii	494928053	--------I-V--MSI-L		P----A--A-----
	Bacteroides pyogenes	567224504	--------I-V--MSI-L		P-Y--A--N-----
	Barnesiella viscericola	570742677	--------V-V--MSI-L		P----A--A--M--
	Caldimicrobium thiodismutans	068516299	--------V-V-K--I-E		P--IP-----V-M-
	Thermodesulfobacterium geofontis	HHH86329	--------I-V-KM---E		P--LP-------V-
	Dissulfurirhabdus thermomarina	163298669	--------V-V-KM-I-E		Q--LP---H----M
	Cyprideis torosa	CAD7233470	--------V-I--M-L-E		Q--LP---H-V---
	Thermodesulfobacterium hveragerdense	028841618	--------F---------		PT-IP-------V-
	Massilibacterium senegalense	062198499	--------M-V-KMLL-E		E--I----F----M
	Thermodesulfobacterium commune	038060483	--------F---------		PT-IP-------F-
	Sporosarcina psychrophila	HJF33052	----------V-KMII-Q		-S-L-------IK-
	Vagococcus vulneris	125984437	--------M-M-K-L--E		PDKIA------I--
	Lachnobacterium bovis	027421766	----------V-A--I-Q		-G------F--IQ-
	Fictibacillus gelatini	026676258	--------V-V-KMML-Q		E--I----------
	Candidatus Electrothrix aarhusiensis	RWX44970	--------V-V-KM-V-E		Q--LP---H-V---
	Desulfobulbus propionicus	043770998	--------V-V-KMML-E		R--LP---H-----
	Desulfocapsa sulfexigens	HIP82974	--------V-V-KM-L-E		---LP---H----M
	Dissulfuribacter thermophilus	067616757	--------V-V--M-I-E		R--LP---H----M
	Bacteroides ovatus	490428650	--------I-V--MSI-L		P-Y--A--N-----
	Parabacteroides distasonis	150006885	--------I-V--MSI-L		P----A--A-----
	Pontibacter roseus	517289359	--------A-V--M-A-L		SS-LPA------Q-
	Roseburia hominis	347531843	----------V-K--I-E		PG------A--IQ-
	Ruminococcus lactaris	565839991	---------SV---WI-E		-G-L----A-VIQ-
	Desulfocapsa sulfexigens	451948750	--------V-V-KM-L-E		Q--LP---H-----
	Desulfurivibrio alkaliphilus	297568807	--------V-V-KM-L-E		Q--LP---H-----
	Sphaerochaeta pleomorpha	374314810	--------I-V-K-LI-L		P-------K-V--M
	Sporosarcina newyorkensis	497174567	--------M-V-KMLI-Q		QT-LP------MQ-

Figure S32

Partial sequence alignment of the protein bifunctional 5,10-methylene-tetrahydrofolate dehydrogenase, showing a 2 aa insert (boxed) that is found in all members belonging to the order *Chlorobiales.* The CSI is not present in any other bacterial species except for one other bacterium as indicated above, in the top 500-BLASTp hits.

Group	Organism	ID	5		46
**Order *Chlorobiales***	*Chlorobium luteolum*	78187149	IDHIAIAVSNLDEAVRTYMNV	LG	CGADAIRIEEVPSEQVRVA
	Chlorobium phaeoclathratiforme	194336268	--------Q--ES-LE-FR--	--	-ARE----------N----
	Chlorobium limicola	189346400	--------Q--ES-LE-F---	--	-APGD-T-------K----
	Chlorobium phaeovibrioides	145219844	--------T---D-LK-FTSL	--	SRENEV-V----T-G----
	Chlorobium ferrooxidans	493411298	--------Q--EVSLE-FL--	--	-DPKSVT-------N----
	Chlorobaculum parvum	193212493	--------QD-NS--D-FVA-	--	-ERSSVT-H---A-K----
	Chlorobium phaeobacteroides	119356984	--------E--ER-IE-FKS-	--	-ASET-I----A--N----
	Chloroherpeton thalassium	193214308	---V----NSI-ATLA-FKQL	VD	E-VAS-SY----AQK-K--
	Prosthecochloris aestuarii	194333709	--------ND--S-IQQFSAL	T-	IDPESVH--Q----K----
	Chlorobium chlorochromatii	78189093	--------Q---N-LD-FCTI	--	VDRQSVH----A--K---G
	Chlorobaculum limnaeum	2501895295	--------E---S-ID-WI-I	--	-DRS-VT-H-----K--A-
	Prosthecochloris marina	110023485	--------HD-ENSLEQFKIL	TD	ATDAQ---------K----
	Chloroherpeton thalassium	012499144	---V----NSI-ATLA-FKQL	VD	E-VAS-SY----AQK-K--
	Chlorobaculum thiosulfatiphilum	139457284	--------EH--S-ID-WT-L	--	-DRS-VT-H-----K--A-
	Candidatus Chlorobium masyuteum	166808062	--------Q--EVSLE-FL--	--	-DPKSVT-------N----
	Cand. Thermochlorobacter aerophilum	RFM24960	---V----RSI-A-LA-FSQL	--	TEVLR-T---L-QHH----
	Chlorobium sp. 445	PI047471	--------HS--A-LE-FSKL	-S	GSTMH-T---L-AHH--I-
	Chlorobaculum sp. 24CR	129050599	--------E---N-ID-WTKL	--	-SRS-VT-H-----K--A-
	Prosthecochloris sp. GSB1	094082721	---------D-ES-LERF-AL	T-	IAPGN---------K----
	Prosthecochloris sp. ZM	114618132	--------ND--S-IQQFSAL	T-	IDPESVH-------K----
	Prosthecochloris sp. HL-130-GSB	085660616	--------RD--S-IER-IAL	T-	ISRESVHV----A-H----
Ignavibacteriae	*Ignavibacterium album*	385810015	-E--G---K--H--IKF-EE-		F-LKCYAV---ADQK--T-
	Melioribacter roseus	397689645	-E--G---K--EQ-IKY-EE-		L-LKCYS---IRTQKT-T-
**Other Bacteria**	*Calditerrivibrio nitroreducens*	313673009	----G---KS----LIF-KSI		-VSPYHF---E-QK-K--
	Deferribacter desulfuricans	291280307	----G---K-I---LSL-KTL		-ITPYHF---E-QK-KT-
	Denitrovibrio acetiphilus	291288330	----G---K--E-TAF-KTM		-IEPYHF---I-QR-K--
	Mucispirillum schaedleri	555549200	-N--GV--KSIEA-LPF-KAM		---INH------QK-KT-
	Flexistipes sinusarabici	336322433	----G---KS-E----F-ETL		-VSVESY---T-QK-K--
	Geobacillus thermoleovorans	375009421	V---G---RSIEK-LPF-TD-		L-LPFLG----E----K--
	Geobacillus kaustophilus	519662137	V---G---RSIEK-LPF-TD-		L-LPFLG----E----K--
	Anoxybacillus kamchatkensis	518246766	V---G---KS----LPF-TDT		L-LSCVG--T-E-------
	Akkermansia muciniphila	BBP47461	----G---KS--AT-PY-RDA		L-LGEPH-----TQK----
	Nitrososphaeraceae archaeon	MBD0360489	--------KSV----KD-QS-		LNI-QLEF-V--N-K--
	Caldithrix abyssi	HHJ53415	-N------ES----IPY-RD-		L-LEL-AT----DQK----
	Chrysiogenes arsenatis	027389778	----GV--RS-----GF-RAM		--EPYHF-----QK----
	Acinetobacter baumannii	171253172	-E--G---K------QF-TQI		L-LKL-GF-T-E--K---
	Fictibacillus phosphorivorans	153237885	----G---KS----LPF-V--		L-LTLEA------QK-K--
	Ureibacillus manganicus	036186694	----G---N----RITY-TET		L-LKLLK------QN----
	Lysinibacillus halotolerans	122973306	V---G---K----RITY-TET		L-LTLLK------QH----
	Psychrobacillus glaciei	151700256	V---G---KSIE--LPY-IET		L-LKL-H-------K----
	Candidatus Nitrosotalea okcheo	157927039	L--V----N---A--KQ-KE-		L-VGDVEF-T-E--G----
	Ktedonosporobacter rubrisoli	207229296	---V--I-K-IEQ-LSF-RD-		L-ITPSA-K---T----I-
	Candidatus Nitrososphaera gargensis	015019962	V-------K-VE--LKN-QKI		LNV-HLE-----N-K-K--
	Paenisporosarcina indica	075618245	V---G---K----TIPY-TET		L-LTLLK----A--S----
	Kurthia huakuii	029499089	V---G---K-I--RIAY-TD-		L-MNLEKV---E----K--
	Psychrobacillus soli	142606884	V---G---K-IEDSLPY-TET		L-LKL-H-------K----
	Lysinibacillus timonensis	106779771	V---G---R--N-RITF-TET		L-LKLLK-----TQH----
	Kurthia zopfii	109350013	V---G---KD--ARITY-T--		L-MKLLNV---Q-------
	Paenactinomyces guangxiensis	181752266	-S--G---Q---SS--W-QE-		L-LPFEG--A-E-------
	Rummeliibacillus stabekisii	066786744	----G---K----RTTY-T--		L-MKLLN----E--G----
	Planococcus chinensis	MBM7531103	V---G---R--E-VIPY-TET		L-CPLLK----A-QK----
	Ureibacillus sinduriensis	036202110	V---G---KD--KQ-TY-TET		I-LKLLK------Q-----
	Lysinibacillus fusiformis	205444228	V---G---R----RITY-TEI		L-LKLMKV---E-Q-----
	Nitrosopumilales archaeon	RPJ30166	V--V----NSIS--LKN-EKI		LKI-K-N---I-T-R-K--
	Rummeliibacillus stabekisii	147193591	----G---K----RTTY-T--		L-MKLLN----E--G----
	Ureibacillus massiliensis	036173336	V---G-S-R--E-QITY-TE-		L-LKLLK------QN----
	Planomicrobium mcmeekinii	MBP1899710	V---G---RK--DVIPY-TET		L-CPLLK----E--R----
	Anoxybacillus flavithermus	491027944	V---G---KS----LPF-TDT		L-LSCVGM-T-E-------
	Bacillus methanolicus	490570485	V---G---KS--KTLSF-TE-		LRLELLG----E--K-K--
	Geobacillus thermodenitrificans	138895934	V---G---RSIEK-LPF-TD-		LRLPFLG----E----K--
	Lysinibacillus sphaericus	499131666	V---G---R----RITY-TEI		L-LKLLKV---E-Q-----
	Bacillus massiliosenegalensis	517984218	V---G---KSME--LPF-TD-		L-LEVQA--T-E--K-K--
	Geobacter bemidjiensis	197116956	-N-VGV--AS-E--LPF-RDS		L-MTFSGT-----QL----
	Geopsychrobacter electrodiphilus	522166183	-N--G---K--ET--PF-RDT		L-MTFEGT---TEQK----
	Desulfurispora thermophila	516757206	----G---K--QQ-IEF-ETT		L-LKVAG-----EQK----
	Halanaerobium saccharolyticum	491632004	-E--G---Q--E-SLKF-QDI		L-LEFLKQ-T-E-QG-KI-
	Coraliomargarita akajimensis	294054336	----G---KS---SIEY-EKA		L-LHCHGK---E-QK-KT-
	Butyricimonas synergistica	517147053	-E--G---AS----IPY-E--		L-LKCYA----KDQK-KT-
	Porphyromonas gingivalis	34541263	-E-LG---KSIE--LPY-E--		L-LKCYS-----DQK--T-
	Archaeoglobus sulfaticallidus	488600058	----G---K------EL-KKL		-FEVKG----EEQK-K--
	Ferroglobus placidus	288932190	----GV--K--E--IEL-KKL		-FEVKG----KDQK----

Figure S33

Partial sequence alignment of the protein lactoylglutathione lyase, showing a 2 aa insert (boxed) that is found in all members belonging to the order *Chlorobiales.*

			55		106
Order *Chlorobiales*	*Chlorobium luteolum*	78187419	LVTVALRRFNREQLEDDLFGPLSQL	E	GITLMPNTSGASTAAEAVKAAHISRE
	Chlorobium phaeovibrioides	145220164	-----------------------E-	P	-----------------I---N----
	Chlorobium ferrooxidans	493409422	----------------------KE-	S	------------------R----A--
	Chlorobium phaeoclathratiforme	194337038	-I-----------A---------AI	-	-V---------------IR----A--
	Chlorobaculum parvum	193212337	-------------A---------EI	-	-L---------A--K-----------
	Chlorobium chlorochromatii	78189233	-------------A---------A-	-	AV---------------IR----A-Q
	Chlorobaculum tepidum	21673528	-------------A---------EI	-	-L---------A--K--I-----A--
	Chlorobium phaeobacteroides	119356564	-------------IA--------A-	Q	--------------R--IH----A--
	Chlorobium limicola	189346198	-------------PG----T--TSI	P	------------------Q--R-A--
	Chlorobaculum limnaeum	2501893292	-------------A---------AI	K	-L---------A--K-----------
	Chloroherpeton thalassium	193215746	M----I--MDLKSDSAENIMKFLDD	K	KYF-L---A-CY--K---LT-ELA--
	Prosthecochloris marina	110022181	-------------V-H---AA--TI	-	-L----------N-Q---R-------
	Chlorobaculum thiosulfatiphilum	139456668	-------------A---------EI	-	-L---------A--S-----------
	Prosthecochloris marina	110022181	-------------V-H---AA--TI	-	-L----------N-Q---R-------
	Candidatus Chlorobium masyuteum	166807938	-------------------------	P	------------------R----A--
	Chlorobium sp. KB01	076790525	----------------------KE-	S	------------------R----A--
	Chlorobium sp. N1	131354040	-----------------------E-	-	-L------------------------
	Chlorobaculum sp. 24CR	129050323	-------------A---------EI	-	-L---------A--K-----------
Other Bacteria	*Plesiomonas shigelloides*	499149739	---M--K-VDCGGGQ--ILT--RAM	P	HLRWL------RD-K--IL---LA--
	Aeromonas veronii	330831321	---M-IK-LEPGKVH--ILS--L--		-VK-L------K------F---LA--
	Vibrio fluvialis	521102759	---M--K-VDLAERD--ILA--IEA		--H-L------K--RD--F---LA--
	Grimontia hollisae	491643491	---M--K-VDIQNR---ILA--IAS		--N-L------K--K---F--RLA--
	Photobacterium leiognathi	565848455	---M--K-V-IAERD--ILA--I-A		-VN-L------KN-K--IF--QLA--
	Photobacterium angustum	491514775	---M--K-V-IAERD--ILA--I-A		-VN-L------KN-K--IF--QLA--
	Aeromonas media	491473250	---M-IK-LEPGKRH--ILS--LK-		-VK-L------K------F---LA--
	Vibrio ordalii	498005262	MA-M--K-VDLN--N--IL---IE-		-VH-L------KN-KD-IF---LA--
	Enterovibrio norvegicus	515572621	---M--K-VDVQNR---ILA--IAA		NLN-L------K--K--IF--RLA--
	Salinivibrio costicola	543216350	---M----VDVHATQ--ILT--RE-		-VR-L------KN-Q---F--RLA--
	Vibrio mimicus	446854660	---M--K-IDLA-RD--ILA--LDM		KMN-L------KN--D--Y--QLA--
	Photobacterium damselae	491444567	---M--K-VELGKKY--ILS--K--		-VA-L------K--E--IF--RLA--
	Aeromonas molluscorum	492609492	---M-IK-LEPGKRH--ILS--LA-		-VK-L------K------F---LA--
	Vibrio metschnikovii	490528628	---M--K-VDLQHR---ILA--IAA		-VQ-L------K--R--IY---LA--
	Glaciecola punicea	492851889	---L-IK-IDIR-ST-N-MA---S-		-VK-L------RN-Q--IF--QLA--
	Enterococcus gallinarum	537098851	---M-IK-LEPGKAH--ILS--L--		-VK-L------K------F---LA--
	Cellulophaga algicola	319954194	-I----K-VDL-CES---LSY--HD		N-S-L-----VR--K---F--QL---
	Cytophaga aurantiaca	517154023	--------IDI-GQ---ILKW-KKG		NFN-L------RN-K---F--TLA--
	Cytophaga hutchinsonii	110636668	--------IDIDGQ---ILKW-KKG		NFN-L------RN-K---F--TLA--
	Winogradskyella psychrotolerans	527071521	-I----K-VDVQNN---ILRH-KHA		H-N-L-----VR--K---F--EL---
	Butyricimonas synergistica	517147441	------K-VDL-SK---ILAH-KAP		H---L-----VR--K---FV-QLA--
	Desulfocapsa sulfexigens	451947508	M-------V-LDNP---IMKA---R		DY-FL------RN-N--IRL--LA-A
	Aeromonas veronii	167573688	---M-IK-LEPGKAH--ILS--L--		-VK-L------K------F---LA--
	Aeromonas hydrophila	206819215	---M-IK-LEPGKAH--ILS--L--		-VK-L------K------F---LA--
	Shigella flexneri	EFZ8856520	---L-MK-V-LR-HN--ILA--IAE		-V--L------K--E--IF---LA--
	Escherichia albertii	149451965	---L-MK-V-LR-HN--ILA--IAE		-V--L------K--E--IF---LA--
	Kistimonas asteriae	211826209	-----MK-VDLQ-Q---IL---Q-A	C	DVR-L------R--E---F--KLA--
	Kiritimatiella glycovorans	052882252	-I----------KT----YA--AS-	-	--A--------RD-D--CR--RLG--
	Pontiella desulfatans	VG015799	-----------D-A----Y------	-	NVR--------MN-K---R--MLG--

Figure S34

Partial sequence alignment of the protein thiazole synthase, showing a 1 aa insert (boxed) that is found in all members belonging to the order *Chlorobiales.*

Group	Species	Accession	599		644
Order *Chlorobiales*	*Chloroherpeton thalassium*	193213813	GRHPVLEKIMPIDRKYVPNDCRLDVE		TRVQIITGPNMSGKSSFLRQ
	Prosthecochloris vibrioformis	139626255	-------R-LGV-EA--A---SF-EQ		E-MR-V-----A--------
	Chlorobium phaeoclathratiforme	194336140	-------RM-SAEDP------HF-EK		QQML-------A--------
	Chlorobium ferrooxidans	493410219	-------R--SD-EP--Q---LF-EK		QKML-------A--------
	Chlorobium chlorochromatii	78189282	-------RLLGAEES-I----HF-DK		QTML-------A----Y---
	Chlorobium phaeovibrioides	145220133	-------R--KP-EPF-K---HF-GQ		Q-MLM------A----Y---
	Chlorobaculum parvum	193213235	-------R-LGA-EP--A----VGSD		QQLL-------A----Y---
	Chlorobium phaeobacteroides	189499799	-------R-LHV-EP------FF-KK		Q-MLMV-----A----Y---
	Chlorobaculum tepidum	21674323	-------R-LGA-EP--A---QVGS-		QQLL-------A----Y---
	Prosthecochloris aestuarii	194334404	-------R-VDV-EP--S---LF-ER		Q-ML-V-----A----Y---
	Chlorobium limicola	189347201	-------R-L-A-EP-IA--ALF-DR		QKMLM------A----Y---
	Prosthecochloris ethylica	175186793	-------R-LGV-EP--A---TF-D-		Q-MM-V-----A----Y---
	Chlorobaculum limnaeum	2501895063	-------R-LGA-EP--S----VGAS		QQLL-------A----Y---
	Chlorobium luteolum	78186335	-Y--W-L-SHR--EVL-L-LE-GLN		EQ-L--S---AG---VAMKT
	Prosthecochloris sp. CIB 2401	068866586	-------R-LGV-EA--A---SF-EQ		E-MR-V-----A----Y---
	Prosthecochloris sp. ZM_2	114607669	-------R-LGV-EP--A---TF-D-		Q-MM-V-----A----Y---
	Prosthecochloris marina	167392938	-------R-LGV-EP--A---FF-RK		Q-ML-V-----A--------
	Prosthecochloris sp. HL-130-GSB	085659292	-------R-LGV-EP--A---VF-AQ		H-MM-V-----A----Y---
	Prosthecochloris sp. GSB1	094082121	-------R-LDVGEP------E-NDR		Q-MLVV-----A--------
	Chlorobium sp. KB01	076791947	-------R--SD-EP--Q---LF-E-		QKML-------A--------
	Candidatus Chlorobium masyuteum	166806887	-------R--SD-EP--Q---LF-EK		QKML-------A--------
	Chlorobium sp. N1	131353967	-------RLLSPEDPF-S---LF-EQ		Q-MLM------A----Y---
	Prosthecochloris sp. ZM	114618085	-------R-VDV-EP--S---LF-ER		Q-ML-V-----A----Y---
	Chlorobaculum sp. 24CR	129051820	-------R-LGA-EP-IS----VGAS		QQLL-------A----Y---
Larger *Ignavibacteriae* Clade	*Chlorobi bacterium OLB7*	KXK55477	-----V-TLL-PGER-----TA--TA	A	D-IA-----------Y---
	Chlorobi bacterium OLB6	KXK35082	A----I-RLL-AGTR----NIV--T-	T	HQLMVL----------YL--
	Melioribacter roseus	397690923	-----V-Q-L-PGE-FT--S---SSS	E	DQII-L-----A---VY---
	Ignavibacterium album	385809871	-----V-R-LSPGE-FT----K-NNS	D	QQIILL-----A---VY---
Other Bacteria	*Chthonomonas calidirosea*	512552186	-----I-R- GLGGD-I----H--E-		N-LL-----------Y---
	Rubrobacter xylanophilus	108803143	-----V-HA TETPF----A-I-GG		S-L--------A---VY---
	Taylorella asinigenitalis	479191464	-----V-RTI E-FTA---F-NS-		N-M--------G---TYM--
	Desulfurispora thermophila	516757174	-----V--VLD -GSF----LT--KQ		--LILL-----A---T-M--
	Thermophagus xiamenensis	498212269	-----I-TQL-AGEE-----VY--S-	T	QQ-M-------A---AL---
	Prevotella micans	494011157	-----I-TQL--GEQ-----VL--S-	H	QQIM-------A---AL---
	Anaerophaga thermohalophila	498107809	-----I--QL-VGEE-----VF--ND	T	QQIM-------A---AL---
	Gracilimonas tropica	521070739	-----V--SL-MGEPFI---IK--N-	N	DQIL-------A---II---
	Gillisia marina	497914865	-----I--QL-HGEQ--T--VF--R-	T	QQ-IM----------AI---
	Cytophaga hutchinsonii	110636573	-----I--QL-LGES-I---IY--D-	T	QQ-M-------A---AL---
	Nonlabens dokdonensis	443243179	-----I--ML-A-QP-I---VQ--RD	S	QQIIM----------AI---
	Porphyromonas catoniae	491610388	-----I--QL-FGES-----VH--QK	E	-QIMV----------AL---
	Cytophaga aurantiaca	517154534	-----I--QL-LGES-----IY--D-	N	QQ-M-------A---AL---
	Bacteroides ovatus	490426514	-----I--QL--GE--IA--VM--SD	T	QQII-------A---AL---
	Bacteroides vulgatus	511016684	-----I--QL-VGE--IA--VF--S-	T	QQII-------A---AL---
	Eudoraea adriatica	518500928	-----I--QL-LGEA-I---LV-NR-	S	QQIIM----------AI---
	Candidatus Amoebophilus	189502159	-----I-QQLSV-VS-----IY--N-	T	QQ-IV------A---AL---
	Prevotella marshii	494007441	-----I-TQL-LGE------IF--S-	R	QQIM-------A---AL---
	Prevotella nanceiensis	517174161	-----I-MQL--GER-----IL--S-	K	QQIMM------A---AL---
	Bacteroides xylanisolvens	495298839	-----I--QL--GE--IA--VM--SN	T	QQII-------A---AL---
	Kordia algicida	494155465	-----I--QL--GES-IA--VY--RN	T	QQIIM----------AI---
	Halanaerobium praevalens	385799933	-----V--MFT EQF-----Y--QT	E	Q-FV-----------TYM--
	Butyrivibrio fibrisolvens	479189702	-----V-QMINA- QFIS---Q--LD	S	RTIA-------A---TYM--
	Nematostella vectensis	156325874	-----I--QL--GEE-IA--VV-NRN	Q	QQIIM----------AI---
	Caldithrix abyssi	493983722	-----V--TL-PGEDFIA--AY--PD	S	EQIW-------A---T----

Figure S35

Partial sequence alignment of the protein DNA mismatch repair protein (MutS), showing a 1 aa deletion (boxed) that is found in all members belonging to the order *Chlorobiales.* The CSI is not present in any other bacterial species except for the four other bacteria as indicated above, in the top 500-BLASTp hits.

Group	Species	Accession	400		447
**Family *Chlorobiaceae***	*Chlorobaculum parvum*	193212508	NRDPLKTKQLEKGIRQLTDEGVAQLFVQY		GTRKIVGTVGELQFDVIQF
	Chlorobaculum tepidum	010932499	-----------------------------		-------------------
	Prosthecochloris marina	110023507	----F-S------L----E-------L--		-N---L-----------R-
	Chlorobium ferrooxidans	006365821	----M-A---D-------E-------I-H		-A---I-----------K-
	Pelodictyon luteolum	KZK74796	----M-Q---D-------E-L-----I-F		-N---I-----------K-
	Chlorobium phaeoclathratiforme	194336646	----M-A---D--V----E-------I-H		-A---I-----------K-
	Chlorobium phaeovibrioides	145219836	----M-Q---D-------E--L----I-F		-N---I-----------K-
	Prosthecochloris aestuarii	194333731	----F-S---D--V----E-------M-H		-N---I-----------R-
	Chlorobaculum thiosulfatiphilu	139457824	-----------------------------		-------------------
	Chlorobaculum tepidum	21673647	-----------------------------		-------------------
	Chlorobium limicola	189346321	----------D-------E----------		-S---------------K-
	Chlorobium chlorochromatii	78188914	------A---D-------E-------I--		-S---I-----------K-
	Chlorobium phaeobacteroides	189500131	----F-A-----------E-------M--		-N---------------K-
	Chlorobaculum limnaeum	2501894968	--------------H--------------		-S-----------------
	Candidatus Chlorobium masyuteum	166808734	----M-A---D-------E-------I-H		-A---I-----------K-
	Chlorobaculum sp. 24CR	129050574	-----------------------------		-------------------
	Chlorobium sp. KB01	076790347	----M-A---D-------E-------I-H		-A---I-----------K-
	Prosthecochloris sp. HL-130-GSB	085659723	----F-S------V----E--L----I--		-N---I-----------R-
	Chlorobium sp. N1	131353584	-----M-Q-D--------E-L-------F		-N-R-I-----------K-
	Prosthecochloris sp. GSB1	094082708	----F-A------L----E-------L--		-N---I-----------R-
**Larger *Ignavibacteriae* Clade**	*Chlorobi bacterium OLB7*	KXK57314	-A-R-S-Q--LE--RE--AI-VMYSP	N	S-DP-LAA--Q-----VK-
**Other Bacteria**	*Pedobacter arcticus*	515828150	-N--F---------H--S--------I-N	P	-N------------E----
	Proteiniphilum acetatigenes	518370219	-A--M-S---Q---E--M--------TNQ	F	NG---I----Q---E---Y
	Niabella aurantiaca	517456776	----M--------LL-----------T-F	G	--K----C------E---Y
	Mucilaginibacter paludis	495787162	------S-------Q---E---------Q	P	-N---I--------E--S-
	Chitinophaga pinensis	256422385	-K--M--------L------------T-H	N	-N---I-C--D---E---Y
	Dyadobacter beijingensis	518785225	-M--M-S-------Q-----------TLD	L	-N--V-------------Y
	Emticicia oligotrophica	408671881	-K--M-S------VQ-----------TIQ	P	-N---I--------E---
	Rudanella lutea	518834208	-L--M-S-------E-----------TLQ	P	-N------------E---Y
	Arcticibacter svalbardensis	510820811	--------------Q---E-------I-Q	P	-N-----A--A---E--N-
	Segetibacter koreensis	517441155	-K-----------LI-----------T-F	G	-NK--I-C------E---Y
	Runella slithyformis	338213186	-L--M-S------VQ-----------TLQ	P	-N--V---------E---Y
	Bacteroides coprophilus	495420018	-A--M-----N---D--M---------NQ	F	NG---I----Q---E---Y
	Fibrisoma limi	496584223	-L--M-S---D---E-----------TLQ	P	-N--V-----D-------Y
	Pedobacter agri	498284896	-M--MR--------E------------MQ	P	-N--VI-A------E--N-
	Spirosoma linguale	284036038	-L--M-S---D---Q-----------NLE	L	-N------------E---Y
	Niastella koreensis	375146319	-K--M--------LL-----------T-F	G	-NK--I-C--D---E---Y
	Paludibacter propionicigenes	313203001	-A--M-----D---N--M--------TNQ	Y	NG---I----Q---E---Y
	Fibrella aestuarina	436836389	-L--M-S---D---Q-----------TLA	L	-N------------E---Y
	Paraprevotella xylaniphila	495900769	-A--M-----N---E--M---------NQ	F	NG---I----Q---E---Y
	Paraprevotella clara	547245326	-A--M-----N---E--M---------NQ	F	NG---I----Q---E---Y
	Prevotella stercorea	495178579	-D--M-A-------N--M---------NQ	F	NG---I----Q---E---Y
	Bacteroides plebeius	494837401	-A--M-----N---D--M---------NQ	F	NG---I----Q---E---Y
	Bacteroides coprophilus	547871944	-A--M-----N---D--M---------NQ	F	NG---I----Q---E---Y
	Alloprevotella rava	496706421	-A--M-----NR--E--M---------NQ	F	NG---I----Q---E---Y
	Barnesiella viscericola	570741391	-D--M-S---N---D--M--------ISQ	F	NG---I----Q---E---Y

Figure S36

Partial sequence alignment of the protein peptide chain release factor 3, showing a 1 aa deletion (boxed) that is specific for members of the family *Chlorobiaceae.*

Group	Species	Accession	260		316
Family *Chlorobiaceae*	*Chlorobium ferrooxidans*	493410285	PEYVHTATAAYRTAIDFCINRRNDP	QFREEY	SELTTKLKEDLATVYNRGFSKGFYFG
	Pelodictyon phaeoclathratiforme	194337501	--------T---SS----TT-----	E--N--	-K---Q---E----------------
	Chlorobium phaeobacteroides	119357950	--------T---R-----TSH--S-	E--T--	NS-SKQ---E--R-------E-----
	Chlorobium limicola	189347571	--------S---N-----TTN--T-	A-GDG-	NA-SQ----K--L-------E-----
	Chlorobium chlorochromatii	78188525	------T-T---Q-LNM-MQQ-HQA	D--NR-	-A--AS--H----------N-----
	Chlorobium phaeovibrioides	145219174	----QAT-----K------EE-SK-	G--KRN	E--IGN---E--L-------N-----
	Chlorobium luteolum	78186182	----Q-V-S---R-L-C--SE-S--	G--AR-	-A--AE---E-R--------D-----
	Chlorobium phaeobacteroides	189501046	------T-KC--K---YTLEHGHEK	---RHF	EA-AKE-ATE-HK-------H---L-
	Chlorobaculum limnaeum	2501893814	----A-T-----K----IAAH-G-G	D-A-T-	GK-ARE-E-E-KK----------F--
	Chlorobaculum parvum	193211992	----A-T-----K----IAEH-D-E	N-D-A-	RT--DG-QD---R-------E--F--
	Chlorobium tepidum	21674702	----A-T-----K----IAAH---K	D-D-A-	RS--DR-QN--VR-------E--F--
	Chlorobaculum thiosulfatiphilum	139457271	----A-T-----K----IAAH-E-E	D-A-A-	RS-ADG-E-K-KK-------E--F--
	Prosthecochloris marina	110022762	------T-TC--K---YTLEHGHEK	D-SQTF	DA--KE-MKE-E--------------
	Prosthecochloris ethylica	175186871	------T-SI--S-F-YILQHQD--	G-ADT-	RRFAE-QA-E-R-----E--D-----
	Prosthecochloris aestuarii	194334724	------T-SC--Q---YIVAH-HET	S-E-D-	QAVADN-TRQ-K-----E--S-----
	Candidatus Chlorobium masyuteu	166807158	-------------------------	------	--------------------------
	Chlorobium sp. N1	131356391	----Q-V-R-------C-MEE-GK-	G--AR-	G---GR--GE-EK-------S-----
Chloroherpetonaceae fam.nov	*Chloroherpeton thalassium*	193214872	EF--ASV-R---A-L-SYAKNPDTY		TFDPAW-- --EAISH-LY-T--A--
Ignavibacteriae	*Melioribacter roseus*	397690227	---I-KVVS---K---LYYENKLND		-IKSE--NK-TE-------A---L-
	Ignavibacterium album	385811731	----AKVVSV--N---LYFEGKLNT		-KKKEFLTE-E--------S-----
Other Bacteria	*Prevotella denticola*	490483825	------VVTC--E--ESVLDGTFTE		-KKDLWDKR-S--F----WD-Y-Q-
	Prevotella histicola	496098877	------VVSC-KE--ESVLDGTFTE		-KKD-WD-R-S--F----WD-Y-Q-
	Capnocytophaga cynodegmi	517091584	----A-VIKT--E---AYYEGTYSK		-KVE-WM-A-S-------WS-Y-L-
	Prevotella copri	493902504	----Y-VVKC-KE--AAVLDGTFTE		-KKDEWD-K----F----WD-Y-Q-
	Capnocytophaga canimorsus	340620824	----A-VIKT--E---AYYEGTYSK		-KVE-WM-A-S-------WS-Y-L-
	Thermophagus xiamenensis	498212208	----K-VVSC--E--NAVV-NTYSQ		-KIEEWEQR--S-F----WD-Y-L-
	Anaerophaga thermohalophila	498104798	----K-VVSC--E--NSVLDDTYSQ		DKINHWE-S--S-F----WD-Y-L-
	Porphyromonas crevioricanis	565855801	----Y-VVK---E---AVV-GTYSE		DKIAVWD-Q----F----WD-Y-L-
	Ornithobacterium rhinotracheale	392389808	----A-V-KC--E---SISEGTFTQ		-KVAEWMKL-E-------WS-Y-L-
	Porphyromonas cansulci	565858531	----Y-VVK---E---AVV-GTYSE		DKIAVWD-Q----F----WD-Y-L-
	Prevotella bivia	490466178	------VVTC-KE--EATL-GTYTE		-KK-E-D-R-SR-F----WD-Y-Q-
	Methanoplanus limicola	490179276	----K-TVSC--K-L-AVYDGNYS-		--AGE---K--KA------Q---K-
	Methanosarcina lacustris	232308722	-G-LEVVSRC--E---A-KEG-YTR		-KVETW-SE--S-------T-----
	Porphyromonas crevioricanis	023940636	----Y-VVK---E---AVV-GTYSE		DKIAVWD-Q----F----WD-Y-L-
	Pelagicoccus mobilis	200356609	----S-V-----RGV-AIQSGTFTE		D-V-Q-ND--KK--H--H-S-Y-L-
	Methanosarcina vacuolata	048123772	-G-LEMVSRC--E---AF-EGNYT-		-KIETWRRE--S-------T-----
	Porphyromonas gingivalis	077094689	----Y-VCRC-KE--EA-C-GTY-E		-AIGRWN-Q----F----WD-Y-L-
	Saccharicrinis carchari	142532834	------VVK--NA-LNAILDGSYG-		KKIEDWNKE----F----WD-Y-L-
	Geosporobacter ferrireducens	069974372	----A-IVG---K---QFCEKGIQ-		K-DQR--I-V-QIF--K-T--YL--
	Capnocytophaga felis	155283725	----A-VIKT--E---AYYEETYSK		-KVG-WM-A-S-------WS-Y-L-
	Coraliomargarita akajimensis	013044600	----KLV-R---S-VNAVFDGTYSR		-FVET-M--IKK-----L-D-Y-L-
	Capnocytophaga cynodegmi	212923789	----A-VIKT--E---AYYEGTYSK		-KVE-W--T-S-------WS-Y-L-
	Methanosphaera stadtmanae	MBE6485441	-D--S-V-NC-SD--KLYE-GLW-Q		SSK--IP-W--E-KS-F----DT----
	Porphyromonas gingivalis	097626982	----Y-VCRC-KE--EA-C-GTY-E		-AIGRWD-Q----F----WD-Y-L-
	Salinimicrobium sediminis	097056891	----A-V-K---E---SWYEGTYSE		-KVADWM-R-S-------WS-Y-L-
	Petrimonas mucosa	HHT30867	----R-V--C-KE-VEAYC-NSFSQ		DKIELWN-K----F----WD-Y-L-
	Methanobrevibacter millerae	MBE6504141	-D-GAMV-SV--E---SYL-NPDEY		-FNPQW--E-ES-F----DT----
	Caldanaerobacter subterraneus	011025895	----ASVVK---D---SYYEE-AFD		S--AM-EM-K-F-----T-YL-
	Pelagicoccus enzymogenes	191615848	----S-V-----RGV-AIQSDTF-Q		P-VAE-ND--KK--H--H-S-Y-L-
	Porphyromonas gingivalis	097552668	----Y-VCRC-KE--EA-S-GTY-E		-AIGRWD-Q----F----WD-Y-L-
	Salinimicrobium catena	093113199	----A-V-R---E---AWYEGTYSE		-KVE-WMGE-S-------WS-Y-L-

Figure S37

Partial sequence alignment of the protein U32 family peptidase, showing a 6 aa insertion (boxed) that specific for members of the family *Chlorobiaceae.*

Group	Species	Accession	329	Insertion (boxed)	382
**Family *Chlorobiaceae***	*Chlorobium phaeovibrioides*	145220314	LPLAYFHVFSCSVRPGTV	LAREV	SCGERLAVAPQVASGRSAILKEIGERKAAEF
	Chlorobium ferrooxidans	493410273	-----L------I----A	---QT	ASR-LI----AEIAR--RV-NDL-R--E-A-
	Chlorobium phaeobacteroides	119356260	--ISSL-----------Q	--EQ-	ATKK-EV-D-DECRQ--SE-IKL-NL-KSR-
	Chlorobium luteolum	78187582	---S-L-----------L	-G-Q-	AG----R-N-KETAR---L-V-L-----R--
	Chlorobaculum parvum	193213382	--V--L-----------R	-S--I	AEKRVSR-VSAE--R-ATR-AQL-QQLERR-
	Chlorobium limicola	189345895	--I--L---T--------	--AQL	AAD--RLPS-VELDR--RL-I-L-K--TE-Y
	Pelodictyon phaeoclathratiforme	194335608	----SL------I----R	--LQI	TNS--KP-ESAEIAR-YSE-A-L-R-HE-R-
	Prosthecochloris aestuarii	194334590	-----L-I-T--I----A	---Q-	QS-L-QP--HDIVRH--RL-R-L----KR--
	Chlorobium tepidum	66576257	--V--L---T--P----K	-FA-I	AEKKLIRIPSAES-S-A-R-GV----IERR-
	Chlorobium chlorochromatii	78188544	---S-L------L--H-L	--D--	ARQ--K-LSGDIVAE--RQMVAL-HQ-E-A-
	Chlorobaculum limnaeum	2501894174	--V--L---K--P--R-S	-FA-I	AGKKLIR-PSAE--S-A-R-G----KIERR-
	Prosthecochloris ethylica	198911889	--I--L-I-T--L----A	---Q-	DT-L-RPLP-DLVKE--RQ-H-L-HERKQA
	Chlorobaculum thiosulfatiphilum	139457401	--V--L---K--P--R-R	-FA-I	AEKKLIR-PSAE--S-A-R-G----------
	Prosthecochloris marina	167392930	--A--L---T--L--E--	-SKQI	-S-VCKK-HSEI-RS--KL-LDLA-V-QR--
	Candidatus Chlorobium masyuteu	166807448	-----L------I----A	---QT	ASR-LI----AEIAR--RV-NDL-R--E-A-
	Chlorobium sp. KB01	083694608	-----L------I----A	---QT	ASR-LI----AEIAR--RV-NDL-R--E-A-
	Chlorobium sp. N1	131354373	---S-L-----------L	---Q-	EA-V-RR-E-KE-AR---E-IRL--D--RSY
***Chloroherpetonaceae* fam.nov**	*Cand. Thermochlorobacter aerophilum*	RFM24603	--I--L---TF-K--N-Y	A-ELL	DR--WQP--DK-KQA--RK-H-LSFF-KQK-
Ignavibacteriae	*Melioribacter roseus*	397690794	--VS-L---TY-E--N-K		AEKMPEH-THEIRKK-NNM-RILS-K-RN--
	Ignavibacterium album	385809643	--FS-L---TY-E--N-K		AISLPGK-EVNERKK-NNM-RILSDK-KNA-
**Other Bacteria**	*Caldithrix abyssi*	493988209	---------TY-D-K--T		-FKMKPK-----KKL-NR-MI-M-Q--KFA-
	Thermovibrio ammonificans	319789539	---G-L---PY-R-R---		AARL-NS-T--EKRR-GEL-R-V-TE-SV--
	Syntrophus aciditrophicus	85860140	--V--L---PY------P		AAAMPDQ-S-DEKKK-AE--RTL-T--REA-
	Geobacter metallireducens	404495733	--V-SL---PY-R-E--A		AARMEGQ-DGK-IR--AE--R-V----RRS-
	Geobacter bemidjiensis	197118030	-----L---PF-Q----P		AATMTPQ-H-K-IKE-AEA-RVLS---KSDY
	Geobacter daltonii	222055074	---------PF-P-S--P		AATMEGH-HSS-IKE-AKA-RKLS-E-KKS-
	Syntrophobacter fumaroxidans	116747616	---T-L---PF-P----P		AADMPGRIPGDETKK-ARL-QD--T--RRQ-
	Geobacter uraniireducens	148263556	--V------PF-P-T--P		AATMGDH-RSS-IKE-AKA-RKLS-E-KKAY
	Candidatus Nitrospira defluvii	302037249	--FS------Y-S----A		AARLEDQIS-A-IRQ--KA-S-LSRT--LA-
	Thermodesulfovibrio yellowstonii	206890255	IGFS-L---PY-K--F-K		ASEMTEQIPENIKRQ-ADC-I-V-K--KL-Y
	Acetivibrio cellulolyticus	497937468	ISFSAM---KY-Q-K--P		AAEFSNQIS-KKKDE--EV-I-LSSK-TK--
	Eubacterium eligens	238917088	VHFYEM-I-KY-R-K---		ADKMKEQ--DT-K-E---V-LALEKAQSL-Y
	Clostridium clariflavum	374295594	INF-AM---KY-Q-K--P		AANFENQ-S--KKEE--N--L-LSA-MTR--
	Nitrospina gracilis	491153350	--FT-L---TY-P-K--T		AFAMKNN-PKTDKKE-NRR-T-L-RD--LT-
	Thermodesulfobacterium geofontis	337288075	S--NWL-I-PF-L----F		AEKLNSKIPQ-EIEN-KK---NLI-G-REK-
	Thiothrix nivea	488795658	VGFGHL-I--Y---E--K		AA-LPGQ--EP-KKQ--QT-H----LMKQ-W
	Rudanella lutea	518831224	--IS-L---TY-E--N-H		ALSI-PI-PGN-RAE--KM-HILSDK-RRA-
	Flexithrix dorotheae	522017869	-DIS-L---TY-E--N-E		AIELEGV-PMK-RNQ--KM-RGLSHK-KRA-
	Runella slithyformis	338213581	--VS-L---TY-E--N-L		ALEIKPV-PLSQRAE--KM-HILSDK-RRA-
	Marivirga tractuosa	313674192	--VS-L---TY-E--N-D		ADEMDEV-PMK-RNE--KM-RSLS-K-KRA-
	Cyclobacteriaceae bacterium	499123756	--VS-L---TY-E-AN-R		AVEMEGV-PMKKRNE--KM-RILS-K-RRQ-
	Parabacteroides goldsteinii	494928094	-DISQL----Y-E----Q		ALKIAHS-D-K-KHA--QQ-LD-SD--LHA-
	Segetibacter koreensis	517440681	-DIS-L---TY-E-AN-L		ATEMKEV-PVN-RHE-NKK-RNLSYM-MS-H

Figure S38

Partial sequence alignment of the MiaB-like tRNA modifying protein, showing a 5 aa insertion (boxed) that is specific for members belonging to the family *Chlorobiaceae*.

			262		312
**Family *Chlorobiaceae***	*Chlorobium limicola*	WP_041465658	EGMVSFKALLFLPKEAPPELLYRQSE	LEN	RGPQLYVKKVMIQSECRDLLPE
	Chlorobium phaeovibrioides	WP_126383222	--A----------QN--M------G-	---	K------------N--------
	Chlorobium luteolum	WP_011358007	--A-----I--------M------G-	---	K------------H--------
	Candidatus Chlorobium masyuteum	WP_166808743	-------------S----------G-	---	----------L--N--------
	Chlorobium ferrooxidans	WP_040433047	-------S-----S----------G-	---	----------L--N--------
	Chlorobaculum limnaeum	WP_069809908	--A--------I-S---M----N-GA	--K	----------L--N--------
	Pelodictyon phaeoclathratiforme	WP_012508305	----T--------------M----G-	---	H---------L--N--------
	Prosthecochloris vibrioformis	WP_068867009	--V--------I-Q---MDM--Q-GA	---	H---------L--N--K-----
	Chlorobaculum tepidum	WP_010932510	--A--------I-S---M----N-GA	--K	----------L--H--------
	Chlorobaculum thiosulfatiphilum	WP_139457868	--A--------I-S---MD---N-GA	--K	----------L--H--------
	Chlorobaculum parvum	WP_012502101	--V--------I-S---M----Q-GA	--K	----------L--H--------
	Prosthecochloris ethylica	WP_175187146	--A--------------MDMM-Q-GA	---	H-----AH-----H--K-----
	Prosthecochloris aestuarii	WP_012505491	--V--------------MDM--Q-GA	---	H------R--L--H--K-----
	Chlorobium phaeobacteroides	WP_011745048	--K-C--------E------M---GD	--S	----------L--Q--------
	Prosthecochloris marina	WP_110023524	--V-N------------MDM--Q-GA	---	H------R-----H--K-----
	Chlorobium sp. N1	WP_131353572	--A-----I--------M------G-	--Q	K------------H--------
	Chlorobium sp. KB01	WP_076790368	-------S-----S----------G-	---	----------L--N--------
***Chloroherpetonaceae* fam.nov**	*Chloroherpeton thalassium*	WP_012501118	--R----S------S--MG--HLKD-		KSL----N--L--DD-K-----
**Larger *Ignavibacteriae* Clade**	*Chlorobi bacterium OLB7*	KXK52223	--TFEYF----I-SR--MD-FT-EGV		H-LN----R-F-MEDAK----T
	Ignavibacterium album	WP_014558883	DAPIQ-N----I--RSYEFWRWSKDD		Y-LD---RR-L--HQNK-----
	Melioribacter roseus	WP_041356047	DAPIQ-N----I--KSDEFFWIDRDN		Y-LD---R--L--HKNK-----
**Other Bacteria**	*Williamsia sterculiae*	WP_076482150	--SFE-Q----I-SQ--YD-FM-DRA		H-V-----R-F-MDD-D--M--
	Mycobacterium neoaurum	WP_019514778	--TFEYQ----I-SQ--FD-FN-DGN		T-V-----R-F-MGD-DE-V--
	Micrococcus paurometabola	WP_013128275	--TFEYQ----I-TMP-FDMF--NTK		P--A----R-F-MDH-EA---G
	Maledivibacter halophilus	WP_079492068	D-L-RYN-I--I-ENM-FDYYTKEY-		K-LE--SNG-L-MNK-Q----D
	Chryseobacterium mucoviscidosis	WP_079350953	D-A-VYN-I--I-ENT-FDYYTKEY-		K-LE--SNG-L-MDK-G----D
	Pedobacter himalayensis	OOK78077	--KQEYTS--YI-AQ--WDMWN-DHK		H-LK---QR-F-MDDAEQFM-N

Figure S39

Partial sequence alignment of the molecular chaperone HtpG protein, showing a 3 aa insertion (boxed) that is specific for members of the family *Chlorobiaceae.*

Group	Organism	Accession	114		156
**Family *Chlorobiaceae***	*Chlorobium limicola*	WP_012466322	VEKAITPATKAVVAIPMWAPPK	[ ]	MVELAAICDRHSIMLIEDAAQ
	Chlorobaculum limnaeum	WP_069809335	-------K-----------A--	[ ]	-D-I-E--E-RG-T-------
	Chlorobaculum parvum	WP_012502337	-------K-----------A--	[ ]	-D---E--E-RGVT-------
	Chlorobaculum thiosulfatiphilum	WP_139456487	-------K-----------A--	[ ]	-D-I-E--E-RGVT-------
	Chlorobaculum tepidum	WP_010932823	-------K-----------A--	[ ]	-D-I-E--E-RGVT-------
	Chlorobium luteolum	WP_011357713	-------K------V----S--	[ ]	-D---E--R--NLT-V-----
	Pelodictyon phaeoclathratiforme	WP_012508220	-------------------S--	[ ]	-D--S-L-RERGLI-------
	Chlorobium ferrooxidans	WP_006365902	-------------------S--	[ ]	-D-IS---RK-NLI-------
	Chlorobium phaeobacteroides	WP_011745346	------S------------S--	[ ]	-D------QSNNLI-------
	Chlorobium phaeovibrioides	WP_126383305	-QQ---EK-----VV----A--	[ ]	-K--QD--T-RG-I-------
	Prosthecochloris aestuarii	WP_012505602	--R-LSSK-------------Q	[ ]	-D--S-L----GLV-------
	Prosthecochloris ethylica	WP_114607013	--R-V-GR------V------R	[ ]	-D---EV-R--ELV-------
	Chlorobium sp. N1	WP_131353344	--R----K------V----S--	[ ]	-D--E---G--NLT-V-----
	Candidatus Chlorobium masyuteum	WP_166808209	-------E-----------S--	[ ]	-D-IS---RK-NLI-------
	Chlorobium sp. KBO1	WP_076789499	-------------------S--	[ ]	-D-IS---RK-NLI-------
***Chloroherpetonaceae* fam.nov**	*Chloroherpeton thalassium*	WP_012499985	I-A----K--VI-PVHLYGQ-A	A	LD-IHQ-AEK-GLEV------
	Cand Thermochlorobacter aerophilum	RFM23378	I-AE---Q---ILPVHLYGQAA	E	IDKIVE-AT--G-AV---N--
**Larger *Ignavibacteriae* Clade**	*Chlorobi bacterium OLB5*	KXK50680	ISRLV--K-R---VVHYGGIAC	R	-D-IKQ-T--YGLY-------
	Chlorobi bacterium OLB4	KXK03854	I--H---E---IIPVHLFGL-V	A	-DIILE-AR-YKLKVV--S-
	Ignavibacterium album	WP_014561297	--A---SR---I--VHLYGQ-A	D	-DP-RE-SKAKN-H-V--C--
	Melioribacter roseus	WP_014856915	I--K---K---IIPVHLYGQSA	E	-D-IMEVARK-NLYV---G--
	Cand Kapabacteria thiocyanatum	OJX57644	-RA----R-R-I-PVHLFGQSA	D	-DA-V--ATEAG-PIV-----
**Other Bacteria**	*Salinicoccus albus*	WP_020008201	---IIVNLYGQSA	K	-D--VD--N-YGVPIV----E
	Corynebacterium aquilae	WP_075728003	--A---DK----MPVHLYGL-A	N	-PA-RE---KRGL--F--S--
	Nitrosospira briensis	WP_051536689	-RQ-L--Q----I-VHLFGE-V	D	LAP-QQF-ES-GLS-------
	Flavobacterium silvaticum	WP_169525655	-K-----K-R--L-VHLYGRLA	L	-D--D---KQNNLL-------
	Confluentibacter lentus	WP_100612968	-------K----MVVQ-CGSMG	D	-DA-QS--KAYNVL-----C-
	Luteibaculum oceani	WP_147014132	--SK---K---II-VHIYGLTS	N	LKR-RE---KKG-I------E
	Confluentibacter flavum	WP_106659310	-------K----MVVQ-CGSMG	N	-DA-QR--NA-NVF-----C-
	Muriicola jejuensis	WP_163691610	-R-----K---I-PVHLFGQCA	P	-D--LE-AEE-GLF----N--
	Pontibacter burrus	WP_163915187	---L--SK---I-VVHLYGQAC	E	-D-ILQL-E-Y-LK-------
	Galbibacter marinus	WP_008991889	-------N--VIMPVH-CGSMA	R	LK--K----K-GLL-L---C-
	Mariniflexile fucanivorans	WP_132219451	-------K---IMVVH-CGSMA	N	-DA-QN--SK--LL-----C-
	Prolixibacter bellariivorans	WP_025864104	I------R----LL-QVFGSMG	R	-D-IVEV-RKNNLK------
	Fulvivirga imtechensis	WP_009581965	-KE----R----I-VHLYGL-C	R	-D-IM--A--YN-KV---C--
	Chitinophaga pinensis	WP_148230802	I------K---I-PVHIYGQCA	D	-DAIM--AEK-GLHV---T–

Figure S40

Partial sequence alignment of the DegT/DnrJ/EryC1/StrS family aminotransferase protein, showing a 1 aa deletion (boxed) that is specific for the family *Chlorobiaceae.*

Group	Species	ID	238		279
**Family *Chlorobiaceae***	*Chlorobium phaeoclathratiforme*	194336576	KKLKIITPHTVGSYELVG		GKTSSLLLVRDESAFWQKSRCLVI
	Chlorobium ferrooxidans	493409890	-RI--V------------		--------I---N-----------
	Chlorobaculum tepidum	21674004	R-HV-----K-------		--------I-N-AE--K-------
	Chlorobium chlorochromatii	78188793	QS-T------R-----A-		-------VI-N-RE----------
	Chlorobium phaeobacteroides	119357337	-N---V----R-----A-		-N---M--I-N-NE--K------L
	Prosthecochloris aestuarii	194333827	-N---V----TD---MI-		-ES-A---I---NE--K-------
	Chlorobium limicola	189346919	QN--------T-----A-		-S------INN-QE--K-------
	Chlorobaculum limnaeum	2501894719	NR-H-V----K-------		-----I--I-N-VE--K-----I-
	Prosthecochloris marina	110021887	-N-R------K-------		-R------IT---E--K-------
	Chlorobaculum thiosulfatiphilum	139456507	SR-H------K---T---		----A---I-N-AE--K-----I-
	Prosthecochloris vibrioformis	139626512	-S-R-V---SID----IE		-GN-----IK--NE--R-------
	Chlorobium sp. KB01	076789444	-RI--V------------		--------I---N-----------
***Chloroherpetonaceae* fam.nov.**	*Chloroherpeton thalassium*	193216023	QVELL-S-SKD---IKK	D	-ENA-V-QIK-AKE--K-AK----
**Other Bacteria**	*Porphyromonas gulae*	517795839	--ANVL-S-PAS----IR	G	TDKMLT-RIK-PQ---KN-KI---
	Porphyromonas gingivalis	334146329	--ANVL-S-PAS----IR	G	TDKMLT-RIK-PQ---KN-KM---
	Alistipes shahii	479187696	--ATLV-S-PE------T	D	ANKVVEK-IITDPVRFWE-SKIL-
	Odoribacter laneus	496428340	--A-VLSV-PQD--A-EA	G	EDGMLS-T-K--NS--KQTKY--V
	Bacteroides sp. HPS0048	488622481	-RAELL-T-P-------K	D	D-GQLT-KITNPKE--SV--Y---
	Bacteroides massiliensis	511024358	-RAELL-T-PA------K	D	A-GQLT-KITNPTE--SV--Y---
	Flexithrix dorotheae	522022264	--AEL--S-PE---KIE-	S	D-KV-K-VIL-PEK--NS-KY--V
	Bacteroides vulgatus	150004391	-RAELL-T-PA------K	D	A-GQLT-KITNPTE--SV--Y---
	Prevotella ruminicola	294674538	-DA-VL-S-PAS--R-TA	D	ANKQLI-RIN-PI---ST-KY---
	Porphyromonas somerae	516662532	-A-VL-E-PSS----IK	D	-NKNMI-KIK-VDS--SLG-N--V
	Odoribacter splanchnicus	325281781	--A-LLSV-PAD--T-ET	G	EDGNMT-KIN--NN--KQTKY--V
	Coprobacter fastidiosus	550260015	--A--L-N-PE---S--K	N	D-GLLT-VIS-PQN--SL-KY--V
	Bacteroides barnesiae	517541810	-RAELL-N-PS------K	D	D-GQLT-KITNPKE--SV--Y---

Figure S41

Partial sequence alignment of the biogenesis of lysosome-related organelles complex 1 subunit 2 protein, showing a 1 aa deletion (boxed) that is specific for the family *Chlorobiaceae.*

Group	Species	Accession	234		268
Family *Chlorobiaceae*	*Chlorobium phaeoclathratiforme*	194335275	GRGKVVIRARAIVE	I	TPKNSRESIVVTELPYQVNK
	Chlorobium luteolum	78185988	-----------LI-	V	-Q--G---------------
	Chlorobaculum parvum	193211771	-----------V--	V	-Q--G----I----------
	Prosthecochloris aestuarii	194332984	-----------V--	V	-Q--G---------------
	Chlorobium limicola	189345659	-----------L--	V	-Q--G----I----------
	Chlorobium ferrooxidans	493411439	-----------L--	V	-Q--G----I----------
	Chlorobaculum tepidum	21672982	-----------V--	V	-Q--G----I----------
	Chlorobium chlorochromatii	78188072	-----------L--	V	-Q--G----I----------
	Chlorobium phaeovibrioides	145218988	-----------L--	V	-Q--G---------------
	Chlorobium phaeobacteroides	119358427	-----------L--	V	-Q--G----I----------
	Chlorobaculum thiosulfatiphilum	139456349	-----------L--	V	-Q--G----I----------
	Prosthecochloris marina	110024210	---R-------V--	V	-Q--G----I----------
	Chlorobaculum limnaeum	2501895269	-----------V--	V	-Q--G----I----------
	Chlorobium sp. N1	131355716	-----------L--	V	-Q--G---------------
	Chlorobium sp. KB01	076791893	-----------L--	V	-Q--G----I----------
	Chlorobaculum sp. 24CR	129050649	-----------L--	V	-Q--G----I----------
	Candidatus Chlorobium masyuteum	166807315	-----------L--	V	-Q--G----I----------
***Chloroherpetonaceae* fam.nov.**	*Chloroherpeton thalassium*	193213726	----I-L--KVQ--	V	----G----II---------
	Cand. Thermochlorobacteriaceae GBChlB	KER09680	----LT---KVNI-	V	N---D--T------------
	Chlorobium sp. 445	PI047609	----IT----VKI-		EN--G--A------------
	Cand. Thermochlorobacter aerophilum	RFM25354	----IT---KVKI-		EN--G---------------
**Larger *Ignavibacteriae* Clade**	*Chlorobi bacterium OLB5*	KXK50861	----II---K-GI-		VG-GD----II---------
	Melioribacter roseus	397690040	----IT-----NI-		-L--D--N--I---------
	Ignavibacterium album	385808592	---RILL--K-N--		-L--D--N-II--I------
**Other Bacteria**	*Cronobacter sakazakii*	213200884	----IY-----E--	V	D---G--T-I-H-I------
	Thiocapsa roseopersicina	093027529	---RA-----TM--	V	QKRSG--A------------
	Arsenophonus nasoniae	CBA76530	-----Y-----NI-	V	DD--G--T-I-N-I------
	Providencia heimbachae	068444842	-----Y-----E--	V	DE--G--T-I-H-I------
	Plesiomonas shigelloides	039045661	-----YL----E--	V	DE--G----I-H-I------
	Thiocapsa imhoffii	200388422	---R--M---TL--	V	QKRSG--A------------
	Mariprofundus ferrooxydans	009850567	---S-TM-------	V	N-RTH-Q-LII---------
	Cronobacter dublinensis	161586000	----IY-----E--	V	D---G--T-I-H-I------
	Dickeya undicola	123249807	-----Y-----E--	A	D--SG--T-I-H-I------
	Pedobacter saltans	325103800	---RI-M--K-EI-		SQS-E--K-I---I------
	Brucella canis	373088901	---S-IM-G--TI-		PMRGD--A-II--I------
	Balneola vulgaris	516845266	----ITL----NT-		ELRGG--Q--I--I------
	Gracilimonas tropica	521071534	----ITL----NT-		ELRGN--Q--I--I------
	Pedobacter arcticus	515827893	---R-MM-GV-EI-		-LP-D--R-I-S-I------
	Thermophagus xiamenensis	498214077	---RI----KTEI-		SD-DG--R--I--I--M---
	Joostella marina	495891560	---RI-M--K-HN-		EV-G--C-----I------
	Zhouia amylolytica	568804980	---R--L--K-EI-		EV-GK-C-----I------
	Galbibacter marinus	496279386	---RI-M--K-RI-		EVKG--C-I---I------
	Hymenobacter norwichensis	551195584	---R--V--K-H--		-LDSGK-Q-----I--M---
	Psychroflexus torquis	408492625	---R--M--KSS--		EV-G--C-II--I------
	Rhodothermus marinus	345302554	---RIL----IHE-		EIRPG-MAL-I--I------
	Flavobacterium cauense	559195123	---RI-M--K-NF-		EVDG----I---I------
	Marinilabilia salmonicolor	498348651	---RI----KSEI-		ADN-G--K--I--I--M---
	Psychroflexus tropicus	517868593	---R--M--K-DI-		EIKG--CM----I------
	Flavobacterium saliperosum	559200282	---RI-M--K-NF-		EVDG----I---I------
	Anaerophaga thermohalophila	498104611	---RI----KTEI-		-D-SG--R-II--I--M---
	Arcticibacter svalbardensis	510820181	---R-----K-LI-		-YGHD--R---N-I---I--
	Flavobacterium indicum	383450158	---RI-M--K-GF-		EV-G----I---I------
	Psychroflexus gondwanensis	489533711	---R--M--KSS--		EV-GK--LII--I------
	Anaerophaga thermohalophila	515147163	---RI----KTEI-		-D-TG--R-II--I--M---
	Muricauda ruestringensis	344203768	---R--M--K-TI-		EVQGK-------I------
	Rhodothermus marinus	268317740	---RIL----IHE-		EIRPG-MAL-I--I------
	Aequorivita sublithincola	390955544	---R--M--K-TF-		EVQG--C-I---I------
	Cellulophaga lytica	325287312	---R--M--K-TF-		EVQG--------I------
	Flavobacterium rivuli	519055916	---RI-V--KTSF-		EVDG--C-I---I------
	Polaribacter franzmannii	517774671	---RI-M--K--I-		EVKG--C-I---I------
	Alistipes putredinis	490458409	---R--M---TEI-		HTASG--C--I--I--M---
	Amorphus coralli	517527837	---S-TM-GKVA--		-TGRD--ALII--I------
	Azorhizobium caulinodans	158423952	---SIMM----TI-		-VRKE--A-II--I------
	Brucella abortus	145308239	---S-IM-G--TI-		PMRGD--A-II--I------
	Gluconobacter oxydans	58001765	---SIPV----EI-		EIRKD-QA-II--I------
	Afipia birgiae	518026837	---SI-M-GKVNI-		-IRKD--A-I-S-I------
	Acetobacter tropicalis	493605115	---AII-----E--		EIRKD-KA--I--I------
	Bacillus amyloliquefaciens	566079334	---SIT---K-EI-		ETSSGK-R-I----------
	Bacillus licheniformis	117654167	---SIT---K-QI-		ETSSGKQV-II--I------
	Cyprideis torosa	CAD7231638	-----------N--		SH--D----II----F----

Figure S42

Partial sequence alignment of the protein DNA gyrase subunit A, showing a 1 aa insertion (boxed) that is specific for members belonging to the family *Chlorobiaceae*. The CSI is not present in any other bacterial species except for the nine other bacteria as indicated above, in the top 500-BLASTp hits.

Group	Organism	Accession	30		64
***Chloroherpetonaceae* fam.nov.**	*Chloroherpeton thalassium*	WP_012500714	AIRASQLGYKTCIIEKE	Q	TLGGVCLNWGCIPTKSL
	Cand. Thermochlorobacter aerophil	RFM24380	------------V----	P	-----------------
	Cand. Thermochlorobacter GBChlB	KER08989	----A---F---V----	P	-----------------
	Chlorobium sp. 445	PI048610	------------V----	P	-----------------
**Family *Chlorobiaceae***	*Chlorobaculum tepidum*	WP_010932967	--H-ARY-L----V--A		V-----V--------A-
	Pelodictyon phaeoclathratiforme	WP_012508516	-L--AKA-M-V-L---G		A-----V--------A-
	Chlorobium ferrooxidans	WP_006366348	-L-GAKA-M-V-----A		A-----I--------A-
	Chlorobium phaeobacteroides	MBC8524564	-VK-A-S-LQV--V--G		AI----V--------A-
	Prosthecochloris ethylica	WP_175186716	-L--AGE-LRV--V--G		P-----V--------A-
	Chlorobaculum tepidum	AAG12404	--H-ARY-L----V--A		V-----V--------A-
	Prosthecochloris aestuarii	WP_012505992	-V--ARK-LTV----RG		A-----V--------A-
	Prosthecochloris vibrioformis	CAB06298	-LH-ARH-M-V-LV--R		S-----V--------A-
	Chlorobaculum parvum	WP_012501976	-LH-ARH-M-V-LV--A		S-----V--------A-
	Candidatus Chlorobium masyuteum	WP_166808580	-L-GAKA-M-V-----A		A-----I--------A-
	Chlorobi bacterium NICIL-2	KXB98049	----A---M--IC--RD		R------------S-A-
	Prosthecochloris sp. ZM	WP_114617058	-V--ARK-LTV----RG		A-----V--------A-
	Chlorobium sp. KB01	WP_076789414	-L-GAKA-M-V-----A		A-----I--------A-
	Prosthecochloris sp. ZM_2	WP_114607234	-L--AGE-LRV--V--G		P-----V--------A-
**Larger *Ignavibacteriae* Clade**	*Ignavibacteria bacterium*	MBK9333167	--------F--A----D		R---I----------A-
	Chlorobi bacterium OLB7	KXK57232	--------L--A-V-RD		R---I----------A-
	Chlorobi bacterium OLB5	KXK46273	--------L--ALV-TA		R---I--------S---
	Chlorobi bacterium OLB6	KXK33210	--------L-VACV-RD		R------------S---
**Other Bacteria**	*Bizionia paragorgiae*	WP_092133119	--------F--A-----		S--------------A-
	Chryseobacterium indologenes	HA026821	----A---F--A-----		N---I----------A-
	Salinimicrobium marinum	WP_189603939	--------F--A-----		S--------------A-
	Salegentibacter salinarum	WP_079714361	--------F--A-----		S--------------A-
	Muricauda aquimarina	WP_127139765	--------F--A-----		S--------------A-
	Stylophora pistillata	PFX17179	--------F--A-----		S--------------A-
	Aquimarina celericrescens	MBQ0733288	--------F--A-----		S--------------A-
	Salinimicrobium sediminis	WP_097057020	--------F--A-----		S--------------A-
	Flavobacterium johnsoniae	WP_121360554	--------F--A-V---		N--------------A-
	Winogradskyella jejuensis	WP_073086302	--------F--A-----		S--------------A-
	Cellulophaga tyrosinoxydans	WP_084061203	--------F--A-----		S--------------A-
	Tenacibaculum dicentrarchi	WP_101902757	--------F--A-----		N---I----------A-
	Bizionia myxarmorum	WP_148403574	--------F--A-----		S--------------A-

Figure S43

Partial sequence alignment of the protein hypoxanthine phosphoribosyltransferase protein showing a one amino acid insertion (boxed) that is exclusively shared by all members of the *"Chloroherpetonaceae"* clade.

			30		64
***Chloroherpetonaceae* fam.nov.**	*Chloroherpeton thalassium*	WP_012500714	AIRASQLGYKTCIIEKE	Q	TLGGVCLNWGCIPTKSL
	Cand. Thermochlorobacter aerophilUm	RFM24380	------------V----	P	-----------------
	Cand. Thermochlorobacter GBChlB	KER08989	----A---F---V----	P	-----------------
	Chlorobium sp. 445	PI048610	------------V----	P	-----------------
**Family *Chlorobiaceae***	*Chlorobaculum tepidum*	WP_010932967	--H-ARY-L----V--A		V-----V--------A-
	Pelodictyon phaeoclathratiforme	WP_012508516	-L--AKA-M-V-L---G		A-----V--------A-
	Chlorobium ferrooxidans	WP_006366348	-L-GAKA-M-V-----A		A-----I--------A-
	Chlorobium phaeobacteroides	MBC8524564	-VK-A-S-LQV--V--G		AI----V--------A-
	Prosthecochloris ethylica	WP_175186716	-L--AGE-LRV--V--G		P-----V--------A-
	Chlorobaculum tepidum	AAG12404	--H-ARY-L----V--A		V-----V--------A-
	Prosthecochloris aestuarii	WP_012505992	-V--ARK-LTV----RG		A-----V--------A-
	Prosthecochloris vibrioformis	CAB06298	-LH-ARH-M-V-LV--R		S-----V--------A-
	Chlorobaculum parvum	WP_012501976	-LH-ARH-M-V-LV--A		S-----V--------A-
	Candidatus Chlorobium masyuteum	WP_166808580	-L-GAKA-M-V-----A		A-----I--------A-
	Chlorobi bacterium NICIL-2	KXB98049	----A---M--IC--RD		R-----------S-A-
	Prosthecochloris sp. ZM	WP_114617058	-V--ARK-LTV----RG		A-----V--------A-
	Chlorobium sp. KB01	WP_076789414	-L-GAKA-M-V-----A		A-----I--------A-
	Prosthecochloris sp. ZM_2	WP_114607234	-L--AGE-LRV--V--G		P-----V--------A-
**Larger *Ignavibacteriae* Clade**	*Chlorobi bacterium OLB7*	KXK57232	--------L--A-V-RD		R---I----------A-
	Chlorobi bacterium OLB5	KXK46273	--------L--ALV-TA		R---I--------S---
	Chlorobi bacterium OLB6	KXK33210	--------L-VACV-RD		R------------S---
	Ignavibacteria bacterium	MBK9333167	--------F--A----D		R---I----------A-
**Other Bacteria**	*Flavobacterium columnare*	WP_213068832	--------F-VA-----	N	S---I----------A-
	Bizionia paragorgiae	WP_092133119	--------F--A-----		S--------------A-
	Chryseobacterium indologenes	HAO26821	----A---F--A-----		N---I----------A-
	Salinimicrobium marinum	WP_189603939	--------F--A-----		S--------------A-
	Salegentibacter salinarum	WP_079714361	--------F--A-----		S--------------A-
	Muricauda aquimarina	WP_127139765	--------F--A-----		S--------------A-
	Stylophora pistillata	PFX17179	--------F--A-----		S--------------A-
	Aquimarina celericrescens	MBQ0733288	--------F--A-----		S--------------A-
	Salinimicrobium sediminis	WP_097057020	--------F--A-----		S--------------A-
	Flavobacterium johnsoniae	WP_121360554	--------F--A-V---		N--------------A-
	Winogradskyella jejuensis	WP_073086302	--------F--A-----		S--------------A-
	Cellulophaga tyrosinoxydans	WP_084061203	--------F--A-----		S--------------A-
	Tenacibaculum dicentrarchi	WP_101902757	--------F--A-----		N---I----------A-
	Bizionia myxarmorum	WP_148403574	--------F--A-----		S--------------A-

Figure S44

Partial sequence alignment of the protein dihydrolipoyl dehydrogenase protein containing a 1 aa insertion (boxed) that is specific for members of the *"Chloroherpetonaceae"* clade.

Group	Species	Accession	236	(insertion)	280
***Chloroherpetonaceae* fam.nov.**	*Chloroherpeton thalassium*	WP_012499003	TEPVNIGNPDEITILDFAKEVQ	TIVK	ELTGKDTEIIFKELPSDDP
	Cand. Thermochlorobacter aerophilum	RFM24898	H----L---S------------	A--R	-----EIP-VH-P--A---
	Chlorobium sp. 445	PIO47888	H--------S------------	A--R	--I--EIP-VH-P--A---
	Cand. Thermochlorobacteriaceae GBChlB	KER10278	H--------S-----E------	--M-	-F--REA--T--P--A---
**Larger *Ignavibacteriae* Clade**	*Chlorobi bacterium NICIL-2*	KXB98589	V----L-----V-V-EL--QIR		D-V-SSSP-E-HP--E---
	Chlorobi bacterium OLB7	KXK55332	ND---------L-M-EL-Q-IK		N---SSSP-E-RA--K---
	Ignavibacteria bacterium	MBD1209187	-D----------M--L---IL		-I--SKS--V--P--E---
**Other Bacteria**	*Rhodocaloribacter litoris*	NHZ74744	V-----------S-----R--V		R---SRS--V--P--Q---
	Rhodothermus marinus	HEK45053	VG-------E--S--E----II		----SNS-----P--A---
	Rhodohalobacter barkolensis	WP_101072079	S----L---E----------II		-I--SKS-VV-----E---
	Candidatus Kapabacteria bacterium	MBL7976002	SY----------SM--L---II		----TTSS-E--D--Q---
	Rubricoccus marinus	WP_094545675	HD---------VS--E--Q--V		A---SSS--VH----A---
	Aliifodinibius sediminis	WP_142714759	-D-------Q-----E--R-II		----S-S--T-E---K---
	Terrimonas ferruginea	WP_028787551	AM----------SL------IR		Q-S-ERS--V--P--V---
	Nonlabens spongiae	WP_085767182	SD-------H----------IV		K---T-QK----D--Q---
	Gramella portivictoriae	WP_026913634	SD------------------II		K---T-QK-V--D--Q---
	Fodinibius salinus	WP_148897875	---------Q--S--E----II		D---S-SK-T-E---K---
	Patiriisocius marinus	WP_151672747	-H----------------Q-II		K---T-QK-V-----K---
	Rhodohalobacter halophilus	WP_069132987	VY-------E----------II		DI--SNSK-V-----E---
	Aliifodinibius halophilus	NGP89105	V--------------E----II		---ES-S--T-E---K---
	Raineya orbicola	WP_101358019	PY-----------L-E----I-		----SKSK-V--P--K---
	Rubrivirga marina	WP_095511589	-D---V-----VS-KE--E--V		A---SSST-TYEP------
	Gramella forsetii	WP_011711129	S-----------S-----D-II		R---T-QK-V-E---K---
	Gramella aestuarii	WP_156277031	SD-------N--S-----R-II		K---T-Q--V-----Q---
	Salinibacter altiplanensis	WP_103020725	-D------------QE--E-II		-V--T-S--TYEP------
	Microscilla marina	WP_045114667	AH----------S-K---E-II		K---T-QK--Y-D--K---
	Salinivenus iranica	WP_103038273	-D------------KE--E-II		-I--S-SP-TYE---K---
	Candidatus Moduliflexus flocculans	GAK54610	SD-------R-MS--Q--EKIK		AI--TSS--V-EP--V---
	Jiulongibacter sediminis	WP_055143938	AQ-------N----A---E-II		K---T-QKV-Y-D--K---
	Chryseotalea sanaruensis	WP_127121542	HL-------------Q--E-II		K---T-QK-------K---
	Gramella gaetbulicola	WP_108170953	S--------N--S-----D-II		K---T-QR-VY----Q---
	Telmatocola sphagniphila	WP_213494753	H----L-----V---E-----L		--S-SKS--VY-P--Q---
	Candidatus Fermentibacteria bacterium	MBD3368767	-G---V-----M---E--EF-R		K-G---SR-VHR---K---
	Gemmata obscuriglobus	WP_010047942	HD---L---A-----E--E-IK		K-A-SKS--V--P--Q---
	Candidatus Brocadia sinica	WP_052562761	YD-------E----HQL---IL		A---SKSR----T--V---

Figure S45

Partial sequence alignment of the protein SDR family oxidoreductase, showing a 4 aa insertion (boxed) that is specific for members of the "*Chloroherpetonaceae*" clade

			164		203
***Chloroherpetonaceae* fam.nov.**	*Chloroherpeton thalassium*	WP_012499407	YDFRPSYQQIASQLKLIYPKT	RP	VMLALTATATEEILKDI
	Cand. Thermochlorobacter aerophilum	RFM23398	--------K--TGIAQLGLPK	--	T-----------V-E--
	Chlorobium sp. 445	PIO48802	--------K-TAG-AQLGLPK	--	T-----------V-E--
**Family *Chlorobiaceae***	*Chlorobium phaeobacteroides*	MBV5319837	H----D-LSLSQLVT-FPDLP		VA-F-----HQVQR--
	Chlorobaculum thiosulfatiphilum	WP_139456672	H----D-LALS-IPEVLPGIP		VA-F------RVQH--
	Prosthecochloris marina	WP_110024258	H----D-LSL-ALA--FPGTP		IA-F-----HKVQQ--
	Chlorobi bacterium NICIL-2	KXB98426	H------RR-GELSGALGR-P		I--------QQTRR--
	Prosthecochloris sp. HL-130-GSB	WP_198166311	H----D-RR-VRV-QA-PSNV		PV--T----NDRVVH-V
**Other Bacteria**	*Fictibacillus enclensis*	WP_061970029	H------LR-KDM-NNL--RP		-IV-------K-VTE--
	Vagococcus silagei	WP_136136718	H------LKL-ETIQV-Q--P		TIV-------P-VAQ--
	Bacillus marasmi	WP_147534659	H------RY-S-I-QH-HS-P		II--------AQVRS--
	Candidatus Gallibacteroides avistercoris	HIU54418	-------L---QIRH-LPQVP		V--------N-AI---
	Gracilimonas mengyeensis	WP_142454845	H------RE-R-S-ES-ADQ-		RW--------P-VQ---
	Ornithinibacillus massiliensis	WP_211741114	H------RS-VPT--Q-RNIP		-YM---------VIS--
	Oceanobacillus chungangensis	WP_115748595	H------RS-VPN--KLNNIP		-FV---------VIN--
	Virgibacillus dokdonensis	AUJ24938	H------RS-IH--QQLPKRP		--M-------T-VIE--
	Virgibacillus chiguensis	SHH23913	H------RS-IP--QQLPKRP		--M-------T-VIE--
	Fictibacillus solisalsi	WP_090233596	H------LR-KDM-NNL--RP		-IV-------K-VTE--
	Peptococcus niger	WP_200781888	H------R---AG-SALT-RP		IFA--------PVQA--
	Bacillus amyloliquefaciens	KJD52951	H------RH-E-F-NSLGTRP		-V--------P-VHE--
	Ornithinibacillus californiensis	WP_047983848	H------RS-VPN--R-GTIP		-IM---------VIS--
	Ornithinibacillus caprae	WP_155671718	H------RS-VP---Q-RNIP		-YV---------VIS--
	Virgibacillus subterraneus	WP_092502198	H------RS-V-N--RLSNIP		--IG--------VIS--
	Virgibacillus salinus	SDQ11862	H------RS-V-N--RLSNIP		--IG--------VIS--
	Spirochaetaceae bacterium	MBP3450048	H----E-RR-SEIRQIFPKAV		C---------TVR---
	Enterococcus canintestini	WP_095006432	H------L-L-QEI-ALPKRP		AVI-------PQVA---
	Alicyclobacillus ferrooxydans	WP_206659046	H-------S--PF-EQLDERP		-VV-------P-VTR--
	Litchfieldia alkalitelluris	WP_078547561	H------RN-K-MIDSLKD-P		TIV-------K-VIR--
	Oceanobacillus senegalensis	WP_085993971	H------RS-V-N--QFTNIP		-IV---------VIN--
	Candidatus Kurthia intestinigallinarum	HIX42122	F----E-L---EA-PKER-PI		---S----NQ-MR--
	Alkalihalobacillus bogoriensis	WP_035177688	H------MG-STF-SVLQ--P		-I---------QVQA--
	Flavobacteriales bacterium	NQY11258	-------LN-SELR--FKTVP		VM-------K-VV---
	Bacillus alkalicellulosilyticus	WP_078554038	H------RS-STYIDSLQ--P		LI---------QVQE--
	Xylanibacillus composti	WP_213413892	H------M--PKL-RQ-H-RP		-VA-F-----DKVKE--
	Oceanobacillus caeni	WP_204798664	H------RS-V-T--Q-SNIP		-FV---------VIH--
	Massilia timonae	HAK90119	H----A-IGL-AAIEA-GRPP		V--------D-VIA--
	Gracilimonas amylolytica	WP_103665834	H------RE-R-S-ES-ADN-		RWI-------P-VQ---
	Candidatus Tetragenococcus pullicola	HIY56919	H------LEL-ES--K-E-RP		AII-------PQVS---
	Bacillus paralicheniformis	WP_145654026	H------RH-E-F-NSLGTRP		-V--------P-VHE--
	Bacillus licheniformis	WP_031314645	H------RH-EGF-NSLGTRP		-V--------P-VHE--
	Oceanobacillus halophilus	WP_121204935	H------RS-V-N--QFSNIP		-FV---------VIS--
	Enterococcus faecium	WP_049143502	H------L-M-N--DQLPNRP		-IV-------VQVAA--
	Bacillus licheniformis CG-B52	EQM27666	H------RH-EGF-NSLGTRP		-V--------P-VHE--
	Ornithinibacillus halotolerans	WP_188383055	H------RS-VPN--Q-GSIP		-VM---------VIS--
	Enterococcus dispar	WP_016173413	H------L-L-QEI-ALPQRP		AVI-------PQVA---
	Paenibacillus psychroresistens	WP_155699081	H------RS-STL--MLPERP		IVA-F-----P-VVD--
	Lactobacillus paragasseri	WP_113576156	H----A-R--MDGV-S-KSNP		NI--------PSVQ---
	Oceanobacillus limi	WP_090872575	H------RS-VP---QLNNIP		-YV---------VIS--
	Virgibacillus alimentarius	WP_226371238	H------RS-VP---QLANIP		-FV---------VIS--
	Priestia megaterium	WP_026682619	H------RS-IP---QLHHAP		IIM---------VIF--
	Loigolactobacillus binensis	WP_137638398	H------L-VTAR-QELRT-P		-LV--------RVAT--
	Halobacillus faecis	WP_146815185	H------RS-VPT--K-ANLP		-LM-------Q-VI---
	Paenibacillus hemerocallicola	WP_139601900	H------MS-SRV-N--R-RP		-IA-F-----DKVKE--
	Paenibacillus mesophilus	WP_138881353	H------MS-SRV-N--R-RP		-IA-F-----DKVKE--
	Lactobacillus johnsonii	WP_180872720	H----A-R--MEGV-S-KSQP		NI--------PSVQ---
	Halobacillus karajensis	WP_074733221	H------RS-VPT--E-PNLP		-LM-------Q-VI---

Figure S46

Partial sequence alignment of the protein RecQ family ATP-dependent DNA helicase, showing a 2 aa insertion (boxed) that is found specific for members of the *"Chloroherpetonaceae"* clade

			33		78
***Chloroherpetonaceae* fam.nov.**	*Cand.Thermochlorobacter aerophilum*	RFM24133	IAIVIDQFRYDYLERFEAYY	LPATR	TSGGFKRLLKHGALFTNAHYR
	Chlorobium sp. 445	PI047328	V------L--------D-H-	VA--K	--------IEQ---L-----S
	Chloroherpeton thalassium	WP_012500094	LT----------D---DMF	--VGK	H----RK-R-Q--FMEKCF-D
Ignavibacteriae	*Ignavibacteriaceae bacterium*	MBE2278926	V-V-V--M-----QK-YPL-		GD------M-D-FNC--V--N
**Other Bacteria**	*Thermaurantimonas aggregans*	WP_218019366	VG--V--MK-E------PL-	S	KN---A---SE-FFYK----N
	Schleiferia thermophila	WP_037361302	VG--V--MK-E------PLF	S	D----R-FT-Q-FN-K----N
	Candidatus Kapabacteria bacteria	MBU3699324	VVVSF----G--PRQ---VF	P	-Q---R--M-E--T--SCR-E
	Seonamhaeicola maritimus	WP_147769213	V------M-----T---SK-		GE-----MIEE-FNCK-N-FN
	Nonlabens agnitus	WP_105981864	VG--V--M-----T--YDR-		SN------M-D-FSA--N--N
	Echinicola rosea	WP_137401383	VG--V--M--E--HK-NDRF		-D------MNE-FMMK----N
	Cecembia rubra	WP_106568470	-G--V--M-QE-FYK---R-		SE------M-D-FMMK----N
	Echinicola vietnamensis	WP_015265142	VG--V--M--E--HK-NDRF		-D------MND-FMMK----N
	Flavobacteriaceae bacterium	HAE71810	VG--V--M----IS--WNG-		SE------V-E-FN-K-N--N
	Echinicola soli	WP_141613469	VG--V--M--E--HK-NDRF		-D------MND-FVMK----N
	Echinicola shivajiensis	WP_215225323	VG--V--M--E--HK-HDR-		-E------MHE-FMMK-G--N
	Nonlabens xylanidelens	WP_104515391	VG--V--M-----S--YNR-		SD------MNE-FNL--N--N
	Ekhidna lutea	WP_089358319	VG--V--M----IS-YYSDF		ED------IEE-F-YR----N
	Cyclobacteriaceae bacterium	NOT77289	VG--V--M-QE--Y---KKF		GP------MSD-FMLK----N
	Arcticibacter tournemirensis	WP_161987516	VG-I---M-----Y-YQDK-		SE-------GQ-F--RDT--N
	Cecembia lonarensis	WP_040400614	VGL-V--M-QE-FYK--ER-		-E------M-E-FMMK----N
	Cecembia calidifontis	WP_130275039	VG--V--M-QE-FYK--ER-		SD------M-E-FMMK----N
	Algoriphagus marincola	KPQ14508	VG-IV--M-QE--Y--SDR-		SE------M-E-FMMK-G--N
	Pontibacter actiniarum	WP_025608463	VG--V--M-----Y-YWSK-		GND---K--SE-FS-R-T--N
	Nonlabens dokdonensis	WP_015360898	VG--V--M-F---T--SSR-		-N---N--IND-YQL--N--N
	Aureibaculum marinum	WP_123896104	-G--V--M-----T--YNK-		GE-------NS-YN-E----N
	Hymenobacter metallilatus	WP_125432143	VG--V--M-----Y-YWNK-		G----R---GE-FSYE----N
	Algoriphagus antarcticus	WP_086541318	VG-IV--M-Q---Y--SDR-		SD-----MM-D-FMMK-G--N
	Hymenobacter wooponensis	WP_135532231	VG--V--M-----Y-YWSK-		GN---R---GE-FSYE----N
	Pyrinomonadaceae bacterium	MBA2502343	LL--V-----------GDLF		APN-LRMM-RN--SW--TNFD
	Seonamhaeicola algicola	WP_147135211	VG--V--M-----T---SK-		GN-----MIRE-YNCK-H--N
	Hymenobacter lapidiphilus	WP_176909843	VG--V--M-----Y-YWSK-		G--------GE-FSYE-T--K
	Gramella gaetbulicola	WP_108172269	VG--V--M-----T--WDQF		EN------VND-FT-K-T-FN
	Kordia jejudonensis	WP_046756932	VG-IV--M-----TQ--NK-		SE-----M-RE-FNCK-N--N
	Mariniradius saccharolyticus	WP_040480808	VG--V--M-QE-FYK-QER-		-E------MRE-FMM--G--N
	Kordia antarctica	WP_160129351	-G--V--M-----TQ-D-K-		SE-----M-NG-YNCK-N--N
	Nibribacter ruber	WP_160691664	VG--V--M-----Y-YWDK-		SND------RE-FN-K-N--N
	Aquimarina intermedia	WP_148782777	VG--V--M-----T--DTSF		G------IS-E-FQYK---FN
	Leptobacterium flavescens	WP_163608315	VG--V--M--N--T--WSK-		GN------INE-FNCK-N--N
	Tenacibaculum jejuense	WP_095073954	VG-IV--M-----T--DSK-		GE------I-D-FSLE---FN
	Nonlabens mediterranea	MBF4982940	VG--V--M-----T--YKR-		GD------MND-FNL--N-FN
	Psychroserpens damuponensis	WP_040278820	VG--V--M-----T---NK-		SD-----MINQ-FNCK-N-FN
	Nonlabens xiamenensis	WP_124979762	VG--V--M-----T--AHR-		GE---N--VRD-FNA--H--N
	Hymenobacter gelipurpurascens	WP_088845355	VG--V--M-----Y--WSK-		GN---R---GE-FSYE----N
	Antarcticibacterium flavum	WP_139067918	VG--V--M-----T--WDK-		EE------VRN-FN-R---FN
	Rufibacter radiotolerans	AKQ47831	-G--V--M-----Y--WGKF		-ND-----VNQ-FS-Q-N--N
	Urechidicola croceus	WP_070236445	VG--V--M-----T--YDK-		SE------MNN-YNLK-T--N
	Hymenobacter psychrophilus	SDY42225	VG--V--M-----Y-YWSK-		G--------GE-FSYE-T--N
	Rufibacter glacialis	KAA6431211	-G--V--M-----Y-YWDK-		SAN------AE-FN-R-N--N
	Gramella sabulilitoris	WP_143411027	VG--V--M-----T--WGQ-		GQN-----VNN-FV-K-N-FN
	Mangrovimonas yunxiaonensis	WP_036122168	VG--V--M-----T---DR-		GD---N-MV-E-FNCK-N-FN
	Belliella pelovolcani	WP_076500269	VG--V--M-QE-FYK-QER-		-E------MND-FMM--G--N
	Saprospiraceae bacterium	MCB0575169	-G--V--M-----Y-YRDS-		GE-------AQ-FSCE-T--N
	Mesonia hippocampi	WP_183478300	VG--V--M-----T--WDK-		QAD-----INE-FV-K-N-FN
	Nonlabens marinus	WP_041497300	VG--V--M-----T--YSR-		GN------MNE-FNV--N--N

Figure S47

Partial sequence alignment of the protein alkaline phosphatase family protein, showing a 5 aa insertion (boxed) that is specific for members of the *"Chloroherpetonaceae"* clade

Group	Species	Accession	2	(boxed)	30
Order *Chlorobiales*	*Chlorobium limicola*	WP_012467025.1	KIGSIDIDRPVI		LAPMEDVTDRAFRQLCK
	Pelodictyon phaeoclathratiforme	WP_012509195.1	---T-E------		-----------------
	Chlorobium phaeovibrioides	WP_126385139.1	----L-------		-----------------
	Chlorobium sp. KB01	WP_076792104.1	-----A------		-----------------
	Candidatus Chlorobium masyuteu	WP_166807179.1	---P--------		-----------------
	Chlorobium phaeobacteroides	WP_011746071.1	R--P--------		-----------------
	Chlorobium sp. N1	WP_131356337.1	---AL-------		-----------------
	Chlorobium luteolum	WP_011357039.1	---KL-------		-----------------
	Chlorobium ferrooxidans	WP_006366291.1	---P-A------		-----------------
	Chlorobaculum tepidum	WP_164927287.1	R--T------I-		-----------------
	Chlorobaculum limnaeum	WP_069810711.1	R--T-E----I-		-----------------
	Chlorobaculum parvum	WP_012501542.1	R--P------I-		-----------------
	Chlorobaculum thiosulfatiphilum	WP_139455934.1	R--Q--------		-----------------
	Prosthecochloris vibrioformis	WP_139626091.1	Q---F-MA---M		----------S--K---
	Prosthecochloris marina	WP_110022432.1	R--KLH-E----		----------S--KI--
	Prosthecochloris aestuarii	HED30740.1	-D-FEMQ---M		-----E-S--S--KI--
	Prosthecochloris ethylica	WP_175186888.1	--GF-MK---M		-----E-S--S--KI--
	Chloroherpeton thalassium	WP_041468962.1	---Q-E-EK---		---------PS--R---
	Cand. Thermochlorobact. GBChlB	KER09073.1	---T---EQ-IV		---------PS--R---
	Cand. Thermochlorobact. aerophilum	RFM23383.1	---H------IV		---------PS--R---
	Chlorobium sp. 445	PIO47072.1	Q--K-H--K-IV		---------PS--R---
Larger *Ignavibacteriae* Clade	*Melioribacter roseus*	WP_014857420.1	---N-ELNK-LL		---------I---K---
	Ignavibacterium album	HFI91396.1	-V-N-E-NKA-L		---------LS--LI--
	Melioribacteraceae bacterium	MCF8241219	---N-E-NKALL		--------SIG--K---
	*Ignavi. bacterium CG_4_9_14_3_*	PJA95416	V-K-S--KALL		---------IG--K---
	Ignavi. bacterium GWC2_38_9	OGU63466	-V-K-N--KALL		---------I-Y-R---
	Ignavi. bacterium HGW	PKL82039	K-N-EKALL		---------I-Y-K---
	Ignavi. bacterium CHB3	MCE7857972	---N-ELNKAIL		---------IS--
Other Bacteria	*Prevotella copri*	WP_158463588	---N-EFGNKPL	F	---------IG--M---
	Alloprevotella tannerae	MBF0970273	---NV-LGKRPL	L	---------IG--K--
	Prevotella marshii	WP_006950262	---N--LGSRPL	L	---------IG--L---
	Marinilabilia rubra	WP_109263596	---NLELRKEPL	L	---------QM--K---
	Moheibacter sediminis	SMC71036	---N-ELPKFPL	L	-------S-PP--R---
	Alkaliflexus imshenetskii	WP_044117808	---N-ELRKEPL	L	---------HM--S---
	Cryomorphaceae bacterium	HAP71276	---D-E-GKFPL	L	-------S-PP--K---
	Elizabethkingia meningoseptica	WP_073552649	---N-ELPDFPL	L	-------S-PP--K---
	Epilithonimonas caeni	WP_027384989	---N-ELPDFPL	L	-------S-PP--K---
	Epilithonimonas hungarica	WP_089873071	---N-ELPDFPL	L	-------S-PP--K---
	Elizabethkingia meningoseptica	WP_077564192	---N-ELPDFPL	L	-------S-PP--K---
	Muricauda aquimarina	WP_127140348	---N-ELPDFPL	L	-------S-PP--K---
	Muricauda oceanensis	WP_127136015	---N-ELPDFPL	L	-------S-PP--K---
	Tamlana sedimentorum	WP_044631505	---N-ELPDFPL	L	-------S-PP--K---
	Kaistella solincola	WP_039343339	---N-ELPEFPL	L	-------S-PP--K---
	Chryseobacterium salivictor	WP_133438697	---N-ELPEFPL	L	-------S-PP--K---
	Mesoflavibacter sabulilitoris	WP_106680957	---N-ELPEFPL	L	-------S-PP--K---
	Prevotella melaninogenica	WP_120174414	---T--LGERPL	F	---------IG--KM--
	Myroides profundi	AJH16529	---N-ELPDHPL	L	-------S-PP--R---

Figure S48

Partial sequence alignment of the protein tRNA dihydrouridine synthase DusB, showing a 1 aa deletion (boxed) that is specific for members of the order *Chlorobiales* and the “Larger Ignavibacteriae Clade”.
